# Elucidating the Multicomponent Reaction Pathway of 2‑Pyrrolidone Synthesis

**DOI:** 10.1021/acsomega.5c08141

**Published:** 2025-12-26

**Authors:** Alexander Dueñas-Deyá, Reyna Evelyn Cordero-Rivera, Mariano Martínez-Vázquez

**Affiliations:** Instituto de Química, Circuito Exterior s/n, Circuito de la Investigación Científica, Universidad Nacional Autónoma de México, 04510 Coyoacán, Ciudad de México

## Abstract

2-Pyrrolidone derivatives are valuable heterocycles with significant biological relevance, yet their synthesis through multicomponent reactions (MCRs) remains mechanistically ambiguous, particularly due to the difficulty of distinguishing them from isomeric aryl-substituted furanones. In this work, we combine electron impact mass spectrometry (EI-MS), Direct Analysis in Real Time mass spectrometry (DART-MS), and single-crystal X-ray diffraction to unambiguously confirm the exclusive formation of 2-pyrrolidone products in the reaction of anilines, benzaldehydes, and diethyl acetylenedicarboxylate. EI-MS provided a diagnostic fragmentation profile inconsistent with furanone structures, while X-ray analysis validated the pyrrolidone core. Time-resolved DART-MS enabled the detection of key long-lived intermediates–such as imines, hydrated alkyne adducts, and pyrrolidone-type species–supporting a stepwise mechanism involving acid-catalyzed imine formation, alkyne hydration, nucleophilic addition to an iminium ion, and final lactamization. Complementary experiments employing diethyl oxaloacetate, together with DFT calculations, further substantiated the pivotal role of alkyne hydration and the β-nucleophilic attack governing cyclization. Citric acid emerged as the most effective catalyst due to its dual activation of the aldehyde and iminium intermediates, while competing enamine formation rationalizes differences in isolated yields across the series. Overall, this study provides the first experimentally supported mechanism for this MCR and establishes a robust analytical framework for the structural and mechanistic elucidation of pyrrolidone-forming reactions.

## Graphical abstract

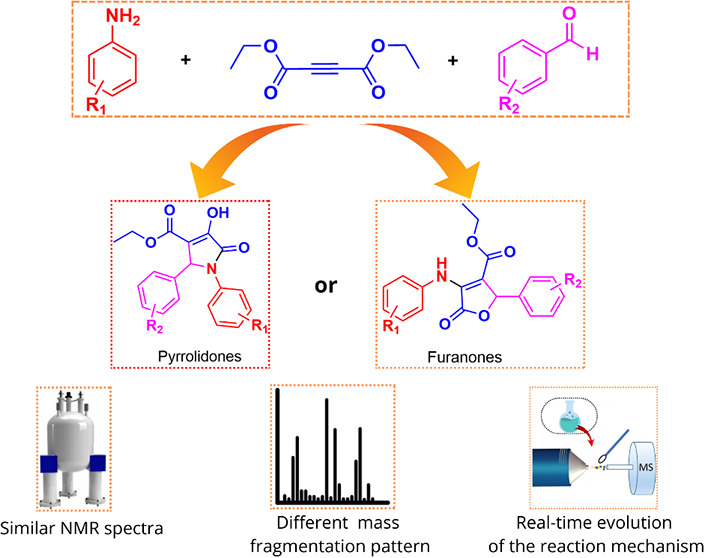

## 1 Introduction

2-Pyrrolidone groups are widely distributed in both pharmaceuticals and natural compounds. Notable examples of compounds featuring this moiety include cotinine, doxapram, ethosuximide, lactacystin, and indoprofen. Additionally, derivatives of 2-pyrrolidone display significant biological and pharmacological activities. These activities encompass action on the central nervous system, antibacterial properties, antimicrobial activity, anticancer effects, and anti-inflammatory properties.^1–8^ Consequently, it is crucial to investigate novel synthesis approaches for these five-membered heterocyclic structures, utilizing readily available starting materials and straightforward procedures^9^ (Figure 1).

**Figure 1:**
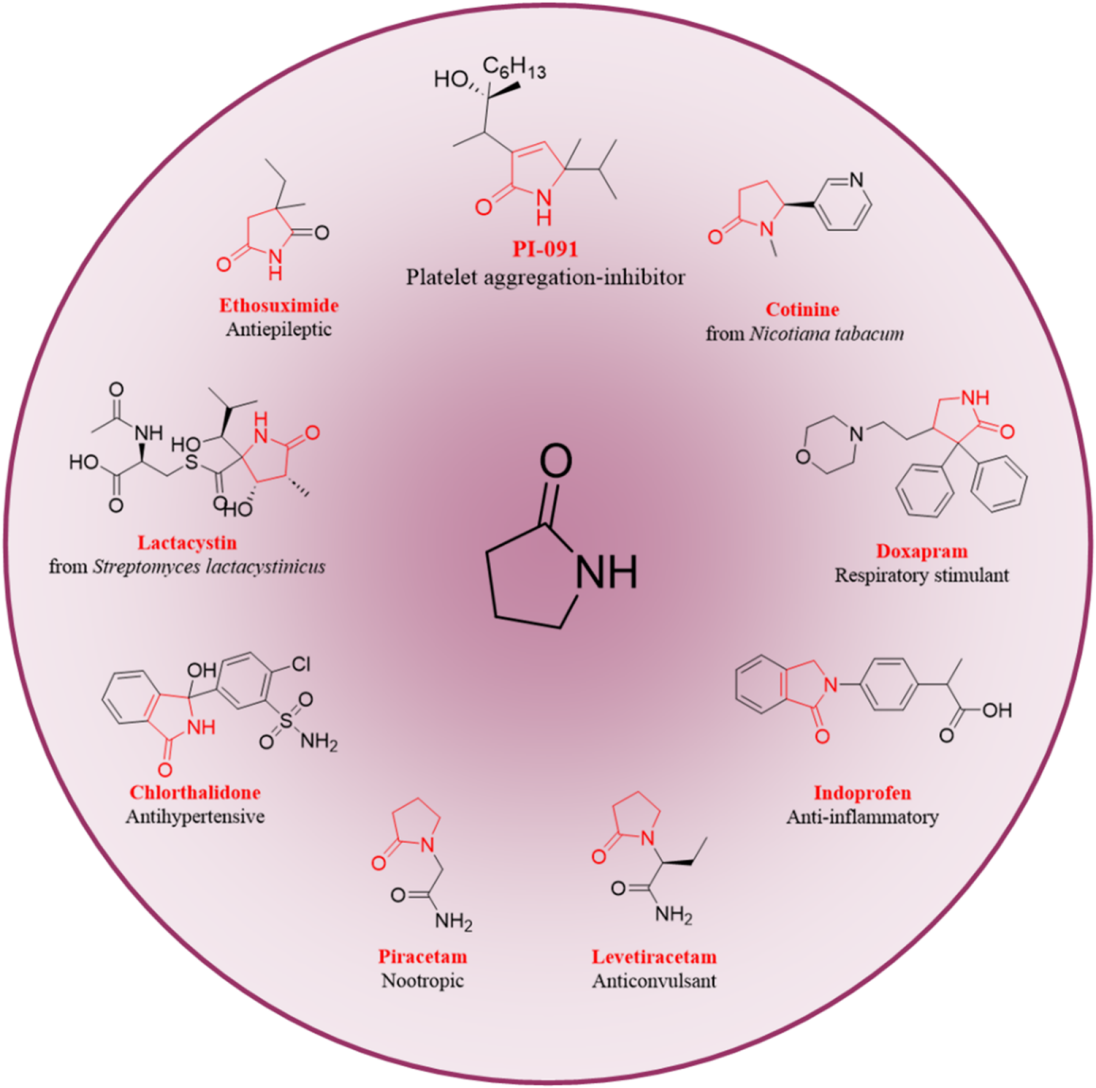
Chemical structures of some natural products and drugs containing 2-pyrrolidone moieties.

Due to the significant biological relevance of 2-pyrrolidones numerous methodologies have been developed to access functionalized these molecules, leveraging a diverse array of starting materials. These include anilines, benzaldehydes, dialkyl acetylenedicarboxylates, benzylamines, α-oxoaldehydes, and α-angelicalactone, among others.^9–11^ These reactions are typically catalyzed by various catalysts, including different types of acids and nanostructured materials, each offering specific advantages in selectivity, efficiency, and environmental impact.^12,13^

One of the procedures described for the synthesis of 2-pyrrolidones is Multicomponent Reactions (MCRs). It represents a powerful strategy in organic synthesis, characterized by their ability to simultaneously incorporate multiple starting materials into a single reaction vessel to produce complex products in a single step. This one-pot process is not only atom-economic, meaning that essentially all the atoms from the starting materials are retained in the final product, but also highly step-efficient, as it eliminates the need for multiple reaction steps and purification procedures typically associated with traditional linear synthesis routes.^14^ Moreover, MCRs offer unparalleled exploration power in terms of chemical space. By enabling the rapid assembly of diverse molecular scaffolds from simple building blocks, these reactions facilitate the efficient exploration of vast regions of chemical space. This aspect is particularly advantageous in the context of drug discovery and materials science, where access to diverse compound libraries is crucial for identifying novel bioactive molecules or functional materials with desired properties.^15^

In the realm of organic synthesis, the intricate interplay between anilines, dialkyl acetylenedicarboxylates, and benzaldehydes has garnered considerable attention. Researchers have delved deep into the exploration of this reaction, aiming to unlock its potential in crafting novel heterocyclic compounds. However, despite extensive investigation, the precise outcome of this MCR has remained elusive. One of the intriguing aspects of this reaction is the diverse array of products that can potentially emerge. Among these, aryl-substituted furanones and 2-pyrrolidones stand out as the most frequently encountered, and varying the reaction conditions, one or another product can be obtained, with various reaction media found in the reports that include aqueous systems or alcohols as solvents (Scheme 1).^11,16,17^ These compounds, characterized by their unique structural motifs and versatile chemical properties, hold significant promise in various fields, ranging from pharmaceuticals to materials science.

**Scheme 1:**
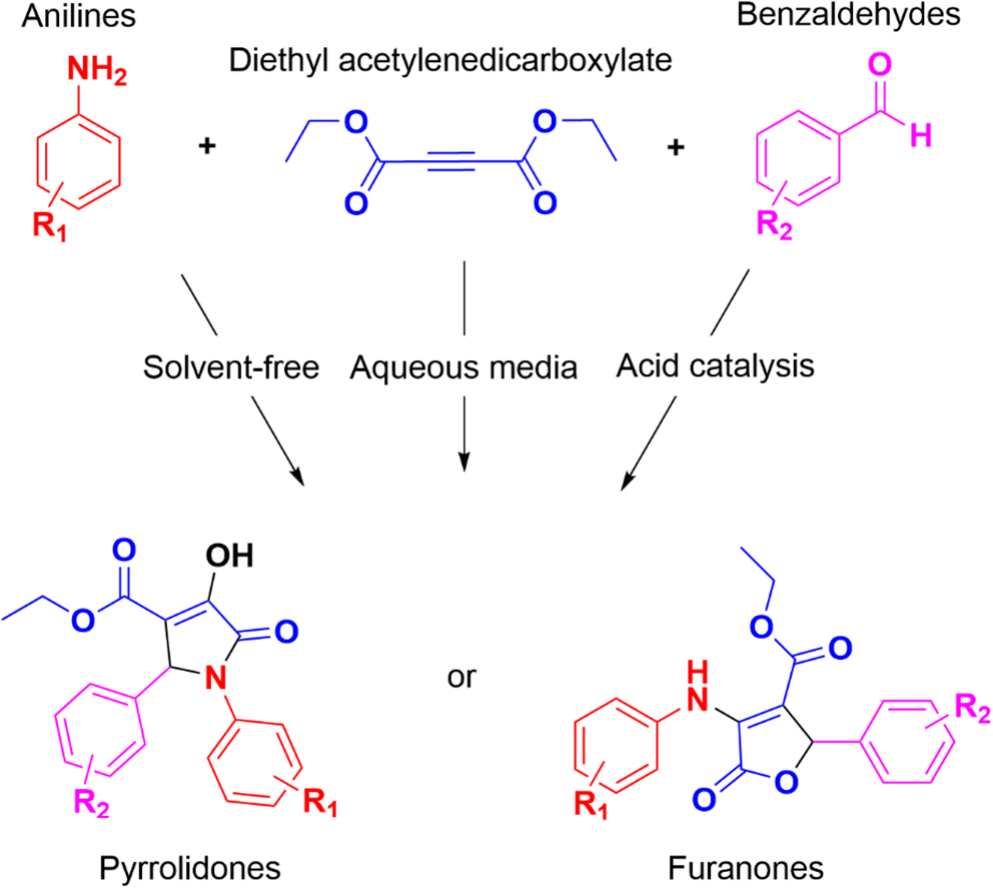
General Synthesis Pathway and Reaction Conditions: Solvent-free Conditions, Aqueous Media with β-Cyclodextrin and Citric Acid Catalysis in Ethanol Solution

The main challenge associated with the products obtained in this multicomponent reaction is their characterization, since both 2-pyrrolidones and furanones exhibit nearly identical Nuclear Magnetic Resonance (NMR) spectra. Consequently, distinguishing between these compounds with certainty has proven elusive. Several researchers have documented the identification of furanone-type structures, reporting their formation under different reaction conditions.^18,19^ Notably, this is a consequence of the highly similar NMR spectroscopic patterns exhibited by both furanones and pyrrolidones. In the absence of X-ray data to unequivocally validate the accuracy of the proposed outcomes, the identity of the synthesized compounds remains uncertain due to a gap in knowledge.

Thus, far, the most reliable method for achieving comprehensive and precise identification of these compounds has been through single-crystal X-ray diffraction. However, this technique is not easily accessible to many researchers due to its complexity, resource-intensive nature and the requirement of a high-quality single crystal.

Due to the ambiguity in the products obtained from this multicomponent reaction, there are no reports validating proposed reaction mechanisms. Understanding stepwise reaction mechanisms is crucial for sustainable development and precise chemical reactions control. Traditional empirical approaches are insufficient for studying new reactions with unexpected species and mechanisms.^20^ Spectroscopic techniques like UV/vis, IR, and NMR help monitor reactions but struggle with detecting fleeting intermediates. Direct Analysis in Real Time - Mass spectrometry (DART-MS) offers powerful structural analysis of multiple analytes, making it ideal for reaction monitoring.^21,22^

This study delves into the characterization of the products resulting from the chemical reaction involving anilines, dialkyl acetylenedicarboxylate, and benzaldehydes across diverse reaction environments. By examining the outcomes of these reactions under different conditions, the research aims to elucidate the intricate details of the chemical transformations involved. Moreover, the investigation extends to unraveling the underlying mechanism of the reaction using mass spectrometry in real time (DART), focusing on the selective formation of various reactive intermediate species. Through careful analysis of these intermediates, the study seeks to uncover the pathways and dynamics governing the reaction process, offering valuable insights into its mechanism. Furthermore, the research highlights the utility of electron impact mass spectrometry as a powerful analytical tool in this context. By leveraging this technique, researchers can effectively distinguish between the different compounds involved in the reaction, facilitating accurate identification and characterization.

## 2 Results and Discussion

### 2.1 Characterization of Products Obtained

The interaction between anilines, dialkyl acetylenedicarboxylate, and benzaldehydes is known to yield a diverse array of products,^13,23^ with the specific outcomes heavily influenced by the choice of solvent and catalyst. First, we explored the solvent-free route, which offers insights into the intrinsic reactivity of the reactants in the absence of any external solvent. This approach allows for a direct examination of the interactions between the reactants, potentially leading to unique product formations. Second, we investigated the reaction in an aqueous medium with the inclusion of β-cyclodextrin. By utilizing β-cyclodextrin as a molecular host, we aimed to create a confined environment that could selectively stabilize certain reaction intermediates or enhance the regioselectivity of the reaction, thus influencing the product distribution. Lastly, we examined the impact of acid catalysis in ethanol, leveraging the catalytic properties of acids to facilitate specific reaction pathways and modulate the product spectrum. The choice of ethanol as the solvent provides a polar environment conducive to the activation of reactants and the stabilization of reactive intermediates.

Table 1 illustrates the ^1^H and ^13^C NMR data of the products obtained under the three distinct reaction routes investigated. Remarkably, Table 1 shows the similarity among the three spectra according to the chemical shifts of the protons, suggesting that regardless of the specific conditions employed, the resulting product remains consistent across all three cases. The data derived from the ^13^C NMR spectra, coupled with the insights gained from two-dimensional experiments, provided further validation of this hypothesis (See Supporting Information). Moreover, the difficulty of unambiguously assigning the observed signals to either a pyrrolidone-type structure or its furanone-type isomer is evident, as their high structural similarity results in a very similar set of signals.

**Table 1:**
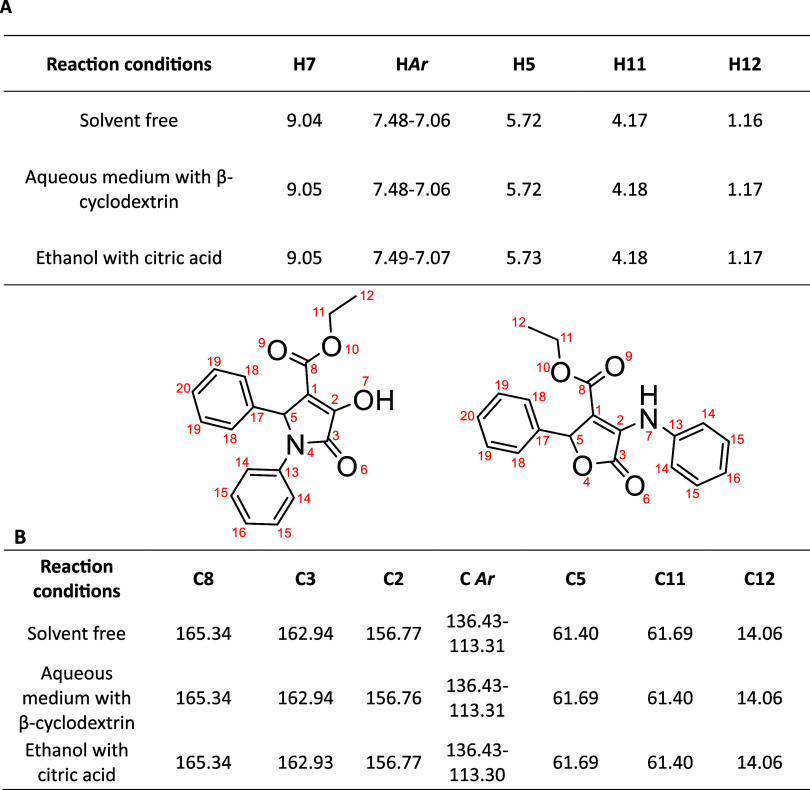
Chemical Shifts (δ, ppm) in ^1^H-NMR (A) and ^13^C-NMR (B) of the Products Obtained from the Reaction Involving Aniline, Benzaldehyde and Diethyl Acetylenedicarboxylate, under Different Reaction Conditions

### 2.2 Mass Spectrometry Characterization

While the multicomponent reaction between anilines, diethyl acetylenedicarboxylates, and benzaldehydes may lead, in principle, to the formation of either 2-pyrrolidones or furanones, unambiguous structural differentiation between these possibilities remains a significant analytical challenge. Nuclear magnetic resonance (NMR) spectroscopy, though a powerful tool, failed to resolve this ambiguity due to the striking similarity in chemical shifts and coupling patterns exhibited by both structural types.

To address this limitation, we employed electron impact mass spectrometry (EI-MS) to examine the fragmentation behavior on the synthesized compounds. Table 2 shows the prominent ions observed in the electron impact mass spectra for the molecules synthesized. Notably, the fragmentation patterns observed were inconsistent with a furanone core: key ions corresponding to furanone-derived cleavage were entirely absent, while diagnostic peaks uniquely attributable to pyrrolidone structures were consistently present. These include prominent signals associated with cleavage at the lactam ring, loss of the −COOEt group (M–73), and subsequent rearrangements that would not be feasible in a furanone scaffold. Thus, the mass spectral data not only provide compelling evidence for the formation of pyrrolidones but also effectively rule out the presence of furanone isomer in the reaction products.

**Table 2:** Ions Detected by Electron Impact Mass Spectrometry and Their Relative Abundance^a^

**compound**	**M** ^**+**^	**main fragments** *m*/*z*		
**1**	323	250	203	130
**2**	341	268	203	130
**3**	357	284	203	130
**4**	402/404	329/327	203	130
**5**	449	376	203	130
**6**	368	295	203	130
**7**	353	280	203	130
**8**	427	345	203	130
**9**	459	386	203	130
**10**	421/420	348/347	203	130
**11**	425	352	203	130
**12**	391	318	203	130
**13**	369	296	249	176
**14**	368	295	247	175
**15**	341	258	221	148
**16**	357	284	237	164
**17**	401/403	328/330	281	208/210
**18**	401/403	328/330	281	208/210
**19**	429	ND	ND	ND
**20**	353	280	233	160
**21**	383	310	263	190

a ND = Not detected.

When the molecule ionizes, electron loss can occur at two main sites: the ester carboxyl group or the carbonyl group in the ring. The location of ionization dictates the subsequent fragmentation patterns, shaping the molecule’s breakdown during analysis. The fragmentation process of synthesized pyrrolidones primarily involves the phenyl ring originating from benzaldehyde and implies the fragment derived from diethyl acetylenedicarboxylate. Interestingly, the involvement of the aniline ring in the fragmentation process is comparatively minimal.

Since the 203 *m*/*z* fragment in these derivatives could not arise from the loss of ethyl carboxylate at the C1 fragment, and the 130 *m*/*z* peak may originate from the 203 *m*/*z* fragment through the loss of ethyl carboxylate, the fragmentation pattern illustrated in Figure 2 is proposed. This proposal is supported, as derivatives **13–18** and **20–21** displayed the same pattern, although the values increased due to the substituents in the phenyl residue at C5.

**Figure 2:**
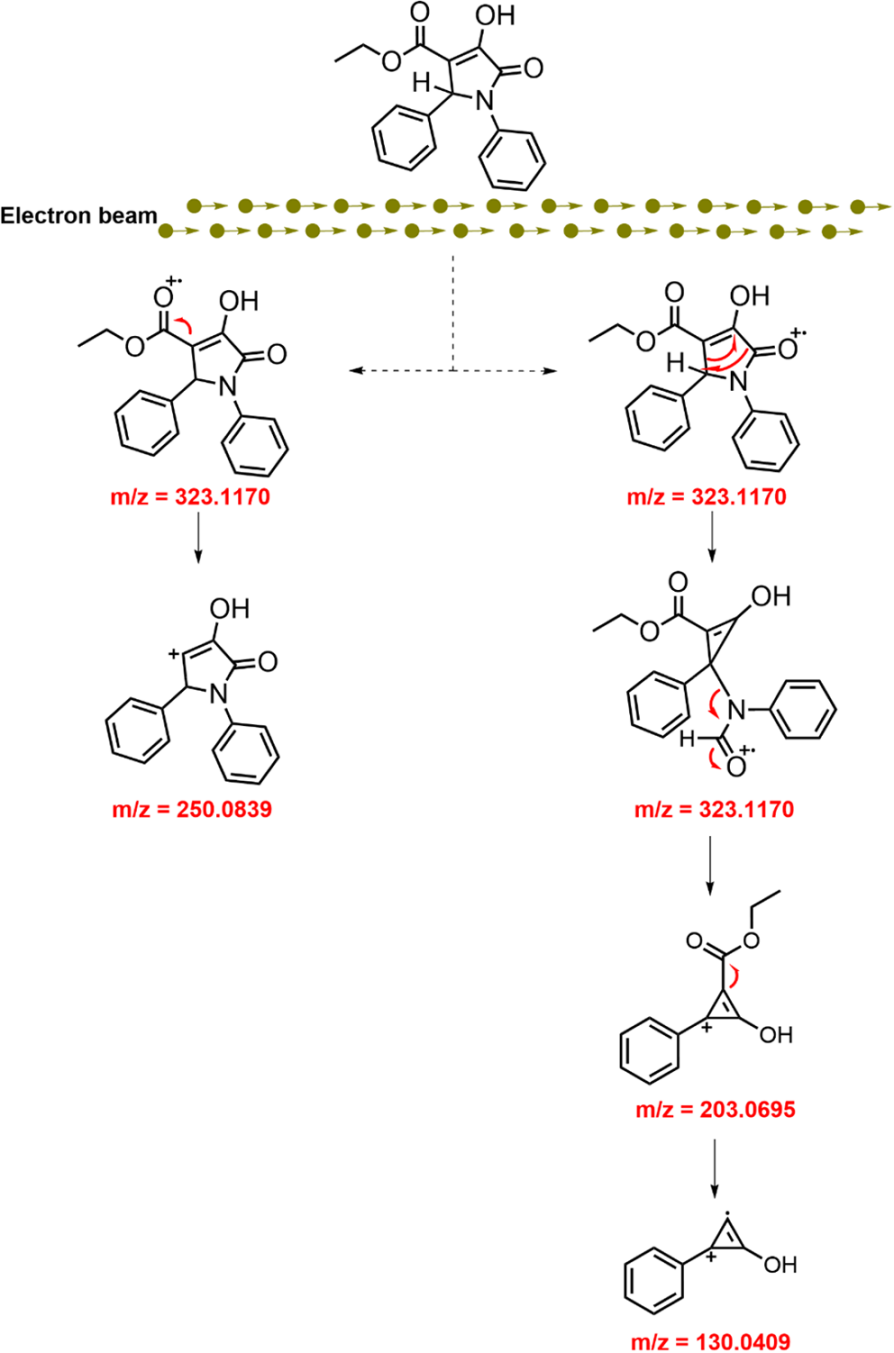
Plausible fragmentation mechanism by electron impact mass spectrometry of aryl pyrrolidone structures. Additional details are given in Supporting Information.

For all synthesized molecules, the presence of the molecular ion was observable and generally of medium to high intensity, exhibiting a characteristic isotopic distribution corresponding to the determined molecular formula. In all analyzed molecules, a peak corresponding to M-73 was consistently observed, attributed to the loss of the ester residue from the structure. When substituents were present only on the aniline ring, the mass spectra exhibited highly consistent peaks, suggesting that the detected ions did not involve fragments derived from the aniline residue. Conversely, a greater diversity of signals was observed when the aniline ring was unsubstituted, and the substituents were introduced on the aromatic ring originating from benzaldehyde.

The presence of these distinctive peaks serves as a hallmark feature indicative of an aryl-substituted pyrrolidone. This unique fingerprint makes electron impact mass spectrometry an indispensable method for distinguishing aryl-substituted pyrrolidones from aryl-substituted furanones. Additionally, we were able to obtain suitable single crystals of derivatives **2**, **9**, **12**, and **20** for X-ray diffraction analysis, as shown in Figure 3. This structural confirmation not only supported our mass spectrometry findings but also provided unequivocal evidence of the pyrrolidone core in the final products. With this combined data, we confidently assigned the definitive structures of the synthesized compounds, as revealed in Figure 4.

**Figure 3:**
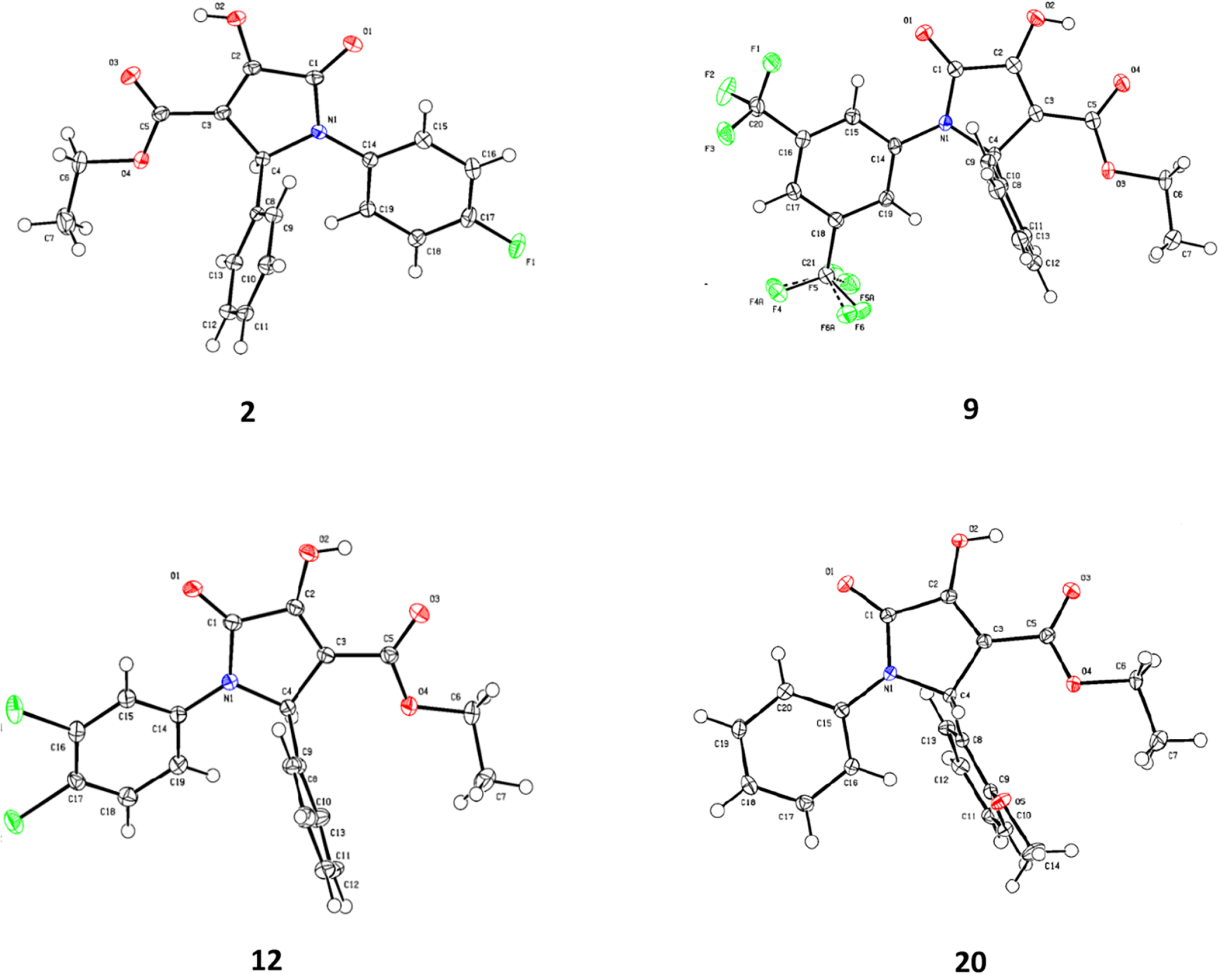
X-ray structures of compounds **2**, **9, 12** and **20.**.

**Figure 4:**
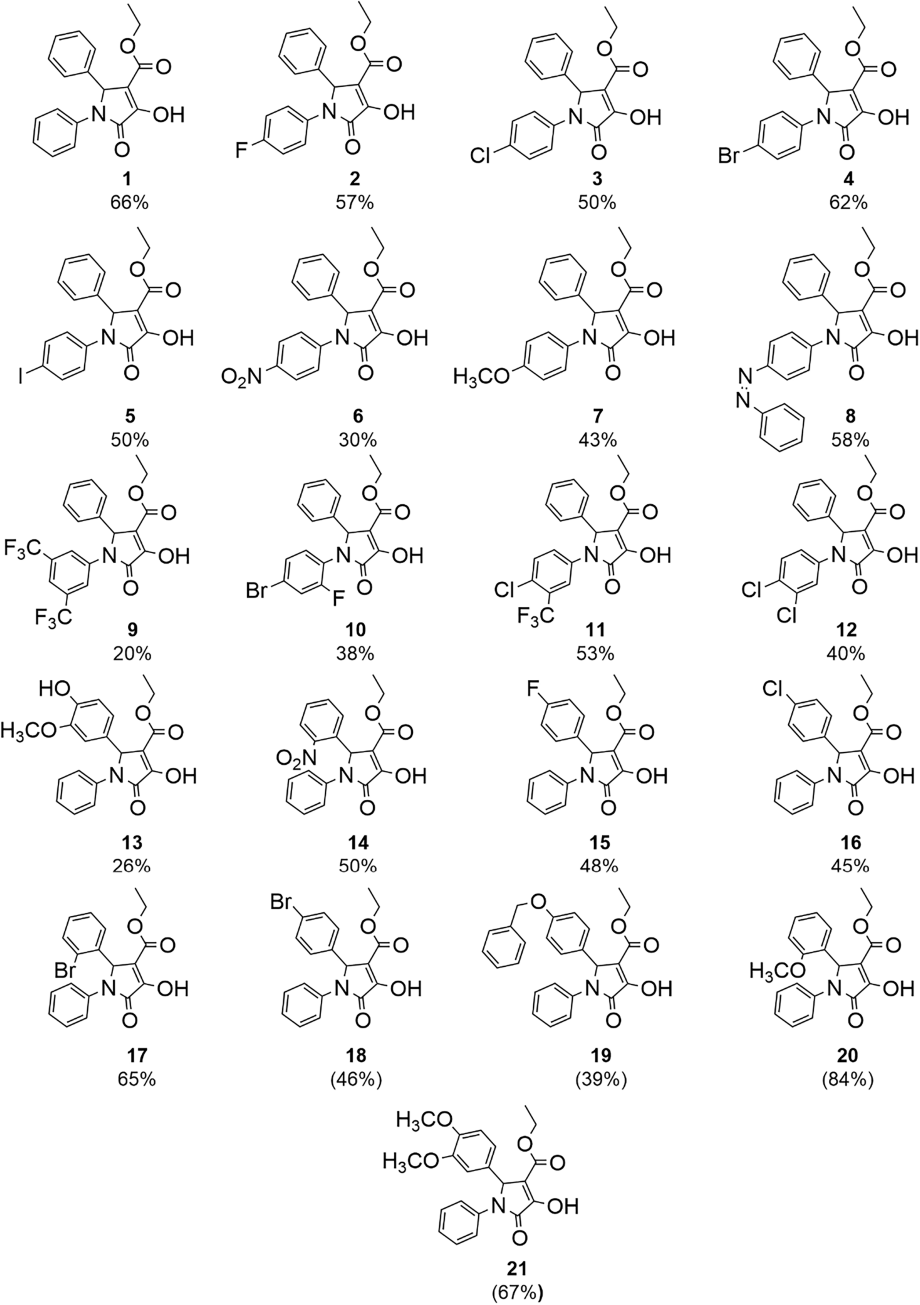
Compounds synthesized with pyrrolidone-like structures *via* citric acid catalysis.

### 2.3 Investigation of Reaction Mechanism

Despite extensive investigation on the multicomponent reaction involving anilines, benzaldehydes, and diethyl acetylenedicarboxylate, the precise mechanistic pathways leading to a pyrrolidone-type structure remain elusive. Several authors have proposed speculative reaction mechanisms that suggest the formation of furanone or 2-pyrrolidone-type structures.^13,24^ However, these proposed mechanisms lack experimental support. Consequently, there is a need to further explore and validate these mechanisms through experimental evidence.

The formation of the 2-pyrrolidone-type product was confirmed by X-ray and mass spectrometry; therefore, a study of the possible reaction mechanism underlying its formation was conducted. For this purpose, we utilized DART-MS (Direct Analysis in Real Time Mass Spectrometry), an analytical technique renowned for its simplicity and the absence of sample preparation requirements.^25^ These features make it an invaluable tool for identifying potential intermediates along the reaction pathway leading to 2-pyrrolidone formation, thereby enhancing our understanding and facilitating the optimization of this synthetic process.

However, it is essential to note a key limitation of this methodology: manual sampling intervals exceeded 10 min due to the offline detection system. This time constraint means that any highly transient or short-lived intermediates in the reaction cascade would likely have entirely reacted before analysis, preventing their observation. Therefore, our mechanistic discussion focuses exclusively on the long-lived species that were sufficiently stable for DART-MS detection. For a comprehensive kinetic analysis of all transient species, more advanced real-time in situ mass spectrometry techniques could address this challenge.^26,27^

Spectrometric analysis was initially conducted using the solvent-free reaction of compound **1** as a model. For the study, aliquots were taken from the reaction medium at defined time intervals, and the samples were quickly introduced into the ion stream of the mass spectrometer. This method enabled a temporal evaluation of the reaction’s progression, offering valuable insights into the intermediates and the mechanism’s evolution in real time.

The data presented in Table 3 and Figure 5 highlight the critical peaks detected during the DART-MS analysis of the reaction mixture. At the initial reaction time of 0 min, three prominent signals were observed at *m*/*z* = 182.09514, 264.12358, and 352.15369 (Peaks A, B, and C). These signals can be correlated with the molecular compositions of intermediate and potential reaction products based on the reactants and plausible chemical transformations occurring in the reaction medium. The signal at *m*/*z* = 182.09514 (Peak A) corresponds to a molecular formula of C_13_H_11_N (Detected as [M + H]^+^). It is attributed to the formation of the imine, a key intermediate, resulting from the condensation reaction between benzaldehyde and aniline. This imine serves as a precursor for subsequent transformations during the mechanism. The signal at *m*/*z* = 264.12358 (Peak B) aligns with a molecular formula of C_14_H_17_NO_4_ (Detected as [M + H]^+^). It is associated with the enamine intermediate, formed through the reaction between diethyl acetylenedicarboxylate and aniline. The formation of this intermediate indicates the nucleophilic addition of aniline to the activated alkyne, further demonstrating the system’s reactivity under the reaction conditions. However, the enamine results in a secondary product of a parallel reaction, since there is no logical pathway leading to the final product of the reaction from this intermediate. The peak at *m*/*z* = 352.15369 (Peak C) corresponds to a molecular formula of C_21_H_19_NO_3_ (Detected as [M-H_2_O+H]^+^). This compound likely represents an addition product formed by the reaction of hydrated diethyl acetylenedicarboxylate with the imine previously generated. As the reaction progresses and longer reaction times are achieved, additional signals emerge in the spectrum, indicative of further product evolution. A signal at *m*/*z* = 324.12254 (Peak D) is attributed to the formation of a pyrrolidone-type product, which aligns with a molecular formula of C_19_H_17_NO_4_. The 2-pyrrolidone structure indicates a critical step in the reaction, involving cyclization or rearrangement pathways, thereby stabilizing the product in its final form. A signal at *m*/*z* = 647.21999 (Peak E) is assigned to a molecular formula of C_38_H_34_N_2_O_8_, representing the dimerization product of the 2-pyrrolidone (detected as [2M+H]^+^). Notably, this dimerization does not arise from the reaction medium but forms *in situ* within the mass spectrometer. This phenomenon implies that the reaction conditions inside the spectrometer, such as energy collisions or ionization processes, facilitate the formation of this dimeric species. The same analysis was subsequently performed for the reaction carried out in ethanol with citric acid. Remarkably, the results were very similar, indicating the presence of the same reaction intermediates under these conditions, as indicated in Figure 6.

**Figure 5:**
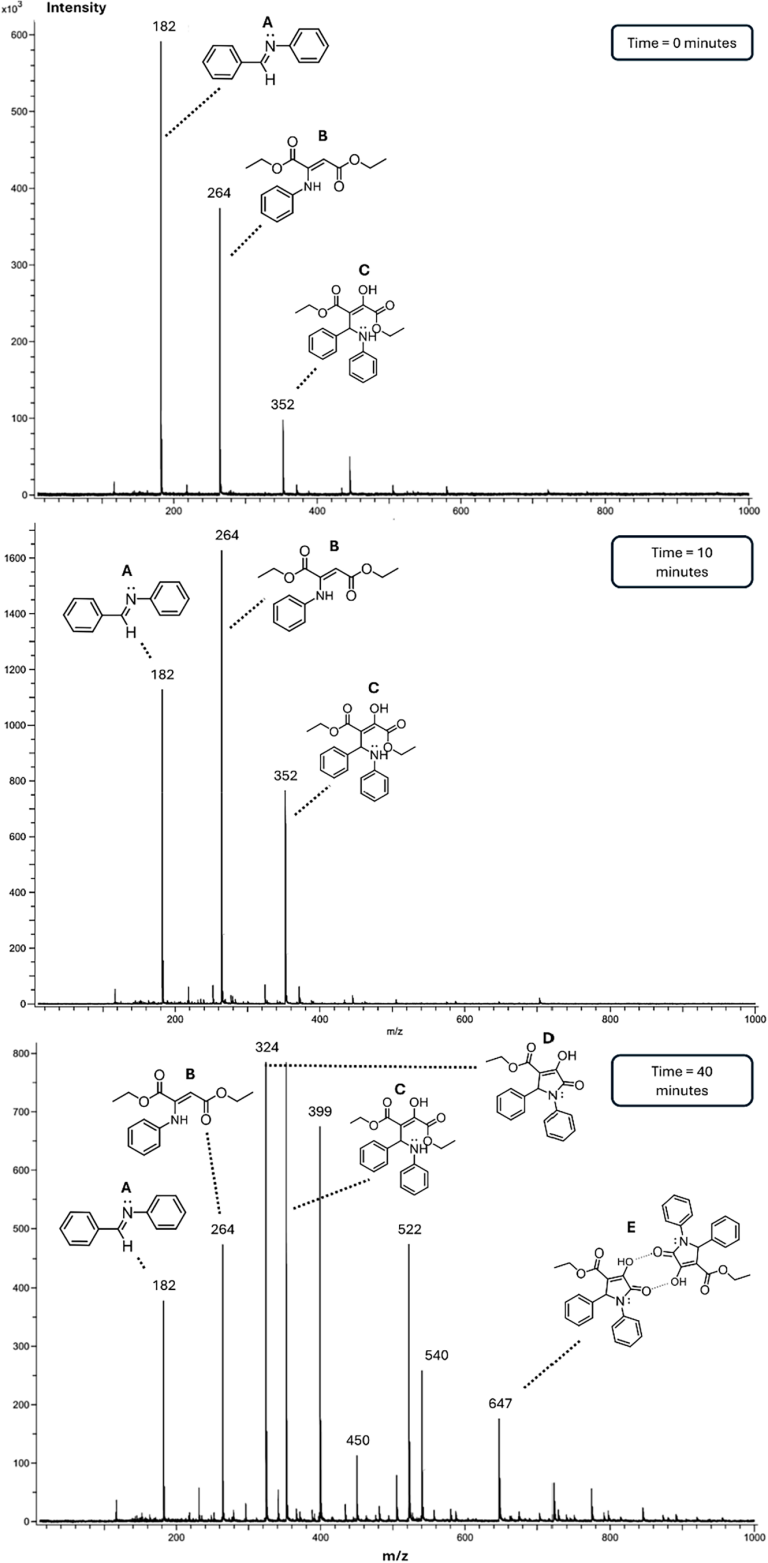
DART mass spectra in positive mode of solvent-free reaction mixtures at various time intervals, showing the disappearance of intermediate signals and the gradual emergence of product signals as the reaction progresses.

**Figure 6:**
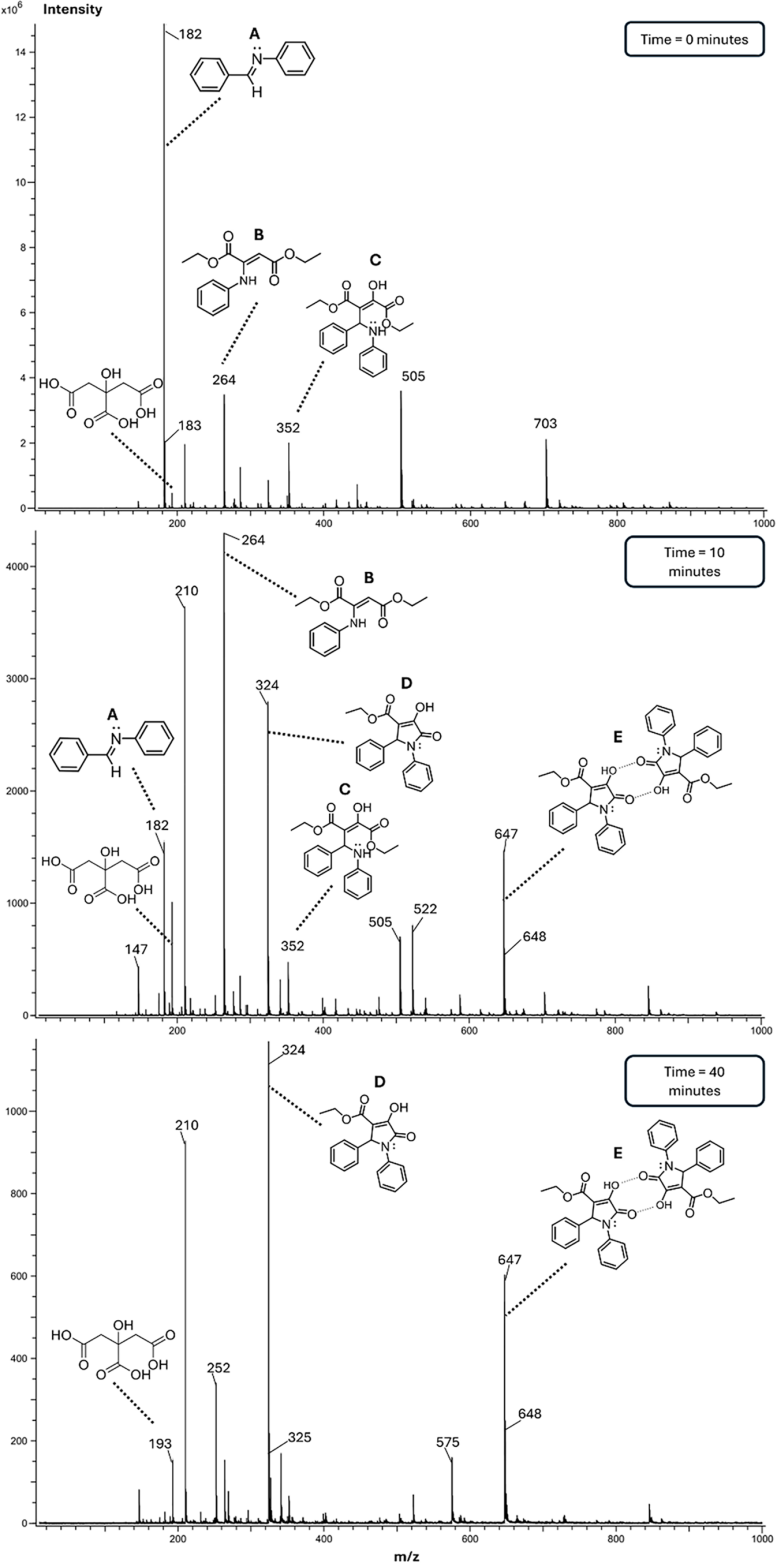
DART mass spectra in positive mode of reaction mixtures in ethanol with citric acid at various time intervals, showing the disappearance of intermediate signals and the gradual emergence of product signals as the reaction progresses.

**Table 3:** Important Peaks in the Mass Spectrum with DART-Positive Mode of the Reaction Mixture

ion	molecule	detected as	mass calculated	mass observed	error (ppm)
A	C_13_H_11_N	[A+H]^+^	182.09697	182.09514	–10.08
B	C_14_H_17_NO_4_	[B+H]^+^	264.12358	264.12246	–4.24
C	C_21_H_19_NO_3_	[C–H_2_O+H]^+^	352.15488	352.15369	–3.39
D	C_19_H_17_NO_4_	[D+H]^+^	324.12358	324.12254	–3.23
E	C_38_H_34_N_2_O_8_	[2D+H]^+^	647.21776	647.21999	3.44

The analysis of the species detected in the reaction medium supports a mechanism initiated by an acid-catalyzed condensation between the aldehyde and aniline, forming an imine and releasing water as a byproduct. The imine is then protonated to produce an iminium ion. Importantly, the water produced in situ promotes electrophilic addition to the triple bond of diethyl acetylenedicarboxylate, a key hydration step that triggers the subsequent cascade reaction.

Compelling evidence for this transformation comes from experiments where diethyl acetylenedicarboxylate was replaced by diethyl oxaloacetate sodium salt, a plausible downstream intermediate and the expected product of alkyne hydration (Scheme 2). Under these conditions, the reaction efficiently produces pyrrolidone **1**, aligning with the proposed mechanistic pathway (see Experimental Section and Supporting Information). This result strongly indicates that hydration of the alkyne moiety is a crucial prerequisite for cyclization to occur.

**Scheme 2:**
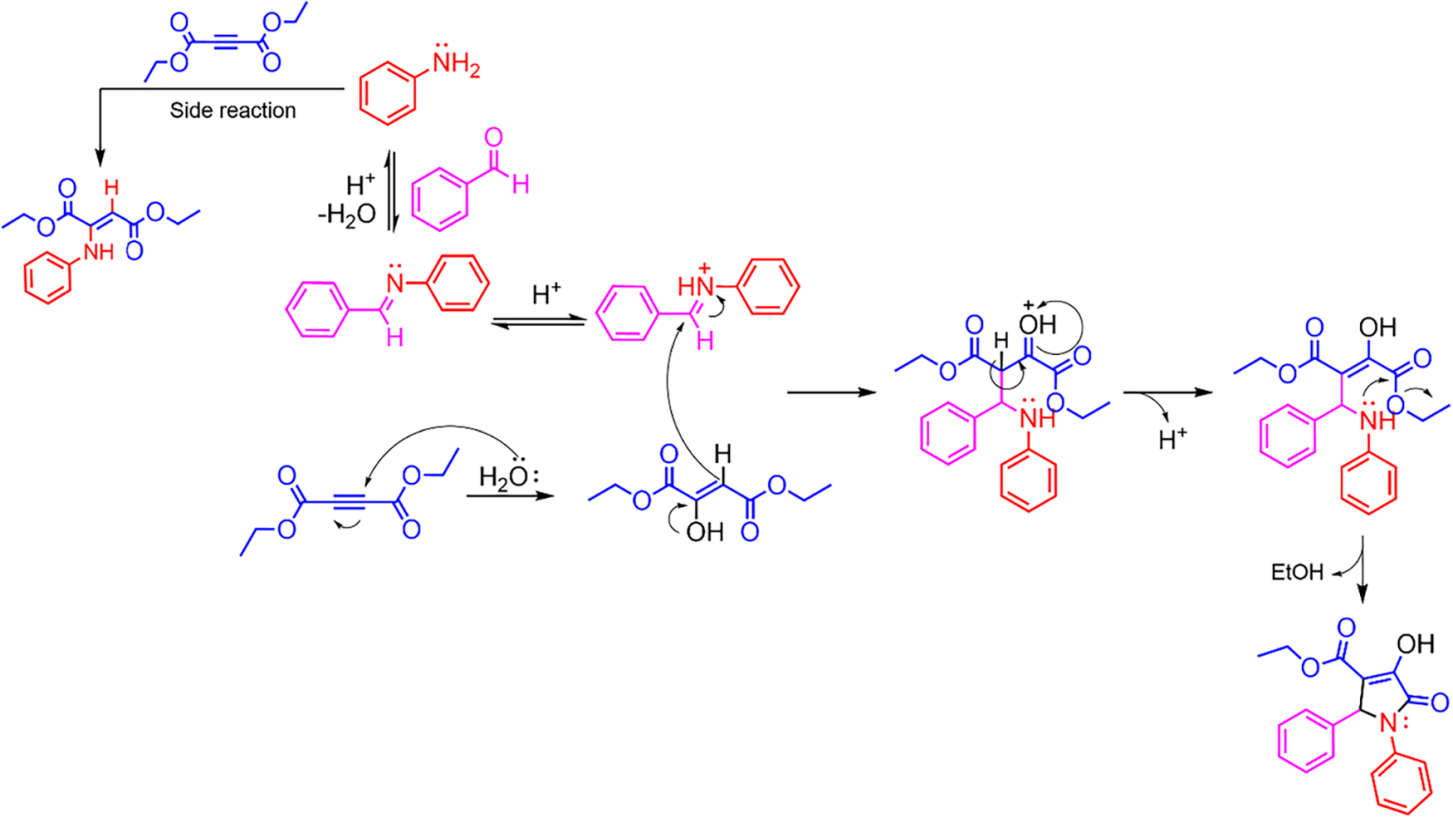
Proposed Reaction Mechanism for the Synthesis of Pyrrolidones from Anilines, Diethyl Acetylenedicarboxylate, and Benzaldehydes in an Acidic Medium

Although no signal corresponding to diethyl oxaloacetate was detected by DART-MS in the original reaction mixture, likely due to its high reactivity and short lifetime, the electronic structure of this species offers additional mechanistic insight. To understand the electronic features of this intermediate, density functional theory (DFT) calculations were performed using Gaussian 16. Geometry optimization and partial charge analysis were conducted at the M06–2X-D3/def2-TZVP level of theory at 298.15 K. Mulliken population analysis revealed that, excluding hydrogens, several carbon atoms carry negative partial charges, with some even more negative than the β-carbon of the enol group. However, when the contributions of bonded hydrogens were included, only the β-carbon maintained an overall negative charge (−0.0217 au), while the other carbon atoms became positive, as shown in Figure 7. This redistribution occurs because the small positive charges on hydrogen atoms partially offset the electron density on adjacent carbons, leaving the β-carbon as the only site with a true buildup of electronic density. This electron-rich site is well positioned to act as a nucleophile toward the electrophilic iminium ion previously formed, resulting in the formation of a neutral intermediate. These intermediate features have an amino group at the γ-position relative to one of the ester groups. Under the acidic reaction conditions, activation of the ester carbonyl enables intramolecular nucleophilic attack by the γ-amino group, promoting lactamization and ultimately producing a five-membered pyrrolidone ring (Scheme 2).

**Figure 7:**
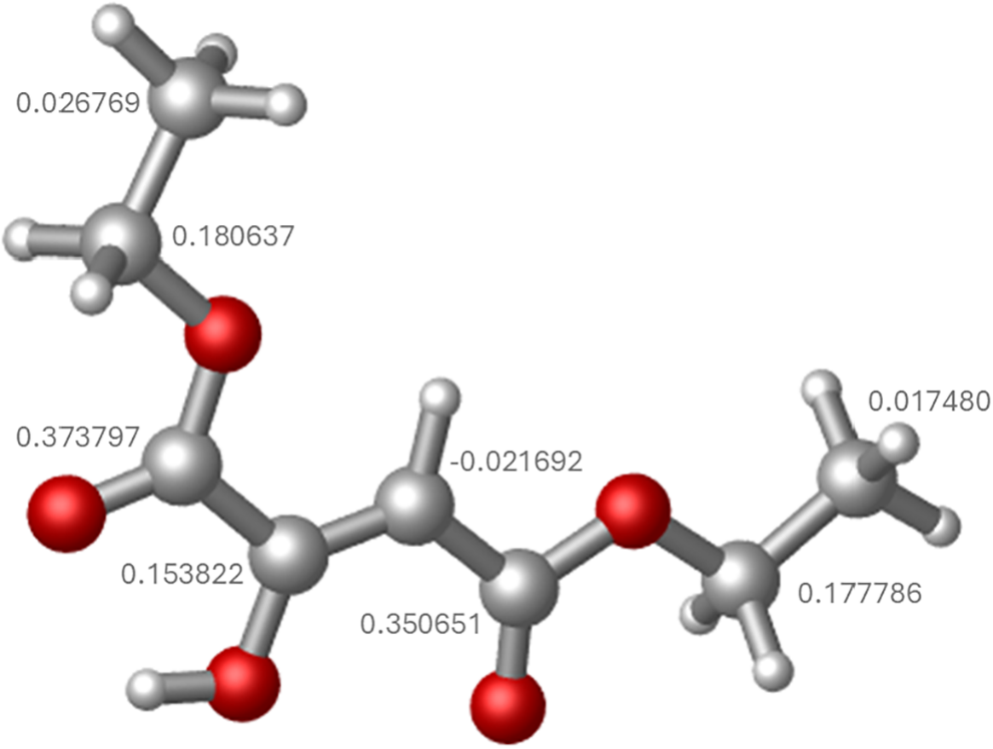
Optimized geometry of the intermediate obtained at the M06–2X-D3/def2-TZVP level of theory at 298.15 K, showing the distribution of partial charges on carbon atoms. Mulliken population analysis indicates that, after accounting for the contribution of bonded hydrogens, only the β-carbon of the enol group retains a negative partial charge (−0.021692 au), whereas the remaining carbon atoms become positively charged.

Further evidence supporting the critical role of water comes from control experiments in which the reaction was carried out in the presence of molecular sieves as a dehydrating agent (Experimental details in Supporting Information). Under these conditions, the expected pyrrolidone product was not formed, indicating that the water generated during the imine formation is essential for activating diethyl acetylenedicarboxylate and driving the subsequent cascade (Scheme 3).

**Scheme 3:**
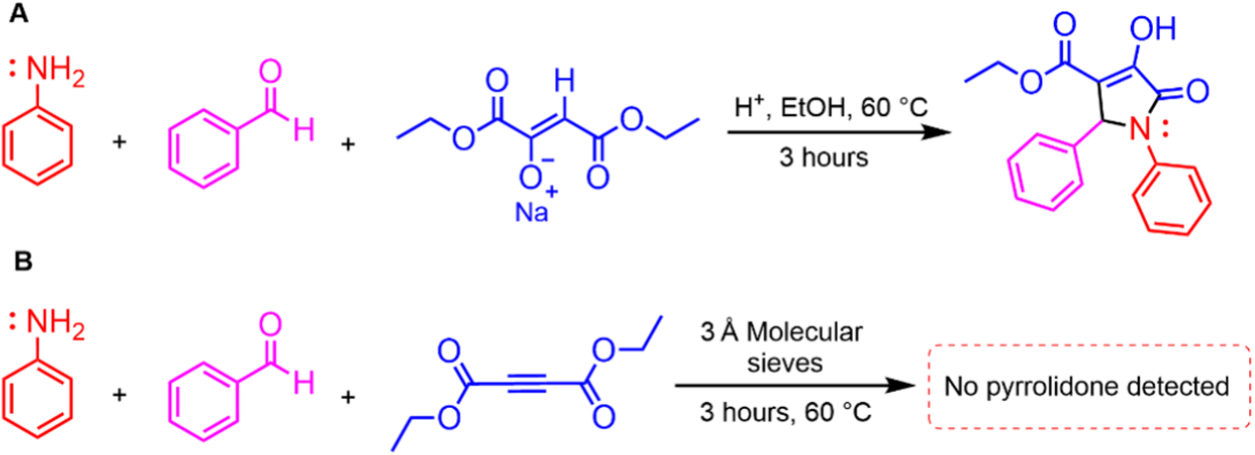
Water Influence Experiments^a^

a (A) In the presence of diethyl oxalacetate sodium salt, the reaction proceeds to the formation of a pyrrolidone-like product. (B) The use of molecular sieves, which retain water molecules, prevents the formation of the pyrrolidone-like product.

Overall, this stepwise mechanism illustrates the orchestrated interplay between acid-catalyzed imine formation, electrophilic addition to an alkyne, nucleophilic attack, and cyclization. The final product is a 2-pyrrolidone derivative, consistent with the observed reactivity and spectrometric data.

It is important to highlight that for compound **1**, the citric acid-catalyzed method proved to be the most efficient, with an isolated yield of 65%. In contrast, the aqueous and solvent-free methods consistently produced significantly lower yields, 54 and 15%, respectively. This stark difference is mainly due to the bifunctional catalytic role of citric acid, which overcomes the kinetic challenges of this multicomponent reaction. First, the acid activates the benzaldehyde carbonyl group through protonation via strong hydrogen bonding, increasing the ate-determining imine formation with the aniline. Importantly, the acid can also protonate the resulting imine, forming a highly electrophilic iminium species. This iminium ion is much more reactive toward nucleophilic attack by the diethyl oxalacetate, aiding the following cyclization step to create the 2-pyrrolidone ring. Second, the acidic environment helps remove the −OH group via water release during condensation, which is crucial for shifting the reaction equilibrium toward the desired product. The lack of this dual activation and equilibrium control in the noncatalyzed methods explains the sharp drop in overall isolated yields.

Once the efficiency of the catalyzed method was established, the variability in yields within this series can be attributed to the presence of a competing pathway in which the aniline can react either with the benzaldehyde to form the imine intermediate, which is in equilibrium with the starting materials due to acid conditions, or directly with the acetylenedicarboxylate to form the enamine. Only the imine pathway leads to the 2-pyrrolidone product, whereas the enamine does not evolve toward cyclization, thereby reducing the overall yield. The variability in isolated yields is directly linked to the competition between two parallel pathways involving the starting aniline. The desired pathway requires the formation of the imine intermediate with benzaldehyde. However, the aniline can also undergo a direct addition with the diethyl acetylenedicarboxylate to yield an enamine intermediate. Crucially, subsequent NMR analysis of the mother liquor confirmed that this enamine is stable under the reaction conditions and does not evolve toward the final 2-pyrrolidone product (See Supporting Information). Therefore, the enamine formation pathway represents a nonproductive, irreversible shunt that consumes part of the starting aniline. The observed yield reduction is a direct consequence of this competing reaction. In this context, electron-withdrawing substituents on the aniline ring generally decreased the formation of the 2-pyrrolidone product, consistent with their reduced nucleophilicity and diminished ability to participate in the imine-forming step. In contrast, electron-donating groups on the aniline favored the desired pathway, leading to higher yields. Substitution on the benzaldehyde ring showed a less pronounced influence; however, the highest yield (84%) was obtained for the derivative bearing an ortho-methoxy substituent. Although methoxy groups are electron-donating by resonance, their ortho orientation enables intramolecular interaction with the formyl hydrogen, thereby favorably polarizing the carbonyl and stabilizing the corresponding imine intermediate. This observation aligns with the behavior reported by Barber et al.,^28^ where ortho-methoxy-substituted pyridinecarboxaldehydes exhibited enhanced iminium stability and faster cyclization due to intramolecular hydrogen bonding. Consequently, the ortho-methoxy benzaldehyde likely promotes imine formation and directs the reaction toward the 2-pyrrolidone pathway, resulting in the highest isolated yield among the series.

## 3 Conclusions

The multicomponent reaction involving anilines, diethyl acetylenedicarboxylate, and benzaldehydes were examined to differentiate between the formation of two isomeric products: furanone-like or 2-pyrrolidone-like structures. Nuclear Magnetic Resonance characterization showed that distinguishing between these two isomeric structures was difficult. However, this uncertainty was resolved using single-crystal X-ray diffraction, which clearly confirmed the formation of 2-pyrrolidone-type structures. Additionally, detailed analysis with EI-MS enabled the development of a fragmentation mechanism for these molecules, demonstrating that electron bombardment mainly causes cleavage at the ring derived from the aldehyde. Furthermore, the DART-MS technique was used to explore the acid-catalyzed reaction mechanism leading to 2-pyrrolidone formation, highlighting the essential roles of the imine and water in the process.

## 4 Material and Methods

### 4.1 Chemicals

All chemical reagents used during the experiments were purchased from Sigma-Aldrich and were used without any further purification.

### 4.2 Instruments

Melting points were determined with a Fisher-Johns apparatus. The acquisition of the one- and two-dimensional NMR spectra was carried out on a Bruker Avance spectrometer (300 and 400 MHz). The Electronic Impact (EI) mass spectra of the synthesized compounds were acquired in a JMS-700 MStation spectrometer operating at an ionization potential of 70 eV. Single crystal X-ray data were acquired on a Bruker Apex II Duo diffractometer equipped with Mo (λ=0.71073 Å) X-ray source, see supporting data for additional information.

#### 4.2.1 Synthesis procedure

##### 4.2.1.1 Compound 1

Three reaction conditions frequently reported in the literature were reproduced, which are described below.

### 4.3 Solvent-Free Reaction

3.0 mmol of aniline (0.27 mL, 1 equiv), 3.0 mmol of diethyl acetylenedicarboxylate (0.48 mL, 1 equiv) and 3.0 mmol of benzaldehyde (0.38 mL, 1 equiv) were placed in a round-bottomed flask, heating the mixture at 60 °C for 2.5 h until the appearance of an insoluble solid, which was considered the end of the reaction. The semisolid precipitate was filtered and redissolved in ethyl acetate, precipitating the compound of interest with the careful addition of hexane. The solid obtained was filtered and recrystallized from hot ethanol (Scheme 1).

### 4.4 Reaction in Aqueous Media

The procedure previously described by Aburto et al. was followed.^29^ Briefly, 0.34 g of β-cyclodextrin was dissolved in 15.0 mL of distilled water. Subsequently, 3.0 mmol of aniline (0.27 mL, 1 equiv) were added until complete dissolution, followed by 3.0 mmol of diethyl acetylenedicarboxylate (0.48 mL, 1 equiv) and finally 3.0 mmol of benzaldehyde (0.38 mL, 1 equiv). The reaction was maintained at 60 °C for 24 h until the formation of a gummy precipitate was observed. The semisolid precipitate was filtered and redissolved in ethyl acetate, precipitating the compound of interest with the careful addition of hexane. The solid obtained was filtered and recrystallized from hot ethanol.

### 4.5 Acid Catalysis Reaction

The procedure previously reported by Ahankar et al. was followed with slight modifications.^11^ In brief, 3.0 mmol of aniline (0.27 mL, 1 equiv) was dissolved in 6 mL of ethanol, followed by 3.0 mmol of diethyl acetylenedicarboxylate (0.48 mL, 1 equiv), 3.0 mmol of benzaldehyde (0.38 mL, 1 equiv), and 2.0 mmol of citric acid monohydrate (0.42 g) were added to the mixture and heated at 60 °C for 3.0 h until the appearance of a solid dispersed in the medium. Once the reaction was completed, the solid obtained was filtered and recrystallized from hot ethanol.

#### 4.5.1 Compound **2**

3.0 mmol of 4-fluoroaniline (0.3 mL, 1 equiv) was dissolved in 6 mL of ethanol, followed by 3.0 mmol of diethyl acetylenedicarboxylate (0.48 mL, 1 equiv), 3.0 mmol of benzaldehyde (0.38 mL, 1 equiv), and 2.0 mmol of citric acid monohydrate (0.42 g) were added to the mixture and heated at 60 °C for 5.0 h until the appearance of a solid dispersed in the medium. Once the reaction was completed, the solid obtained was filtered and recrystallized from hot ethanol.

#### 4.5.2 Compound **3**

3.0 mmol of 4-chloroaniline (0.38 g, 1 equiv) was dissolved in 6 mL of ethanol, followed by 3.0 mmol of diethyl acetylenedicarboxylate (0.48 mL, 1 equiv), 3.0 mmol of benzaldehyde (0.38 mL, 1 equiv), and 2.0 mmol of citric acid monohydrate (0.42 g) were added to the mixture and heated at 60 °C for 5.0 h until the appearance of a solid dispersed in the medium. Once the reaction was completed, the solid obtained was filtered and recrystallized from hot ethanol.

#### 4.5.3 Compound **4**

3.0 mmol of 4-bromoaniline (0.52 g, 1 equiv) was dissolved in 6 mL of ethanol, followed by 3.0 mmol of diethyl acetylenedicarboxylate (0.48 mL, 1 equiv), 3.0 mmol of benzaldehyde (0.38 mL, 1 equiv), and 2.0 mmol of citric acid monohydrate (0.42 g) were added to the mixture and heated at 60 °C for 4.0 h until the appearance of a solid dispersed in the medium. Once the reaction was completed, the solid obtained was filtered and recrystallized from hot ethanol.

#### 4.5.4 Compound **5**

3.0 mmol of 4-iodoaniline (0.66 g, 1 equiv) was dissolved in 6 mL of ethanol, followed by 3.0 mmol of diethyl acetylenedicarboxylate (0.48 mL, 1 equiv), 3.0 mmol of benzaldehyde (0.38 mL, 1 equiv), and 2.0 mmol of citric acid monohydrate (0.42 g) were added to the mixture and heated at 60 °C for 5.0 h until the appearance of a solid dispersed in the medium. Once the reaction was completed, the solid obtained was filtered and recrystallized from hot ethanol.

#### 4.5.5 Compound **6**

3.0 mmol of 4-nitroaniline (0.41 g, 1 equiv) was dissolved in 6 mL of ethanol, followed by 3.0 mmol of diethyl acetylenedicarboxylate (0.48 mL, 1 equiv), 3.0 mmol of benzaldehyde (0.38 mL, 1 equiv), and 2.0 mmol of citric acid monohydrate (0.42 g) were added to the mixture and heated at 60 °C for 6.0 h until the appearance of a solid dispersed in the medium. Once the reaction was completed, the solid obtained was filtered and recrystallized from hot ethanol.

#### 4.5.6 Compound **7**

3.0 mmol of 4-methoxyaniline (0.36 g, 1 equiv) was dissolved in 6 mL of ethanol, followed by 3.0 mmol of diethyl acetylenedicarboxylate (0.48 mL, 1 equiv), 3.0 mmol of benzaldehyde (0.38 mL, 1 equiv), and 2.0 mmol of citric acid monohydrate (0.42 g) were added to the mixture and heated at 60 °C for 3.5 h until the appearance of a solid dispersed in the medium. Once the reaction was completed, the solid obtained was filtered and recrystallized from hot ethanol.

#### 4.5.7 Compound **8**

3.0 mmol of 4-phenylazoaniline (0.59 g, 1 equiv) was dissolved in 6 mL of ethanol, followed by 3.0 mmol of diethyl acetylenedicarboxylate (0.48 mL, 1 equiv), 3.0 mmol of benzaldehyde (0.38 mL, 1 equiv), and 2.0 mmol of citric acid monohydrate (0.42 g) were added to the mixture and heated at 60 °C for 5.0 h until the appearance of a solid dispersed in the medium. Once the reaction was completed, the solid obtained was filtered and recrystallized from hot ethanol.

#### 4.5.8 Compound **9**

3.0 mmol of 3,5-bis­(trifluoromethyl)­aniline (0.47 mL, 1 equiv) was dissolved in 6 mL of ethanol, followed by 3.0 mmol of diethyl acetylenedicarboxylate (0.48 mL, 1 equiv), 3.0 mmol of benzaldehyde (0.38 mL, 1 equiv), and 2.0 mmol of citric acid monohydrate (0.42 g) were added to the mixture and heated at 60 °C for 7.0 h until the appearance of a solid dispersed in the medium. Once the reaction was completed, the solid obtained was filtered and recrystallized from hot ethanol.

#### 4.5.9 Compound **10**

3.0 mmol of 4-bromo-2-fluoroaniline (0.57 g, 1 equiv) was dissolved in 6 mL of ethanol, followed by 3.0 mmol of diethyl acetylenedicarboxylate (0.48 mL, 1 equiv), 3.0 mmol of benzaldehyde (0.38 mL, 1 equiv), and 2.0 mmol of citric acid monohydrate (0.42 g) were added to the mixture and heated at 60 °C for 4.0 h until the appearance of a solid dispersed in the medium. Once the reaction was completed, the solid obtained was filtered and recrystallized from hot ethanol.

#### 4.5.10 Compound **11**

3.0 mmol of 4-chloro-3-(trifluoromethyl)­aniline (0.59 g, 1 equiv) was dissolved in 6 mL of ethanol, followed by 3.0 mmol of diethyl acetylenedicarboxylate (0.48 mL, 1 equiv), 3.0 mmol of benzaldehyde (0.38 mL, 1 equiv), and 2.0 mmol of citric acid monohydrate (0.42 g) were added to the mixture and heated at 60 °C for 4.5 h until the appearance of a solid dispersed in the medium. Once the reaction was completed, the solid obtained was filtered and recrystallized from hot ethanol.

#### 4.5.11 Compound **12**

3.0 mmol of 3,4-dichloroaniline (0.48 g, 1 equiv) was dissolved in 6 mL of ethanol, followed by 3.0 mmol of diethyl acetylenedicarboxylate (0.48 mL, 1 equiv), 3.0 mmol of benzaldehyde (0.38 mL, 1 equiv), and 2.0 mmol of citric acid monohydrate (0.42 g) were added to the mixture and heated at 60 °C for 3.5 h until the appearance of a solid dispersed in the medium. Once the reaction was completed, the solid obtained was filtered and recrystallized from hot ethanol.

#### 4.5.12 Compound **13**

3.0 mmol of aniline (0.27 mL, 1 equiv) was dissolved in 6 mL of ethanol, followed by 3.0 mmol of diethyl acetylenedicarboxylate (0.48 mL, 1 equiv), 3.0 mmol of 3-hydroxy-4-methoxybenzaldehyde (0.46 g, 1 equiv), and 2.0 mmol of citric acid monohydrate (0.42 g) were added to the mixture and heated at 60 °C for 4.5 h until the appearance of a solid dispersed in the medium. Once the reaction was completed, the solid obtained was filtered and recrystallized from hot ethanol.

#### 4.5.13 Compound **14**

3.0 mmol of of aniline (0.27 mL, 1 equiv) was dissolved in 6 mL of ethanol, followed by 3.0 mmol of diethyl acetylenedicarboxylate (0.48 mL, 1 equiv), 3.0 mmol of 2-nitrobenzaldehyde (0.46 g, 1 equiv) and 2.0 mmol of citric acid monohydrate (0.42 g) were added to the mixture and heated at 60 °C for 3.0 h until the appearance of a solid dispersed in the medium. Once the reaction was completed, the solid obtained was filtered and recrystallized from hot ethanol.

#### 4.5.14 Compound **15**

3.0 mmol of aniline (0.27 mL, 1 equiv) was dissolved in 6 mL of ethanol, followed by 3.0 mmol of diethyl acetylenedicarboxylate (0.48 mL, 1 equiv), 3.0 mmol of 4-fluorbenzaldehyde (0.32 mL, 1 equiv) and 2.0 mmol of citric acid monohydrate (0.42 g) were added to the mixture and heated at 60 °C for 3.0 h until the appearance of a solid dispersed in the medium. Once the reaction was completed, the solid obtained was filtered and recrystallized from hot ethanol.

#### 4.5.15 Compound **16**

3.0 mmol of aniline (0.27 mL, 1 equiv) was dissolved in 6 mL of ethanol, followed by 3.0 mmol of diethyl acetylenedicarboxylate (0.48 mL, 1 equiv), 3.0 mmol of 4-chlorobenzaldehyde (0.42 g, 1 equiv) and 2.0 mmol of citric acid monohydrate (0.42 g) were added to the mixture and heated at 60 °C for 3.5 h until the appearance of a solid dispersed in the medium. Once the reaction was completed, the solid obtained was filtered and recrystallized from hot ethanol.

#### 4.5.16 Compound **17**

3.0 mmol of aniline (0.27 mL, 1 equiv) was dissolved in 6 mL of ethanol, followed by 3.0 mmol of diethyl acetylenedicarboxylate (0.48 mL, 1 equiv), 3.0 mmol of 2-bromobenzaldehyde (0.55 g, 1 equiv) and 2.0 mmol of citric acid monohydrate (0.42 g) were added to the mixture and heated at 60 °C for 5.0 h until the appearance of a solid dispersed in the medium. Once the reaction was completed, the solid obtained was filtered and recrystallized from hot ethanol.

#### 4.5.17 Compound **18**

3.0 mmol of aniline (0.27 mL, 1 equiv) was dissolved in 6 mL of ethanol, followed by 3.0 mmol of diethyl acetylenedicarboxylate (0.48 mL, 1 equiv), 3.0 mmol of 4-bromobenzaldehyde (0.55 g, 1 equiv) and 2.0 mmol of citric acid monohydrate (0.42 g) were added to the mixture and heated at 60 °C for 4.5 h until the appearance of a solid dispersed in the medium. Once the reaction was completed, the solid obtained was filtered and recrystallized from hot ethanol.

#### 4.5.18 Compound **19**

3.0 mmol of aniline (0.27 mL, 1 equiv) was dissolved in 6 mL of ethanol, followed by 3.0 mmol of diethyl acetylenedicarboxylate (0.48 mL, 1 equiv), 3.0 mmol of 4-(benzyloxy)­benzaldehyde (0.64 g, 1 equiv) and 2.0 mmol of citric acid monohydrate (0.42 g) were added to the mixture and heated at 60 °C for 5.0 h until the appearance of a solid dispersed in the medium. Once the reaction was completed, the solid obtained was filtered and recrystallized from hot ethanol.

#### 4.5.19 Compound **20**

3.0 mmol of aniline (0.27 mL, 1 equiv) was dissolved in 6 mL of ethanol, followed by 3.0 mmol of diethyl acetylenedicarboxylate (0.48 mL, 1 equiv), 3.0 mmol of 2-methoxybenzaldehyde (0.41 g, 1 equiv) and 2.0 mmol of citric acid monohydrate (0.42 g) were added to the mixture and heated at 60 °C for 4.5 h until the appearance of a solid dispersed in the medium. Once the reaction was completed, the solid obtained was filtered and recrystallized from hot ethanol.

#### 4.5.20 Compound **21**

3.0 mmol of aniline (0.27 mL, 1 equiv) was dissolved in 6 mL of ethanol, followed by 3.0 mmol of diethyl acetylenedicarboxylate (0.48 mL, 1 equiv), 3.0 mmol of 3,4-dimethoxybenzaldehyde (0.49 g, 1 equiv) and 2.0 mmol of citric acid monohydrate (0.42 g) were added to the mixture and heated at 60 °C for 5.0 h until the appearance of a solid dispersed in the medium. Once the reaction was completed, the solid obtained was filtered and recrystallized from hot ethanol.

#### 4.6 Enamine Byproduct (Compound **22**)

In a reaction tube, 3.0 mmol of diethyl acetylenedicarboxylate (0.48 mL, 1 equiv) and 3.0 mmol of aniline (0.27 mL, 1 equiv) were mixed. The mixture was stirred at 60 °C for 30 min. Reaction progress was monitored by Thin Layer Chromatography (TLC), yielding a crude mixture as a dark liquid. The product was purified by column chromatography using silica gel as the stationary phase and eluting with hexane/ethyl acetate (4:1). The enamine was isolated as a yellow oily liquid.

All compounds synthesized were characterized by means of 1H and 13C Nuclear Magnetic Resonance and two-dimensional experiments (COSY, HSQC, and HMBC). Also, electron-impact mass spectra were recorded.

##### 4.7 DART-MS Analytical Conditions Applied to the Reaction Mechanism

The instrument used in this work is a JMS-T100LC time-of-flight mass spectrometer equipped with a DART ion source. The ion source was operated in the positive ion mode with helium (He) as the reagent gas at a temperature of 350 °C.

The synthetic procedure for compound 1 was carried out under solvent-free conditions and acid catalyst in ethanol. At specific time intervals (0, 10, and 40 min), aliquots of the reaction mixture were collected to monitor the transformation’s progress. These samples were immediately analyzed using DART-MS.

##### 4.8 Computational Methods

Geometry optimization and partial atomic charge analysis were performed using the Gaussian 16 software package. All calculations were carried out at the M06–2X-D3/def2-TZVP level of theory at 298.15 K. Optimized geometries were confirmed as true minima by frequency analysis (no imaginary frequencies). Partial atomic charges were obtained from the electrostatic potential (ESP) and analyzed to evaluate charge distribution within the molecule, with particular attention to the β-carbon of the enol moiety in diethyl oxaloacetate. The resulting electronic data were used to rationalize the nucleophilic character of this center and to support the proposed reaction mechanism. Detailed numerical values are provided in Supporting Information.

##### 4.9 Experimental Data

**1**. Yield (0.147 g, 15%, solvent-free) (0.523 g, 54%, aqueous medium with β-cyclodextrin) (0.639 g, 65.87%, acid catalysis in ethanol). White solid. Mp 162 °C. ^1^H NMR (300 MHz, CDCl_3_), δ/ppm: 9.04 (1H, *s*, OH), 7.47 (2H, *dd*, J = 8.5, 1.2 Hz, Ar), 7.29–7.19 (7H, *m*, Ar), 7.09 (1H, *tt*, J = 7.4, 1.1 Hz, Ar), 5.73 (1H, *s*, CH), 4.18 (2H, *q*, J = 7.1 Hz, CH_2_), 1.17 (3H, *t*, J = 7.1 Hz, CH_3_). ^13^C NMR (75 MHz, CDCl_3_), δ/ppm: 165.34, 162.94, 156.76, 136.43, 135.23, 129.11, 128.73, 128.66, 127.66, 125.98, 122.43,113.30, 61.69, 61.40, 14.06. HRMS (EI) *m*/*z* calcd for C_19_H_17_NO_4_: 323.1157, found 323.1156.

**2**. Yield (0.578 g, 57%). Light yellow solid. Mp 150 °C. ^1^H NMR (300 MHz, CDCl_3_), δ/ppm: 9.10 (1H, *s*, OH), 7.43–7.38 (2H, *m*, Ar), 7.26–7.18 (5H, *m*, Ar), 6.95 (2H, *t*, J = 8.5 Hz, Ar), 5.66 (1H, *s*, CH), 4.18 (2H, *q*, J = 7.1 Hz, CH_2_), 1.17 (3H, *t*, J = 7.1 Hz, CH_3_). ^13^C NMR (75 MHz, CDCl_3_), δ/ppm: 165.32, 162.89, 160.47 (*d*, *J* = 246.40 Hz), 156.83, 134.97, 132.46, 132.42, 128.83, 127.65, 124.47 (*d*, *J* = 8.28 Hz), 116.01 (*d, J* = 22.7 Hz), 113.29, 62.07, 61.45, 14.05. HRMS (EI) *m*/*z* calcd for C_19_H_16_FNO_4_: 341.1063, found: 341.1070.

**3**. Yield 0.544 g (50%). White solid. Mp 140 °C. ^1^H NMR (300 MHz, CDCl_3_), δ/ppm: 9.05 (1H, *s*, OH), 7.44 (2H, *d*, J = 8.9 Hz, Ar), 7.29–7.19 (7H, *m*, Ar), 5.69 (1H, *s*, CH), 4.19 (2H, *q*, J = 7.1 Hz, CH_2_), 1.17 (3H, *t*, J = 7.1 Hz, CH_3_). ^13^C NMR (75 MHz, CDCl_3_), δ/ppm: 165.28, 162.90, 156.63, 135.07, 134.92, 131.26, 129.24, 128.88, 127.59, 123.33, 113.41, 61.61, 61.50, 14.05. HRMS (EI) *m*/*z* calcd for C_19_H_16_ClNO_4_: 357.0768, found: 357.0753.

**4**. Yield 0.753 g (62%). White solid. Mp 161 °C. ^1^H NMR (300 MHz, CDCl_3_), δ/ppm: 9.07 (1H, *s*, OH), 7.41–7.36 (4H, *m*, Ar), 7.29–7.19 (5H, *m*, Ar), 5.69 (1H, *s*, CH), 4.19 (2H, *q*, J = 7.1 Hz, CH_2_), 1.18 (3H, *t*, J = 7.1 Hz, CH_3_). ^13^C NMR (75 MHz, CDCl_3_), δ/ppm: 165.25, 162.89, 156.56, 135.59, 134.89, 132.18, 128.88, 127.58, 123.55, 119.07, 113.44, 61.51, 31.05, 14.04. HRMS (EI) *m*/*z* calcd for C_19_H_16_BrNO_4_: 401.0263, found: 401.0268.

**5**. Yield 0.669 g (50%). White solid. Mp 185 °C. ^1^H NMR (300 MHz, CDCl_3_), δ/ppm: 9.05 (1H, *s*, OH), 7.59–7.54 (2H, *m*, Ar), 7.30–7.18 (7H, *m*, Ar), 5.68 (1H, *s*, CH), 4.18 (2H, *q*, J = 7.1 Hz, CH_2_), 1.18 (3H, *t*, J = 7.1 Hz, CH_3_). ^13^C NMR (75 MHz, CDCl_3_), δ/ppm: 165.30, 162.85, 156.62, 138.13, 136.34, 134.91, 128.91, 128.89, 127.57, 123.69, 113.45, 90.12, 61.53, 61.37, 14.06. HRMS (EI) *m*/*z* calcd for C_19_H_16_INO_4_: 449.0124, found: 449.0111.

**6**. Yield 0.327 g (30%). Brown solid. Mp 162 °C. ^1^H NMR (400 MHz, CDCl_3_), δ/ppm: 9. Eleven (1H, *s*, OH), 8.14 (2H, *d*, J = 9.3 Hz, Ar), 7.80 (2H, *d*, J = 6.1 Hz, Ar), 7.32–7.24 (5H, *m*, Ar), 5.80 (1H, *s*, CH), 4.22 (2H, *q*, J = 7.1 Hz, CH_2_), 1.21 (3H, *t*, J = 7.1 Hz, CH_3_). ^13^C NMR (100 MHz, CDCl_3_), δ/ppm: 165.09, 163.36, 155.89, 144.31, 142.22, 134.45, 129.19, 129.13, 127.43, 124.82, 120.73, 114.21, 61.80, 61.28, 14.03. HRMS (EI) *m*/*z* calcd for C_19_H_16_N_2_O_6_: 368.1008, found: 368.1017.

**7**. Yield 0.447 g (43%). White solid. Mp 162 °C. ^1^H NMR (300 MHz, CDCl_3_), δ/ppm: 9.03 (1H, *s*, OH), 7.33–7.17 (7H, *m*, Ar) 6.80–6.76 (2H, *m*, Ar), 5.63 (1H, *s*, CH), 4.16 (2H, *q*, J = 7.1 Hz, CH_2_), 3.72 (3H, *s*, CH_3_), 1.16 (3H, *t*, J = 7.1 Hz, CH_3_). ^13^C NMR (75 MHz, CDCl_3_), δ/ppm: 165.38, 162.81, 157.72, 157.09, 135.31, 129.31, 128.71, 128.65, 127.72, 124.52, 114.37, 113.01, 62.26, 61.31, 55.47, 14.06. HRMS (EI) *m*/*z* calcd for C_20_H_19_NO_5_: 353.1263, found: 353.1274.

**8**. Yield 0.745 g (58%). Yellow solid. Mp 194–196 °C. ^1^H NMR (300 MHz, DMSO-*d*_6_), δ/ppm: 11.82 (1H, *s*, OH), 7.87–7.83 (6H, *m*, Ar), 7.58–7.16 (4H, *m*, Ar), 7.34–7.16 (5H, *m*, Ar), 6.18 (1H, *s*, CH), 4.10–3.99 (2H, *m*, CH_2_), 1.10 (3H, *t*, J = 7.1 Hz, CH_3_). ^13^C NMR (75 MHz, DMSO-*d*_6_), δ/ppm: 164.31, 161.86, 152.30, 151.93, 148.55, 139.03, 136.35, 131.37, 129.40, 128.86, 128.17, 128.00, 127.74, 127.66, 127.17, 123.03, 122.41, 122.19, 122.69, 119.68, 112.61, 60.48, 59.75, 30.67, 13.96. HRMS (EI) *m*/*z* calcd for C_25_H_21_N_3_O_4_: 427.1532, found: 427.1548.

**9**. Yield 0.283 g (20%). Light yellow. Mp 172 °C. ^1^H NMR (400 MHz, CDCl_3_), δ/ppm: 9.13 (1H, *s*, OH), 8.08 (2H, *s*, Ar), 7.57 (1H, *s*, Ar), 7.34–7.23 (6H, *m*, Ar), 5.78 (1H, *s*, CH), 4.20 (2H, *q*, J = 7.1 Hz, CH_2_), 1.18 (3H, *t*, J = 7.1 Hz, CH_3_). ^13^C NMR (100 MHz, CDCl_3_), δ/ppm: 165.14, 163.21, 156.08, 138.12, 134.12, 132.42 (*q*, J = 33.69 Hz), 129.33, 129.22, 127.49, 124.37, 121.65, 120.93, 120.88, 118.77, 114.03, 61.78, 61.41, 14.00. HRMS (EI) *m*/*z* calcd for C_21_H_15_F_6_NO_4_: 459.0905, found: 459.0915.

**10**. Yield 0.474 g (38%). White solid. Mp 179 °C. ^1^H NMR (400 MHz, CDCl_3_), δ/ppm: 9.19 (1H, *s*, OH), 7.25–7.11 (8H, *m*, Ar), 5.72 (1H, *s*, CH), 4.16 (2H, *q*, J = 7.1 Hz, CH_2_), 1.12 (3H, *t*, J = 7.1 Hz, CH_3_). ^13^C NMR (100 MHz, CDCl_3_), δ/ppm: 165.47, 162.83, 158.65, 157.03, 156.95 (*d*, J = 122.11 Hz), 134.44, 129.68, 129.06, 128.83, 128.09, 128.04, 127.66, 122.68 (*d, J* = 11.53 Hz), 121.37 (*d, J* = 9.28 Hz), 120.54, 114.04, 62.54, 61.48, 13.99. HRMS (EI) *m*/*z* calcd for C_19_H_15_FBrNO_4_: 419.0168, found: 419.0154.

**11**. Yield 0.67 g (53%). White solid. Mp 151 °C. ^1^H NMR (400 MHz, CDCl_3_), δ/ppm: 9.11 (1H, *s*, OH), 7.85 (1H, *d*, J = 2.7 Hz, Ar), 7.71 (1H, *dd*, J = 8.8, 2.7 Hz, Ar), 7.37 (1H, *d*, J = 8.8 Hz, Ar), 7.31–7.25 (3H, *m*, Ar), 7.23–7.20 (2H, *m*, Ar), 5.71 (1H, *s*, CH), 4.18 (2H, *q*, J = 7.1 Hz, CH_2_), 1.16 (3H, *t*, J = 7.1 Hz, CH_3_). ^13^C NMR (100 MHz, CDCl_3_), δ/ppm: 165.19, 163.03, 156.30, 135.46, 134.41, 132.15, 129.19, 129.12, 128.71, 127.51, 125.53, 122.48 (*q*, J = 273.63 Hz) 120.44, 113.77, 61.68, 61.46, 14.02. HRMS (EI) *m*/*z* calcd for C_20_H_15_ClF_3_NO_4_: 425.0642, found: 425.0642.

**12**. Yield 0.47 g (40%). Light brown solid. Mp 166–168 °C. ^1^H NMR (400 MHz, CDCl_3_), δ/ppm: 9.09 (1H, *s*, OH), 7.74 (1H, *d*, J = 2.4 Hz, Ar), 7.36–7.26 (1H, *m*, Ar), 7.22–7.20 (1H, *m*, Ar), 5.67 (1H, *s*, CH), 4.19 (2H, *q*, J = 7.1 Hz, CH_2_), 1.18 (3H, *t*, J = 7.1 Hz, CH_3_). ^13^C NMR (100 MHz, CDCl_3_) δ/ppm: 165.19, 162.92, 156.30, 135.96, 134.57, 133.06, 130.63, 129.40, 129.02, 127.51, 123.57, 120.87, 113.67, 61.61, 61.46, 14.03. HRMS (EI) *m*/*z* calcd for C_19_H_15_Cl_2_NO_4_: 391.0378, found: 391.0388.

**13**. Yield 0.28 g (26%). Light yellow solid. Mp 183 °C. ^1^H NMR (400 MHz, CDCl_3_), δ/ppm: 8.90 (1H, *s*, OH), 7.55 (2H, *d*, J = 7.6 Hz, Ar), 7.29 (2H, *t*, J = 7.6 Hz, Ar), 7.09 (1H, *t*, J = 7.4 Hz, Ar), 6.78 (1H, *d*, J = 1.8 Hz, Ar), 6.61 (1H, *td*, J = 8.2, 1.3 Hz, Ar), 6.60 (1H, *t*, J = 8.1 Hz, Ar), 5.94 (1H, *s*, CH), 4.12–3.97 (2H, *m*, CH_2_), 3.66 (3H, *s*, CH_3_), 1.11 (3H, *t*, J = 7.1 Hz, CH_3_). ^13^C NMR (100 MHz, CDCl_3_), δ/ppm: 165.44, 162.91, 156.59, 146.90, 145.91, 136.43, 129.11, 126.70, 126.07, 122.60, 121.88, 114.36, 113.34, 108.77, 61.69, 61.44, 56.13, 14.15. HRMS (EI) *m*/*z* calcd for C_20_H_19_NO_6_: 369.1212, found: 369.1194.

**14**. Yield 0.55 g (50%). Light yellow solid. Mp 185 °C. ^1^H NMR (400 MHz, CDCl_3_), δ/ppm: 9.15 (1H, *s*, OH), 7.94 (1H, *dd*, J = 8.2, 1.2 Hz, Ar), 7.73 (1H, *d*, J = 7.8 Hz, Ar), 7.47 (1H, *td*, J = 7.5, 1.2 Hz, Ar), 7.38 (1H, *td*, J = 8.2, 1.7 Hz, Ar), 7.31 (1H, *td*, J = 7.5, 1.7 Hz, Ar), 7.14 (1H, *td*, J = 8.2, 1.2 Hz, Ar), 7.12 (1H, *s*, CH), 7.12 (1H, *tt*, J = 8.0, 1.0 Hz, Ar), 4.24–4.11 (2H, *m*, CH_2_), 1.15 (3H, *t*, J = 7.1 Hz, CH_3_). ^13^C NMR (100 MHz, CDCl_3_) δ/ppm: 164.97, 162.86, 157.45, 150.17, 136.18, 133.96, 130.86, 129.48, 129.43, 127.23, 126.12, 125.32, 121.65, 113.16, 61.95, 54.34, 14.01. HRMS (EI) *m*/*z* calcd for C_19_H_16_N_2_O_6_: 368.1008, found: 368.1010.

**15**. Yield 0.49 g (48%). White solid. Mp 187 °C. ^1^H NMR (300 MHz, CDCl_3_), δ/ppm: 9.06 (1H, *s*, OH), 7.46–7.42 (2H, *m*, Ar), 7.30–7.25 (2H, *m*, Ar), 7.22–7.17 (2H, *m*, Ar), 7.12 (1H, *tt*, J = 7.4, 1.2 Hz, Ar), 6.97–6.91 (2H, *m*, Ar), 5.72 (1H, *s*, CH), 4.24–4.16 (2H, *m*, CH_2_), 1.19 (3H, *t*, J = 7.1 Hz, CH_3_). ^13^C NMR (75 MHz, CDCl_3_), δ/ppm: 165.12, 162.82, 162.71 (*d*, J = 247.7 Hz), 156.65, 136.15, 131.00 (*d*, J = 3.2 Hz), 129.42, 129.31, 129.18, 126.17, 122.54, 115.81 (*d*, J = 21.9 Hz), 113.09, 61.47, 61.00, 14.10. HRMS (EI) *m*/*z* calcd for C_19_H_16_FNO_4_: 341.1063, found: 341.1055.

**16**. Yield 0.49 g (45%). White solid. Mp 190 °C. ^1^H NMR (300 MHz, CDCl_3_), δ/ppm: 9.07 (1H, *s*, OH), 7.46–7.42 (H, *m, Ar*), 7.30–7.21 (4H, *m*, Ar), 7.18–7.10 (3H, *m*, Ar), 5.75 (1H, *s*, CH), 4.20 (2H, *q*, J = 7.1 Hz, CH_2_), 1.20 (3H, *t*, J = 7.1 Hz, CH_3_). ^13^C NMR (75 MHz, CDCl_3_), δ/ppm: 165.05, 162.80, 156.73, 136.12, 134.49, 133.90, 129.23, 129.04, 129.01, 126.21, 122.43, 112.92, 61.55, 60.99, 14.12. HRMS (EI) *m*/*z* calcd for C_19_H_16_ClNO_4_: 357.0768, found: 357.0763.

**17**. Yield 0.78 g (65%). Light pink solid. Mp 197 °C. ^1^H NMR (300 MHz, CDCl_3_), δ/ppm: 9.26 (1H, *s*, OH), 7.55–7.50 (3H, *m*, Ar), 7.17 (1H, *td*, J = 7.4, 1.2 Hz, Ar), 7.10 (1H, *tt*, J = 7.4, 1.2 Hz, Ar), 7.06 (1H, *dd*, J = 7.3, 1.7 Hz, Ar), 6.95 (1H, *dd*, J = 7.8, 1.7 Hz, Ar), 6.41 (1H, *s*, CH), 4.25–4.11 (2H, *m*, CH_2_), 1.18 (3H, *t*, J = 7.1 Hz, CH_3_). ^13^C NMR (75 MHz, CDCl_3_), δ/ppm: 165.51, 162.82, 157.63, 136.16, 134.46, 133.13, 130.08, 129.18, 128.27, 127.28, 126.05, 125.70, 122.11, 113.28, 61.53, 59.50, 13.99. HRMS (EI) *m*/*z* calcd for C_19_H_16_BrNO_4_: 401.0263, found: 401.0252.

**18**. Yield 0.55 g (46%). White solid. Mp 180 °C. ^1^H NMR (400 MHz, CDCl_3_), δ/ppm: 9.04 (1H, *s*, OH), 7.45 (2H, *d*, J = 8.0 Hz, Ar), 7.39 (2H, *d*, J = 8.4 Hz, Ar), 7.29 (2H, *t*, J = 7.6 Hz, Ar), 7.15–7.10 (3H, *m*, Ar), 5.70 (1H, *s*, CH), 4.21 (2H, *q*, J = 7.1 Hz, CH_2_), 1.22 (3H, *t*, J = 7.1 Hz, CH_3_). ^13^C NMR (100 MHz, CDCl_3_), δ/ppm: 165.04, 162.78, 156.74, 136.12, 134.46, 131.98, 129.32, 129.24, 126.20, 122.64, 122.39, 112.86, 61.56, 61.03, 14.12. HRMS (EI) *m*/*z* calcd for C_19_H_16_BrNO_4_: 401.0263, found: 401.0262.

**19**. Yield 0.50 g (39%). White solid. Mp 170 °C. ^1^H NMR (400 MHz, CDCl_3_), δ/ppm: 9.00 (1H, *s*, OH), 7.48–7.45 (2H, *m*, Ar), 7.38–7.25 (7H, *m*, Ar), 7.14–7.08 (3H, *m*, Ar), 6.86–6.83 (2H, *m*, Ar), 5.68 (1H, *s*, CH), 4.97 (2H, *s*, CH_2_), 4.19 (2H, *q*, J = 7.1 Hz, CH_2_), 1.18 (3H, *t*, J = 7.1 Hz, CH_3_). ^13^C NMR (100 MHz, CDCl_3_), δ/ppm: 165.31, 162.93, 158.95, 156.47, 136.77, 136.42, 129.09, 128.84, 128.70, 128.18, 127.66, 127.21, 125.94, 122.53, 70.12, 61.36, 61.21, 14.10. HRMS (EI) *m*/*z* calcd for C_26_H_23_N_1_O_5_: 429.1576, found: 429.1593.

**20**. Yield 0.89 g (84%). Light yellow solid. Mp 163 °C. ^1^H NMR (300 MHz, CDCl_3_), δ/ppm: 9.06 (1H, *s*, OH), 7.55–7.53 (2H, m, Ar), 7.27–7.22 (3H, *m*, Ar), 7.17 (1H, *td*, J = 7.3, 1.7 Hz, Ar), 7.06 (1H, *tt*, J = 7.4, 1.1 Hz, Ar), 6.82 (1H, *d*, J = 7.6 Hz, *Ar*), 6.80 (1H, *dd*, J = 7.7, 1.0 Hz, Ar), 4.14 (2H, *q*, J = 7.1 Hz, CH_2_), 3.88 (3H, *s*, OCH_3_), 1.12 (3H, *t*, J = 7.1 Hz, CH_3_). ^13^C NMR (75 MHz, CDCl_3_), δ/ppm: 165.58, 163.11, 157.89, 157.28, 136.70, 129.75, 128.91, 125.58, 123.08, 121.81, 121.02, 111.20, 61.13, 55.84, 13.99. HRMS (EI) *m*/*z* calcd for C_20_H_19_NO_5_: 353.1263, found: 353.1251.

**21**. Yield 0.77 g (67%). Light yellow solid. Mp 163 °C. ^1^H NMR (300 MHz, CDCl_3_), δ/ppm: 9.06 (1H, *s*, OH), 7.46 (2H, *d*, J = 8.3 Hz, Ar), 7.28 (2H, *t*, J = 7.6 Hz, Ar), 7.11 (1H, *tt*, J = 7.4, 0.9 Hz, Ar), 6.84 (1H, *dd*, J = 8.3, 2.0 Hz, Ar), 6.73 (1H, d, J = 8.3 Hz, Ar), 6.07 (1H, *d*, J = 2.0 Hz, Ar), 5.68 (1H, *s*, CH), 4.26–4.16 (2H, *m*, CH_2_), 3.81 (3H, *s*, OCH_3_), 3.78 (3H, *s*, OCH_3_), 1.22 (3H, *t*, J = 7.1 Hz, CH_3_). ^13^C NMR (75 MHz, CDCl_3_), δ/ppm: 165.37, 162.89, 156.56, 149.23, 149.20, 136.41, 129.10, 127.30, 126.07, 122.63, 120.87, 113.25, 110.99, 109.76, 61.63, 61.41, 56.07, 55.91, 14.17. HRMS (EI) *m*/*z* calcd for C_21_H_21_NO_6_: 383.1369, found: 383.1384.

#### 4.5.21.4 Enamine Byproduct **22**

Quantitative yield. Yellow liquid. ^1^H NMR (300 MHz, CDCl_3_), δ/ppm: 9.67 (1H, *s*, OH), 7.29–7.24 (2H, *m*, Ar), 7.08 (1H, *tt*, J = 7.4, 1.2 Hz, Ar), 6.92 (2H, *d*, J = 7.5 Hz, Ar), 5.38 (1H, *s*, CH), 4.19 (2H, *q*, J = 7.1 Hz, CH_2_), 4.15 (2H, *q*, J = 7.13 Hz, CH_2_), 1.30 (3H, *t*, J = 7.1 Hz, CH_3_), 1.09 (3H, *t*, J = 7.1 Hz, CH_3_). ^13^C NMR (75 MHz, CDCl_3_), δ/ppm: 169.69, 164.52, 148.55, 140.49, 129.17, 124.35, 121.14, 93.88, 62.16, 60.06, 14.46, 13.74.

The final atomic coordinates and crystallographic data for structures **2**, **9**, **12**, and **20** have been deposited in the joint CCDC/FIZ Karlsruhe deposition service. They are available upon request, quoting the deposition numbers CCDC 2479229, 2479230, 2479231, and 2479232

## Supplementary Material



## Notes

Spectroscopic data for compounds **1–21** (^1^H, ^13^C NMR, and EI-MS), crystallographic details and refinement parameters for single-crystal X-ray diffraction analyses of compounds **2**, **9**, **12**, and **20**, experimental procedures and chromatographic data from the water-influence studies, HPLC and NMR experiments confirming enamine formation as a reaction byproduct, and a computational partial charge analysis of diethyl oxaloacetate performed at the M06–2X-D3/def2-TZVP level of theory (PDF)
