# Supplementary material for: Elucidating the Multicomponent Reaction Pathway of 2‑Pyrrolidone Synthesis
Source: ACS Omega. 2025 Dec 26;11(2):2687–702. doi: 10.1021/acsomega.5c08141 (PMC12824766; doi:10.1021/acsomega.5c08141)
Supplement: Supplementary file 1 [file ao5c08141_si_001.pdf]

Supporting Information

Elucidating the Multicomponent Reaction Pathway of 2-Pyrrolidone Synthesis

Alexander Dueñas-Deyá[1], Reyna Evelyn Cordero-Rivera[1], Mariano Martínez-Vázquez[1*]

[1]Instituto de Química, Circuito Exterior s/n, Circuito de la Investigación Científica, Universidad Nacional Autónoma de México, 04510 Coyoacán, Ciudad de México.

*Corresponding author: marvaz@unam.mx

Spectroscopic information of compounds 1-21

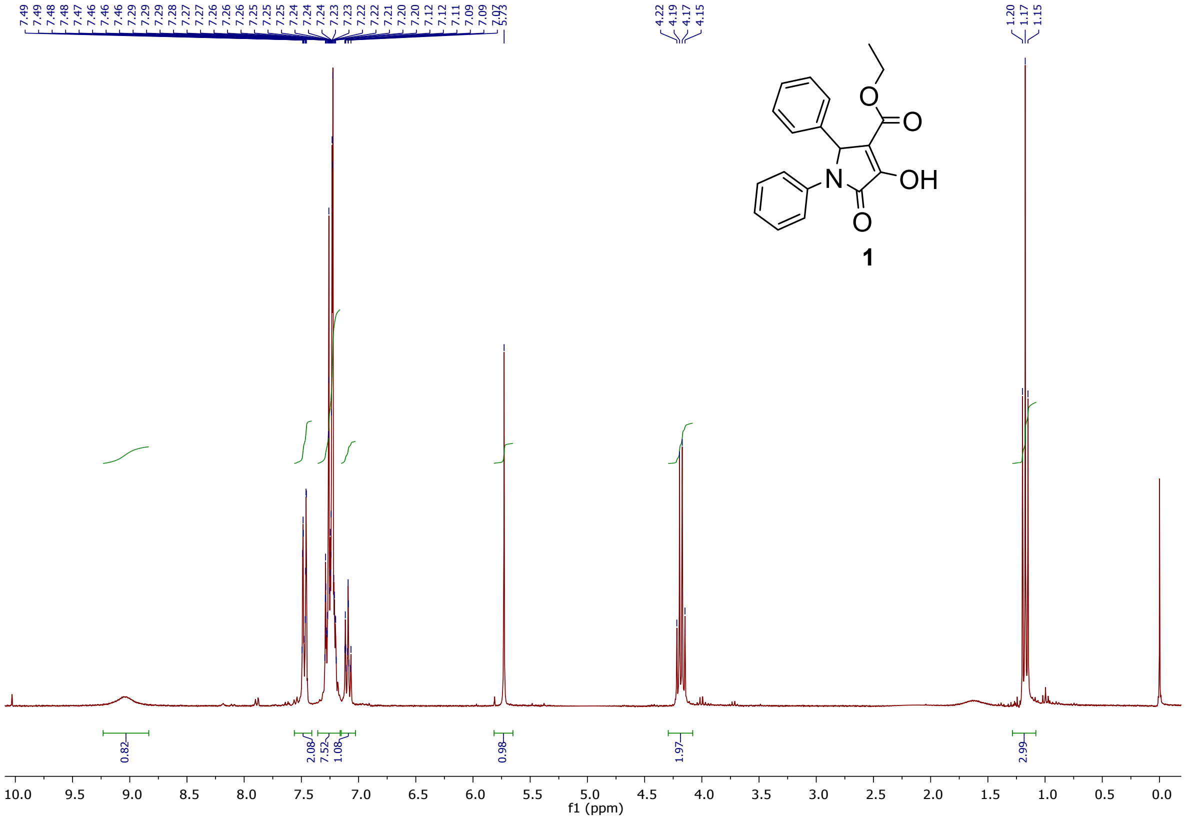

Figure S1. ^{1}H NMR spectrum of compound **1** at 300 MHz in $CDCl_3$.

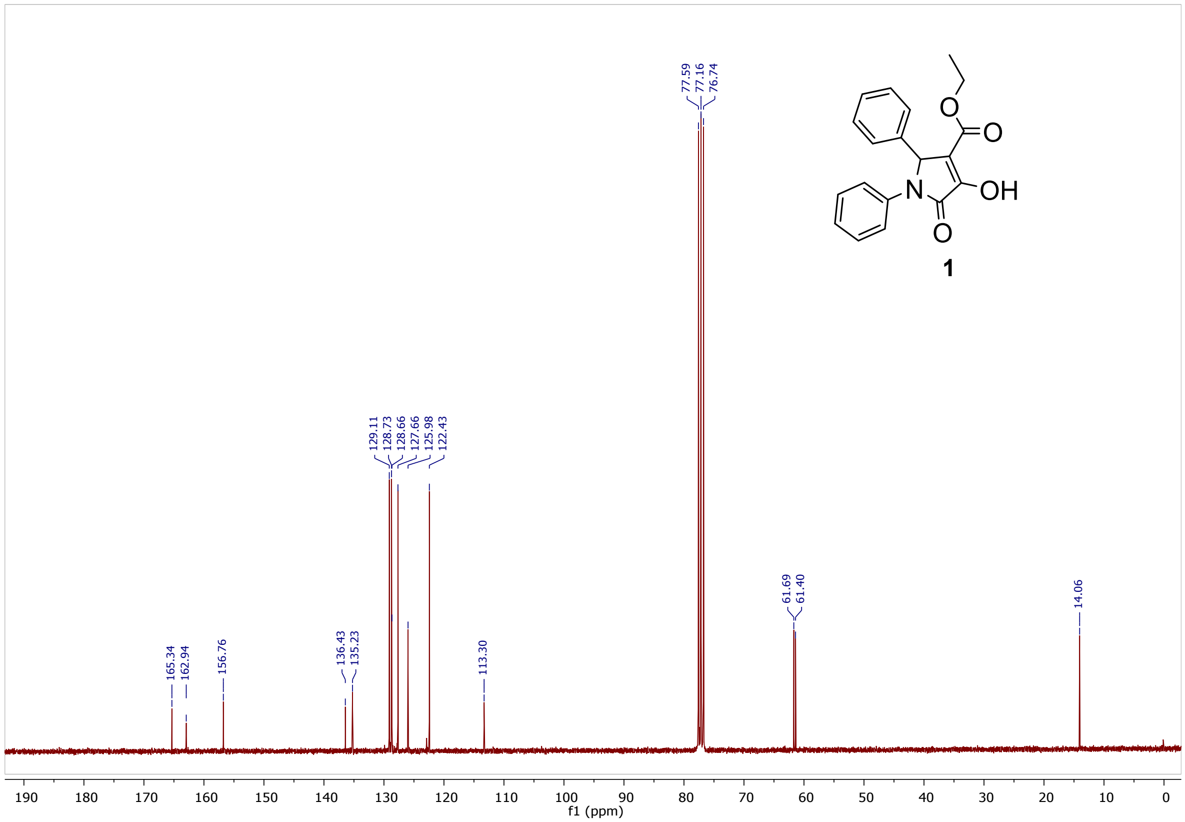

Figure S2. ^{13}C NMR spectrum of compound **1** at 75 MHz in $CDCl_3$.

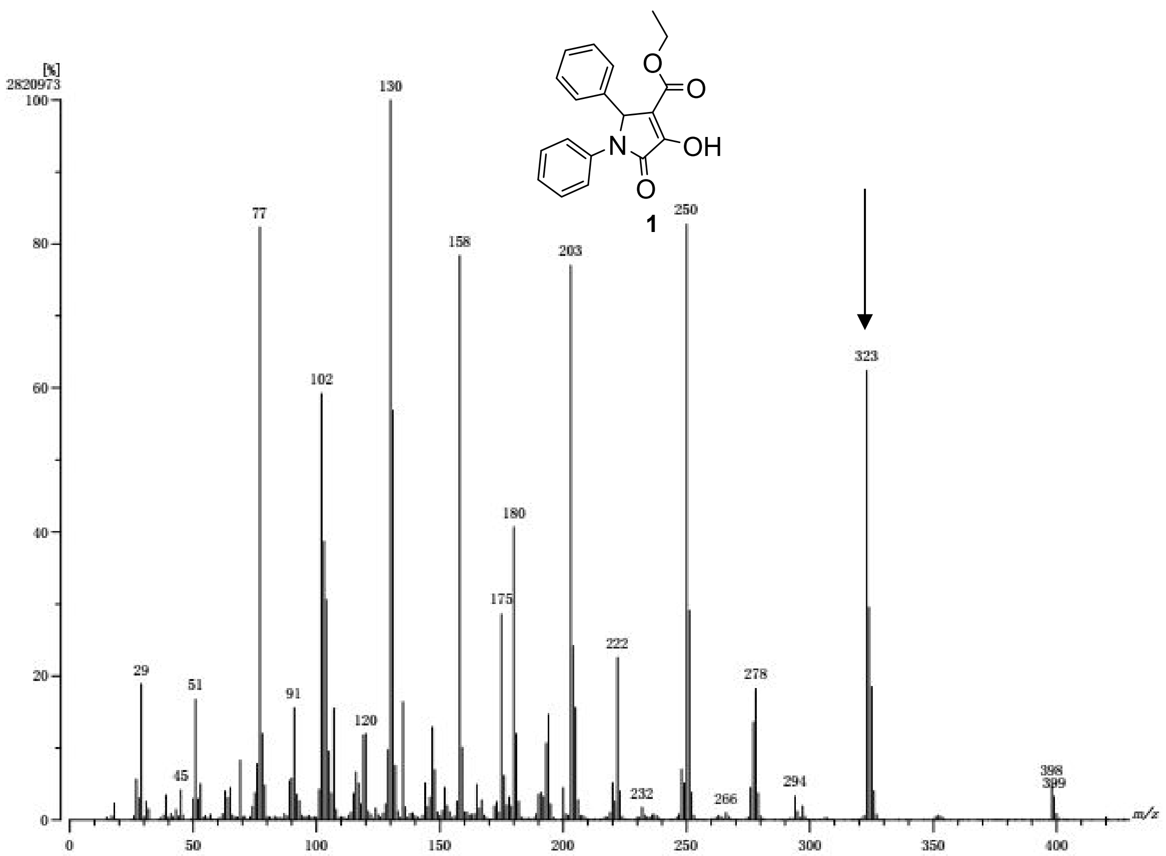

Figure S3. Electron impact mass spectra of compound **1**.

Table S1. Exact masses of main fragments of compound **1** determined by Electron Impact Mass Spectrometry.

Molecular formula	Estimated m/z	Observed m/z	Error (ppm)
$C_{19}H_{17}NO_4$	323.1158	323.1170	3.8
$C_{16}H_{12}NO_2$	250.0839	250.0868	-11.6
$C_{12}H_{11}O_3$	203.0708	203.0695	-6.5
C_9H_6O	130.0419	130.0409	-7.4

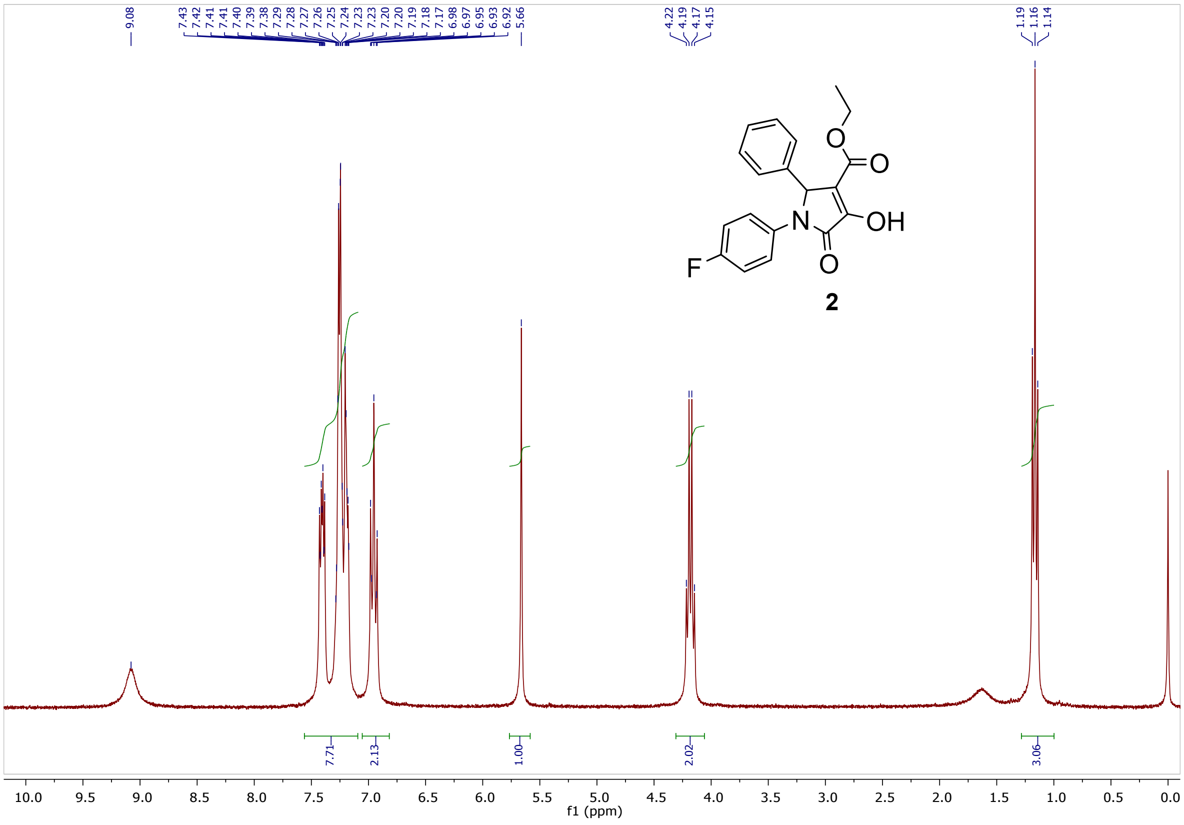

Figure S4. ^{1}H NMR spectrum of compound **2** at 300 MHz in $CDCl_3$.

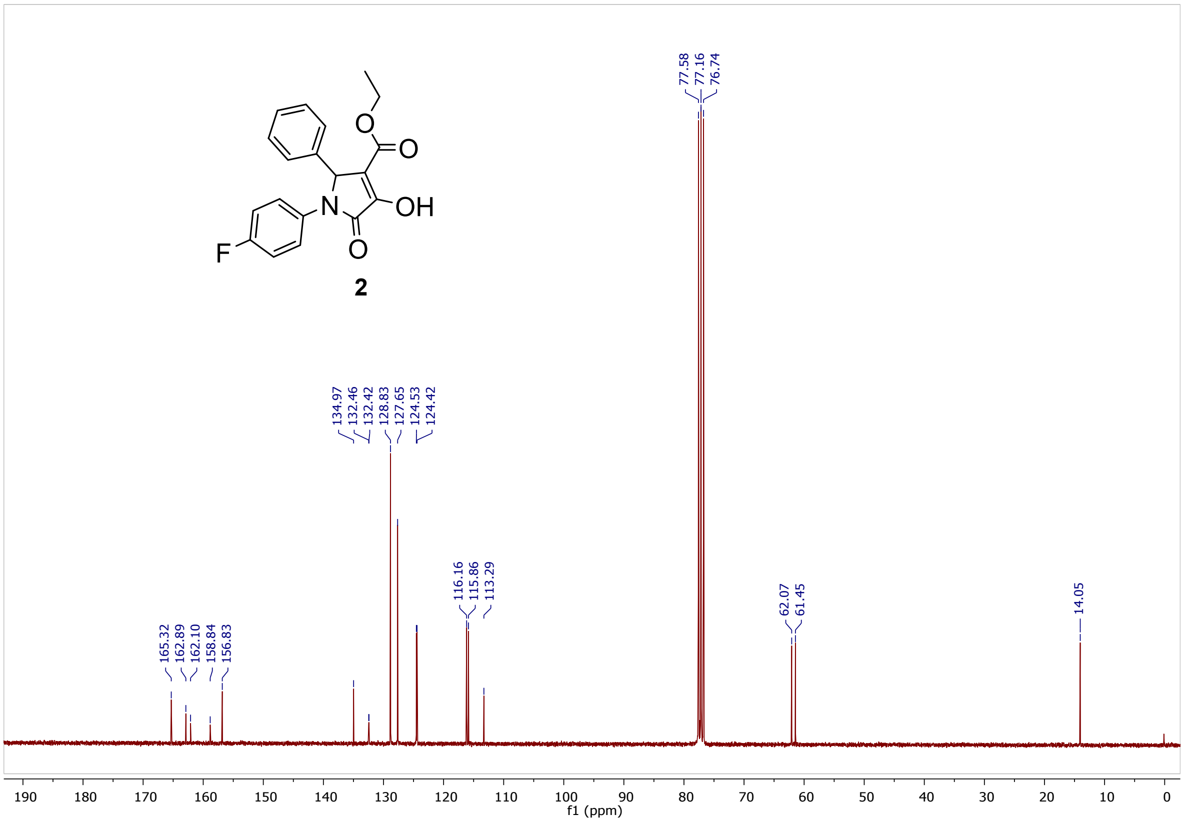

Figure S5. ^{13}C NMR spectrum of compound **2** at 75 MHz in $CDCl_3$.

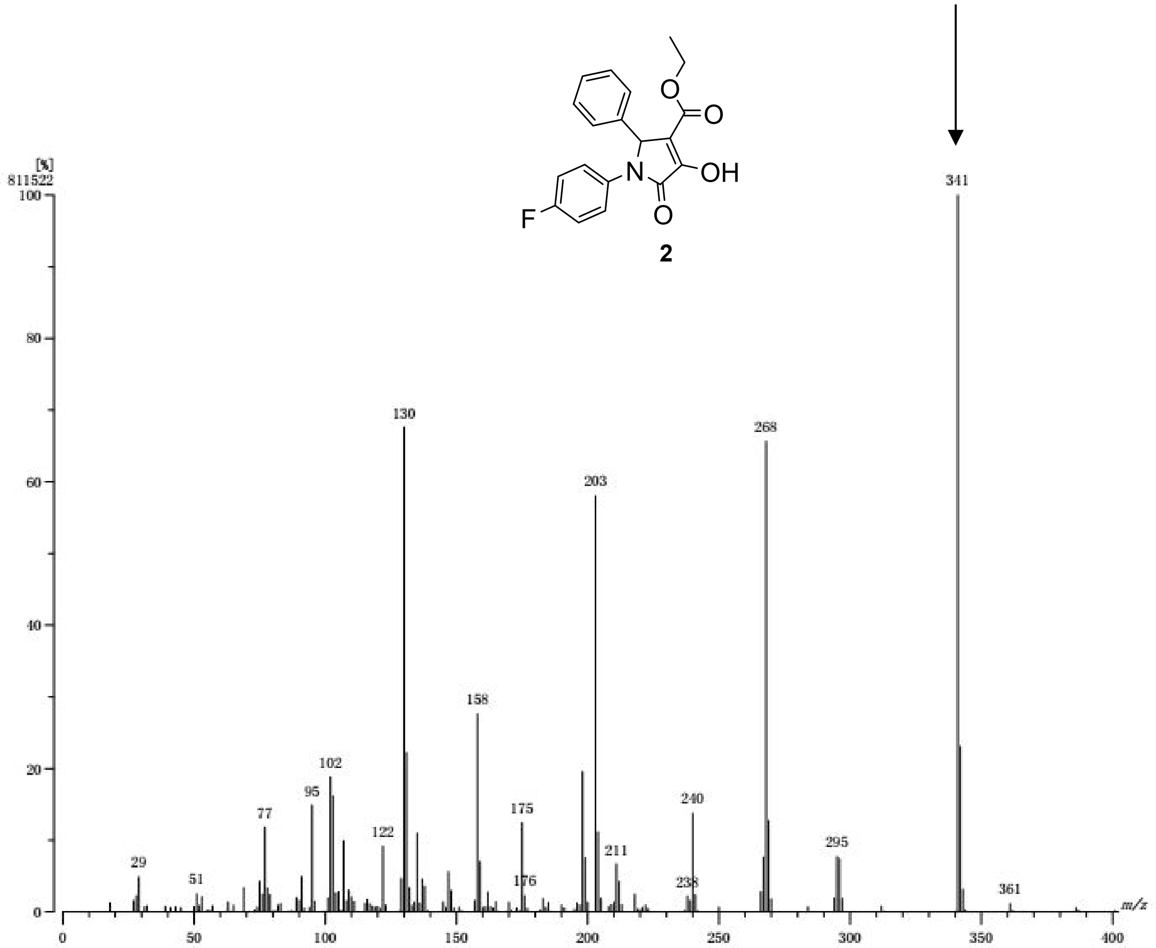

Figure S6. Electron impact mass spectra of compound **2**.

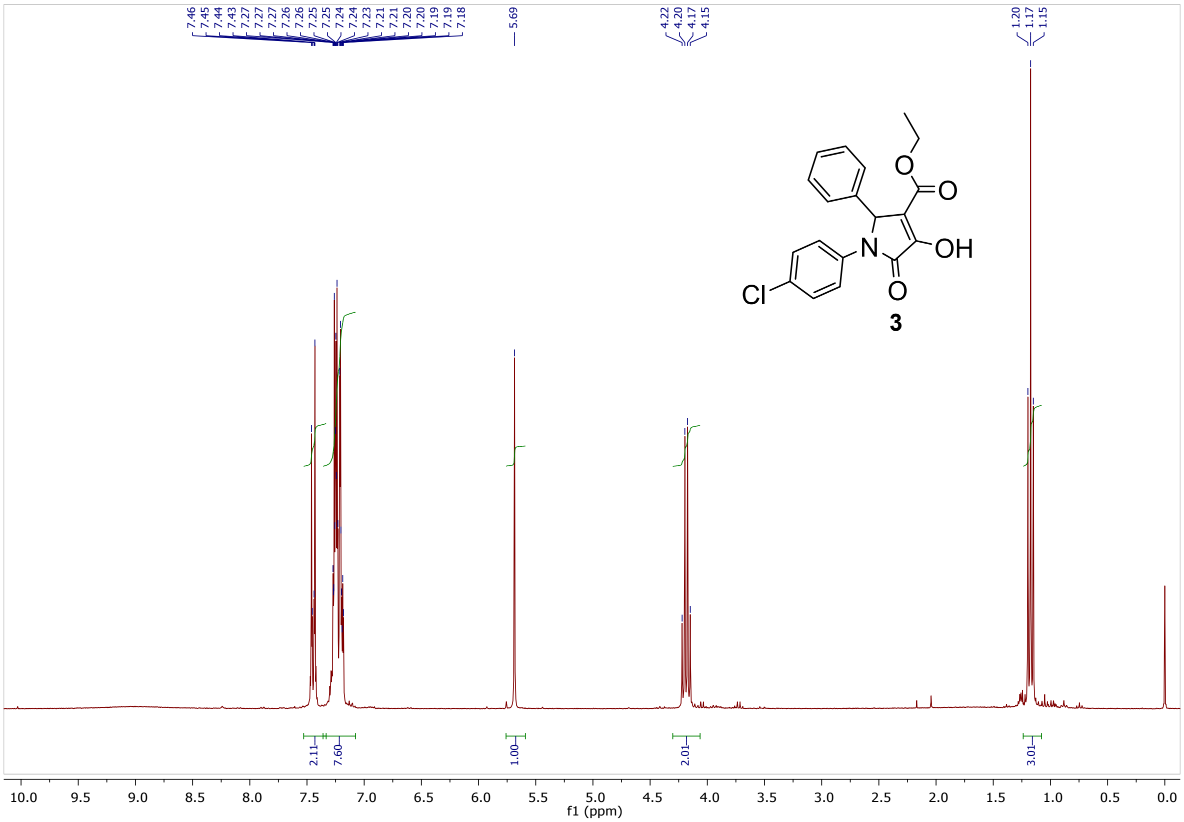

Figure S7. ^{1}H NMR spectrum of compound **3** at 300 MHz in $CDCl_3$.

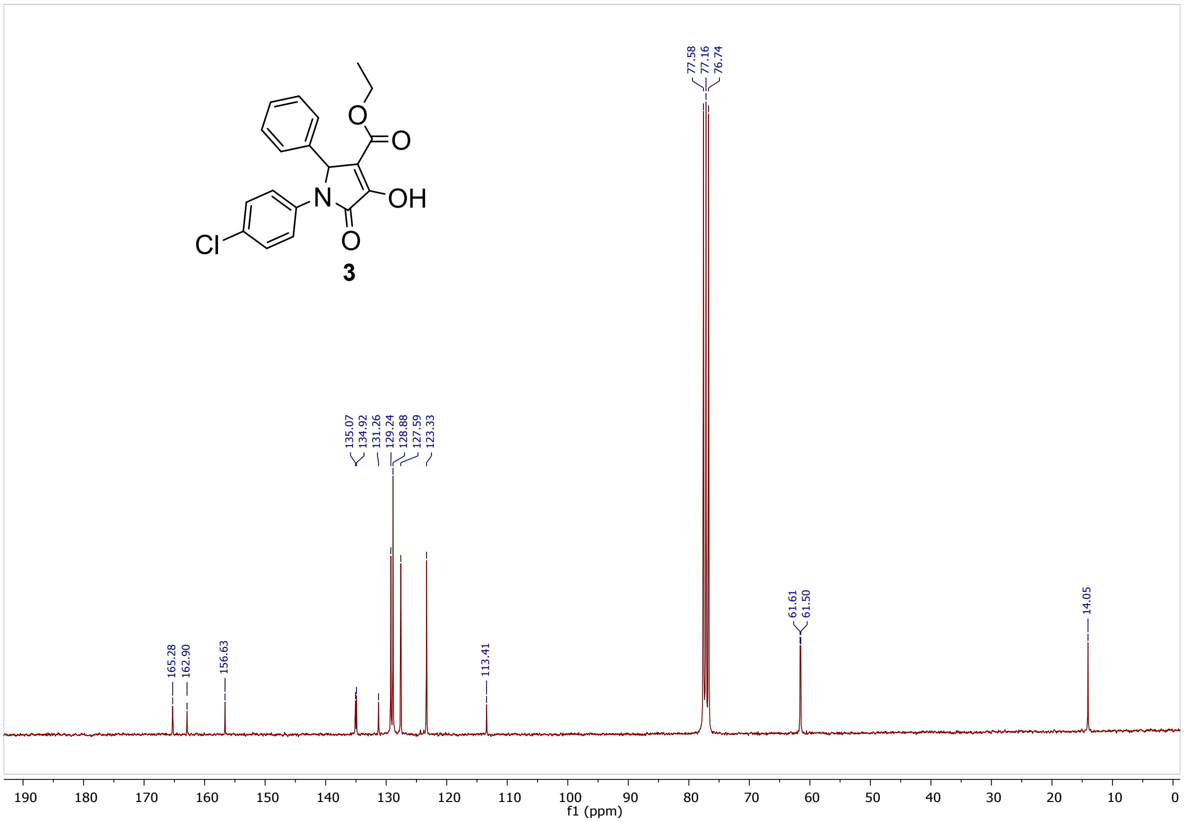

Figure S8. ^{13}C NMR spectrum of compound **3** at 75 MHz in $CDCl_3$.

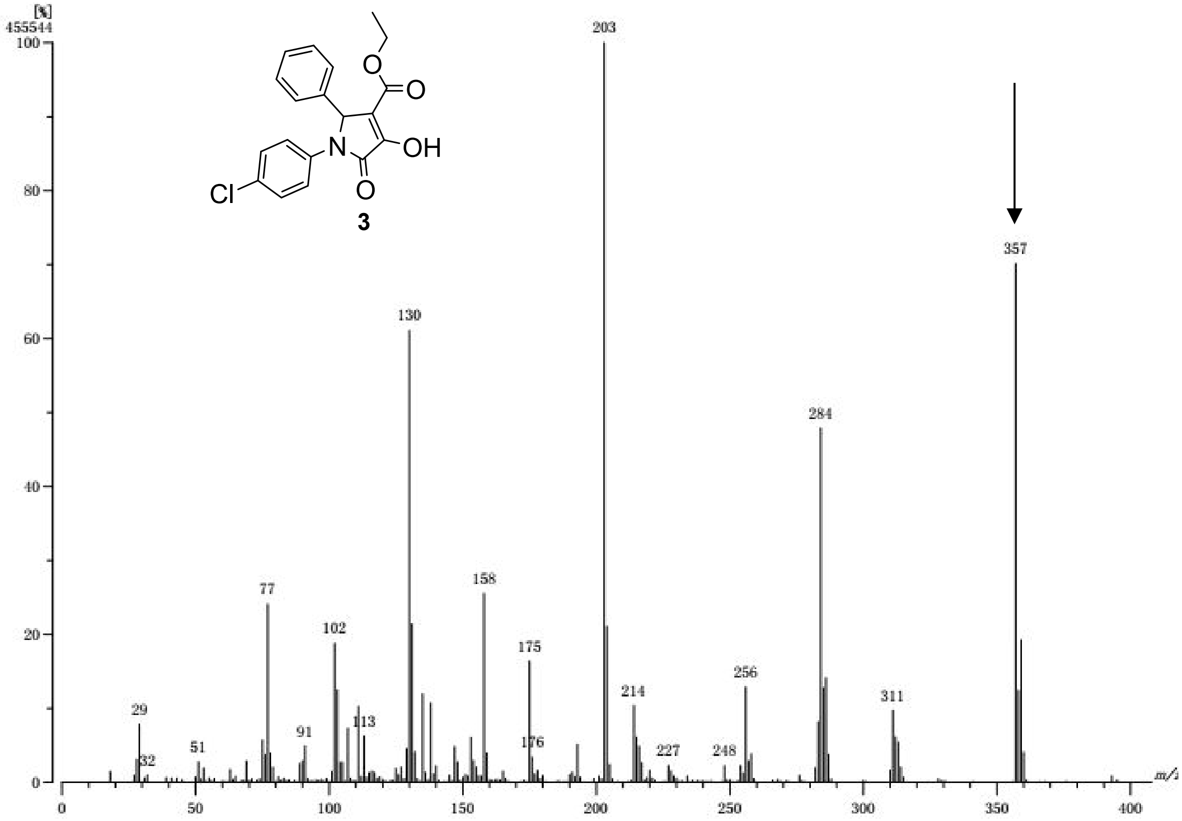

Figure S9. Electron impact mass spectra of compound **3**

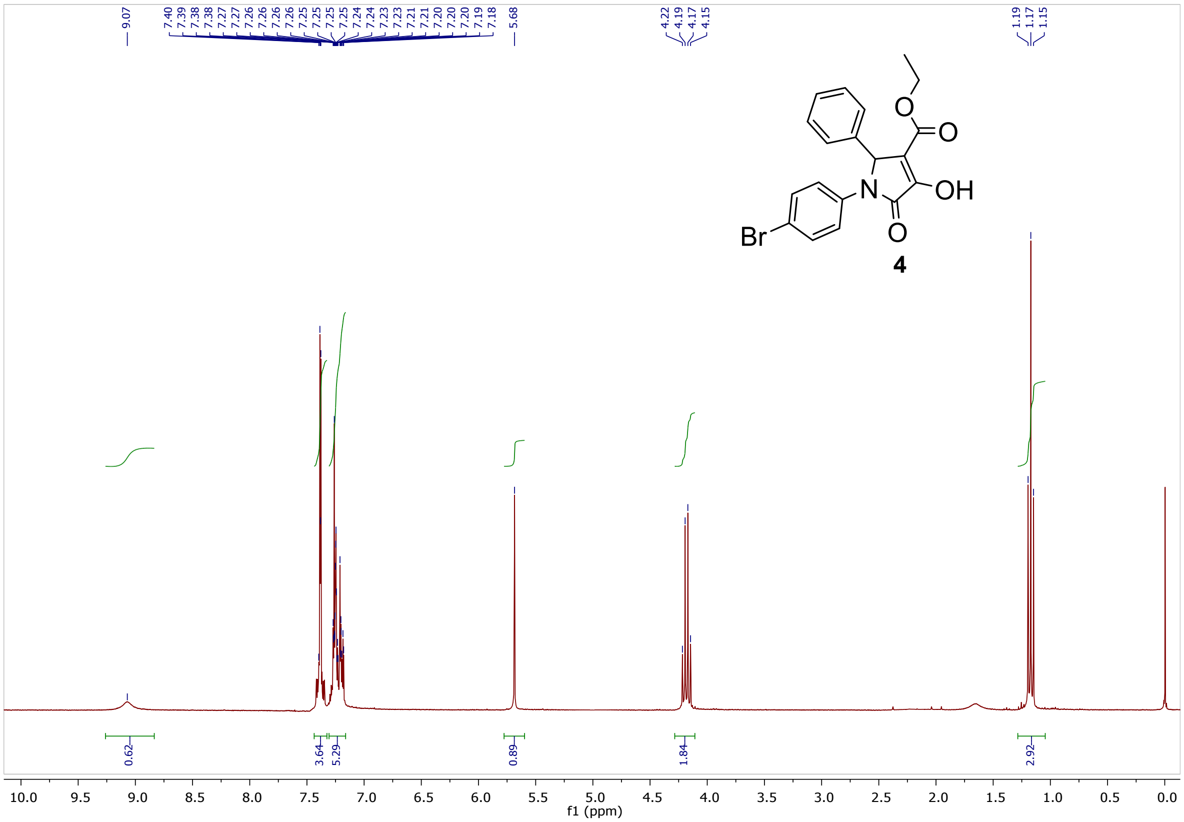

Figure S10. ^1H NMR spectrum of compound **4** at 300 MHz in $CDCl_3$.

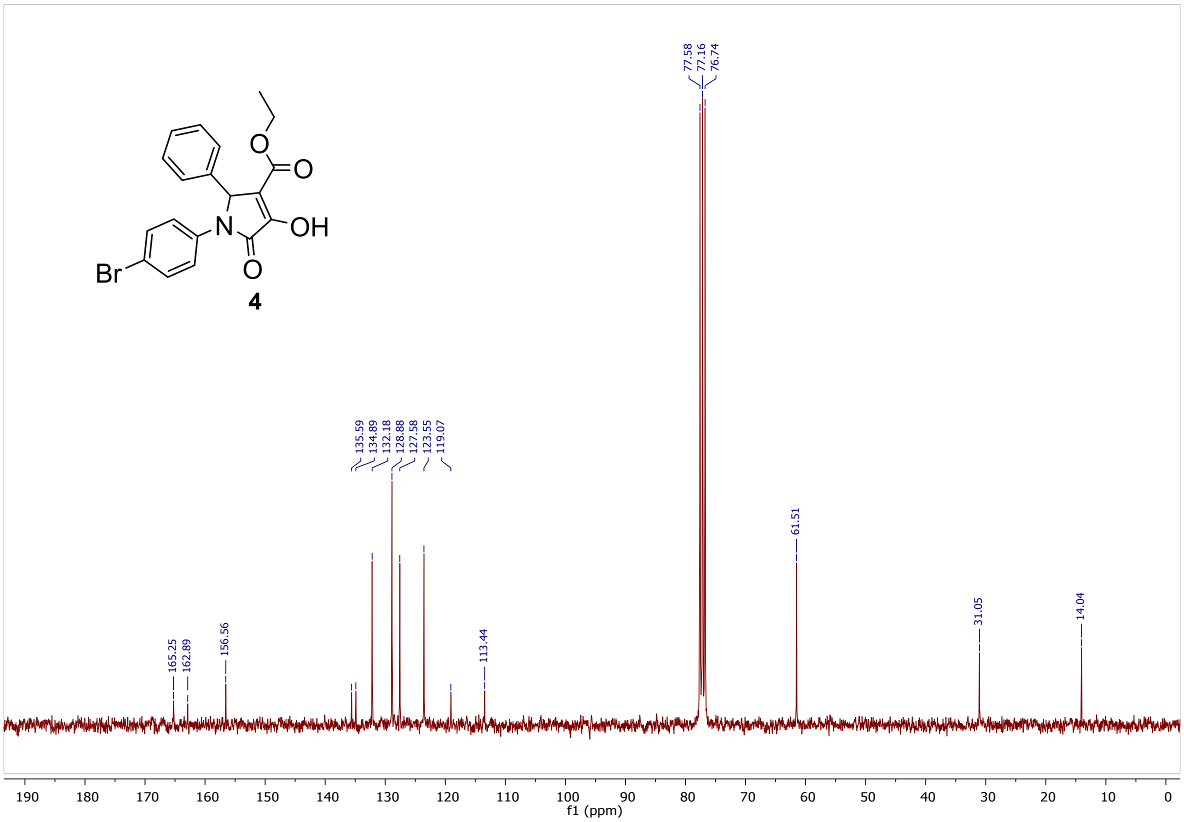

Figure S11. ^{13}C NMR spectrum of compound **4** at 75 MHz in $CDCl_3$.

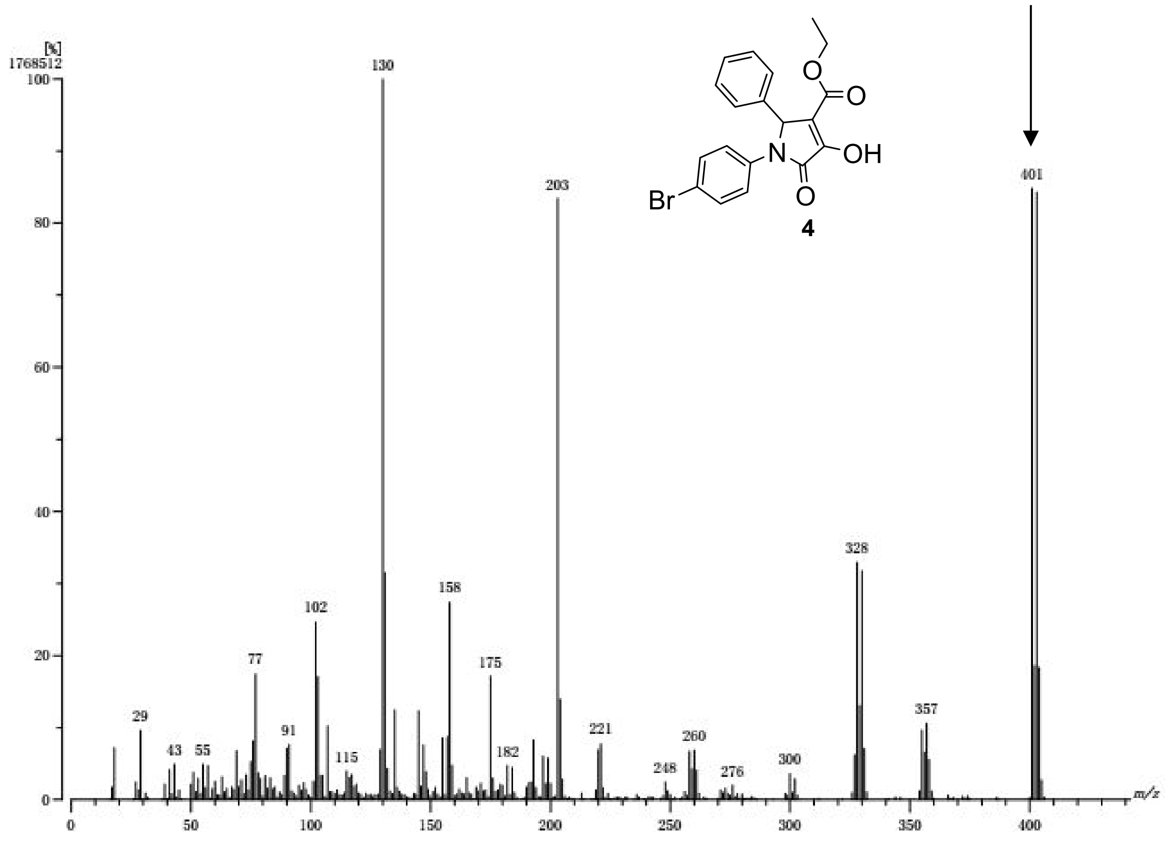

Figure S12. Electron impact mass spectra of compound **4**.

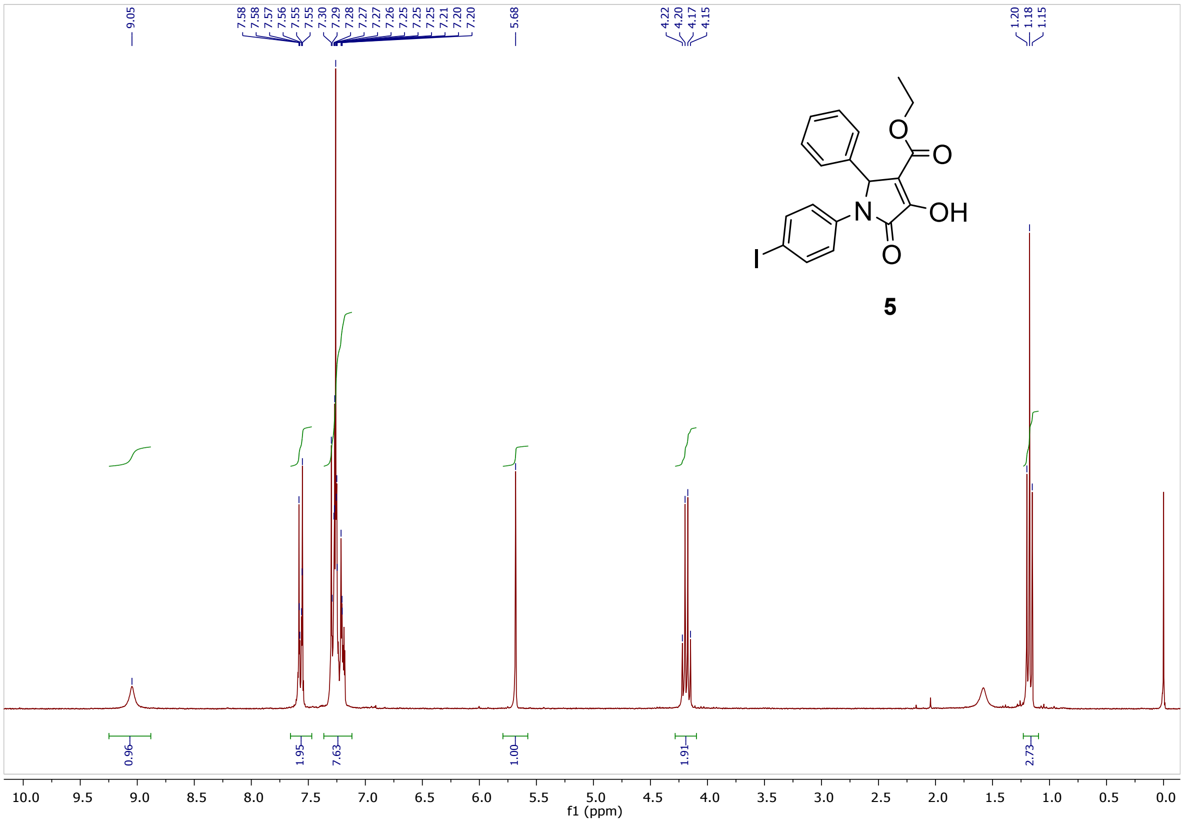

Figure S13. ^{1}H NMR spectrum of compound **5** at 300 MHz in $CDCl_3$.

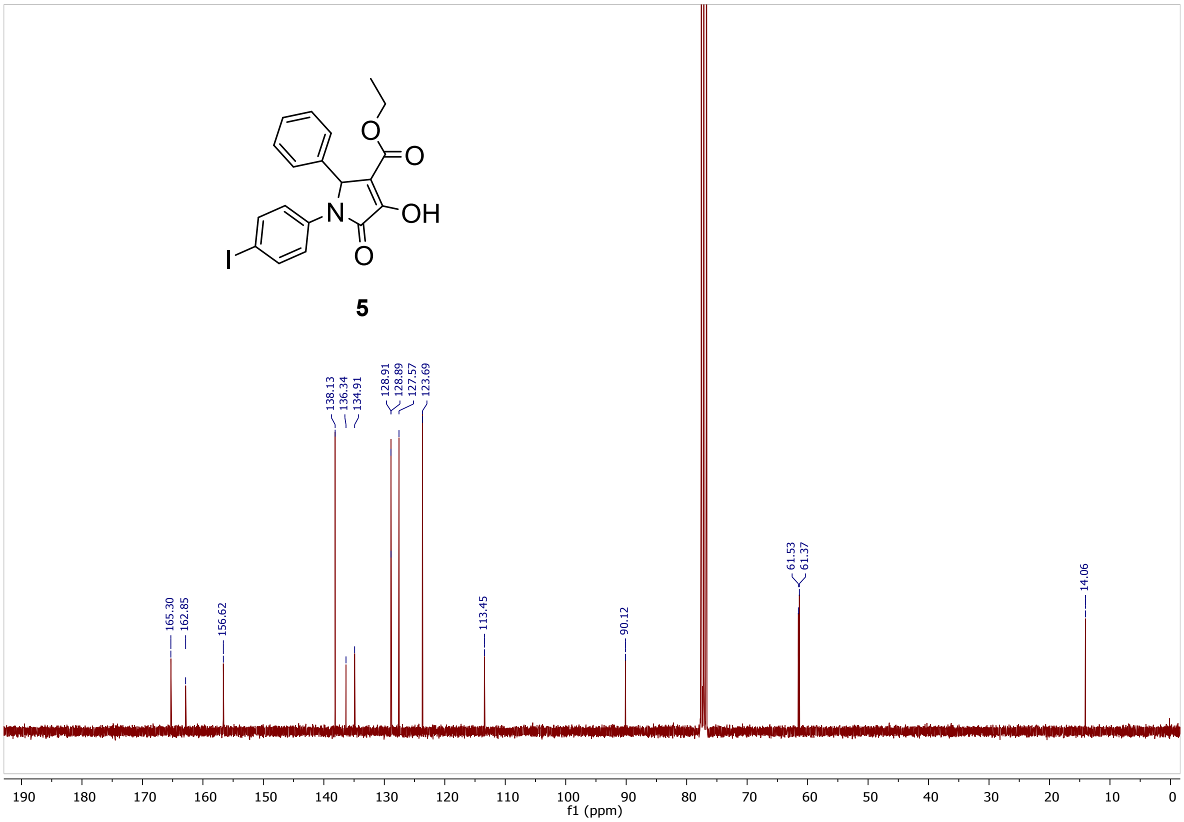

Figure S14. ^{13}C NMR spectrum of compound **5** at 75 MHz in $CDCl_3$.

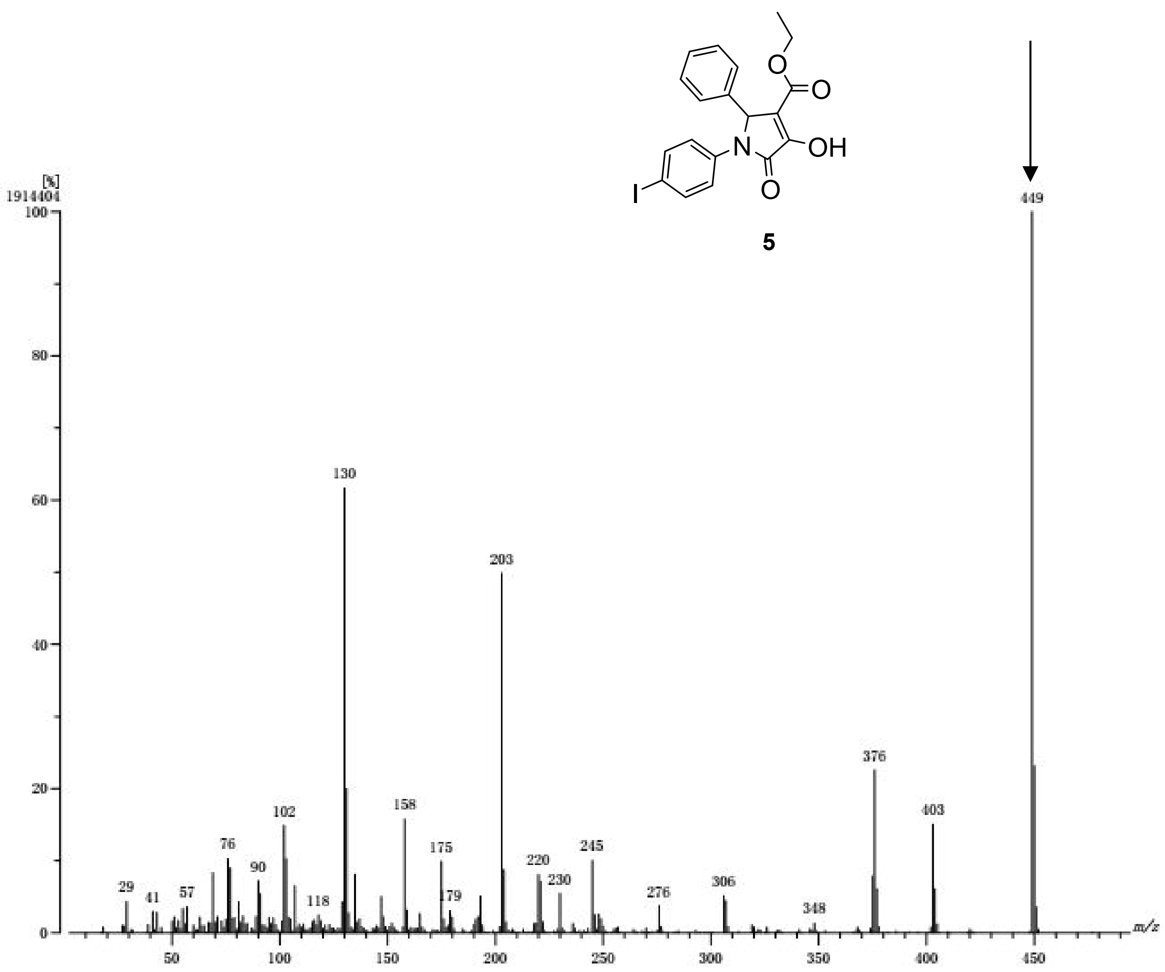

Figure S15. Electron impact mass spectra of compound **5**.

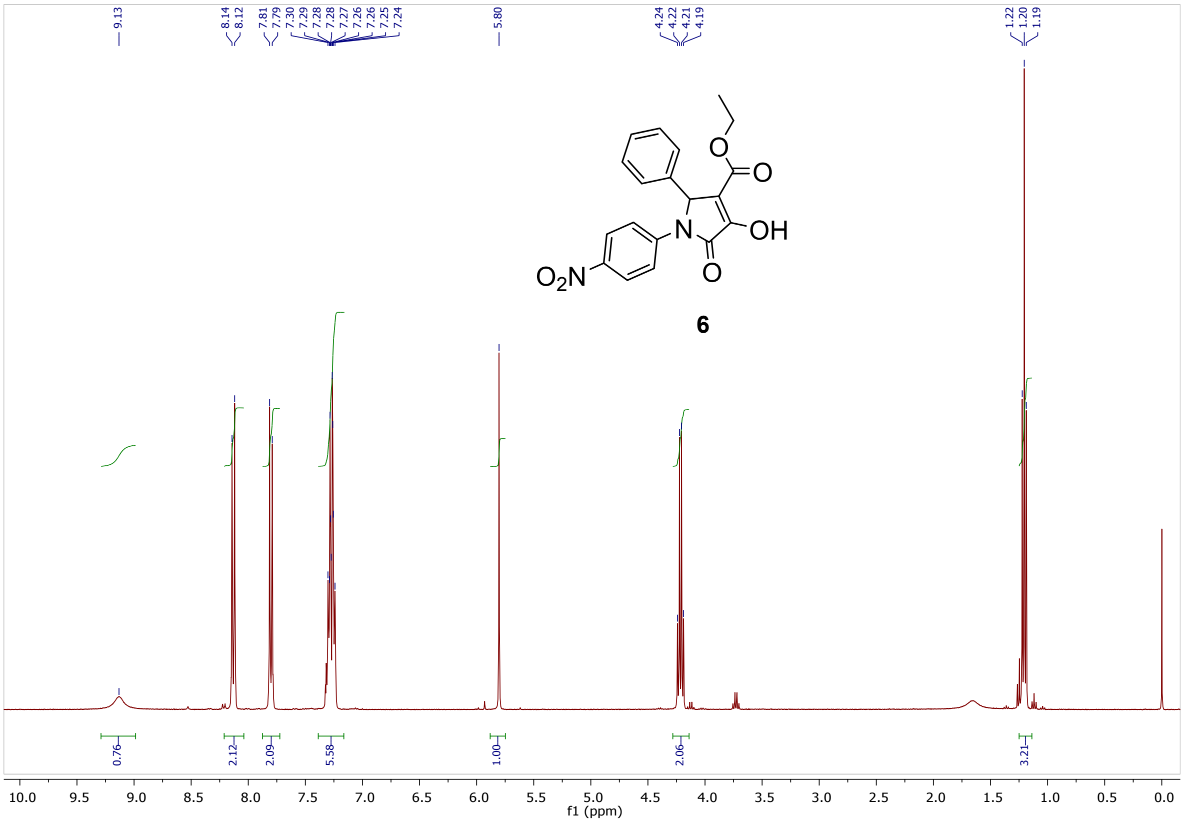

Figure S16. ^{1}H NMR spectrum of compound **6** at 400 MHz in $CDCl_3$.

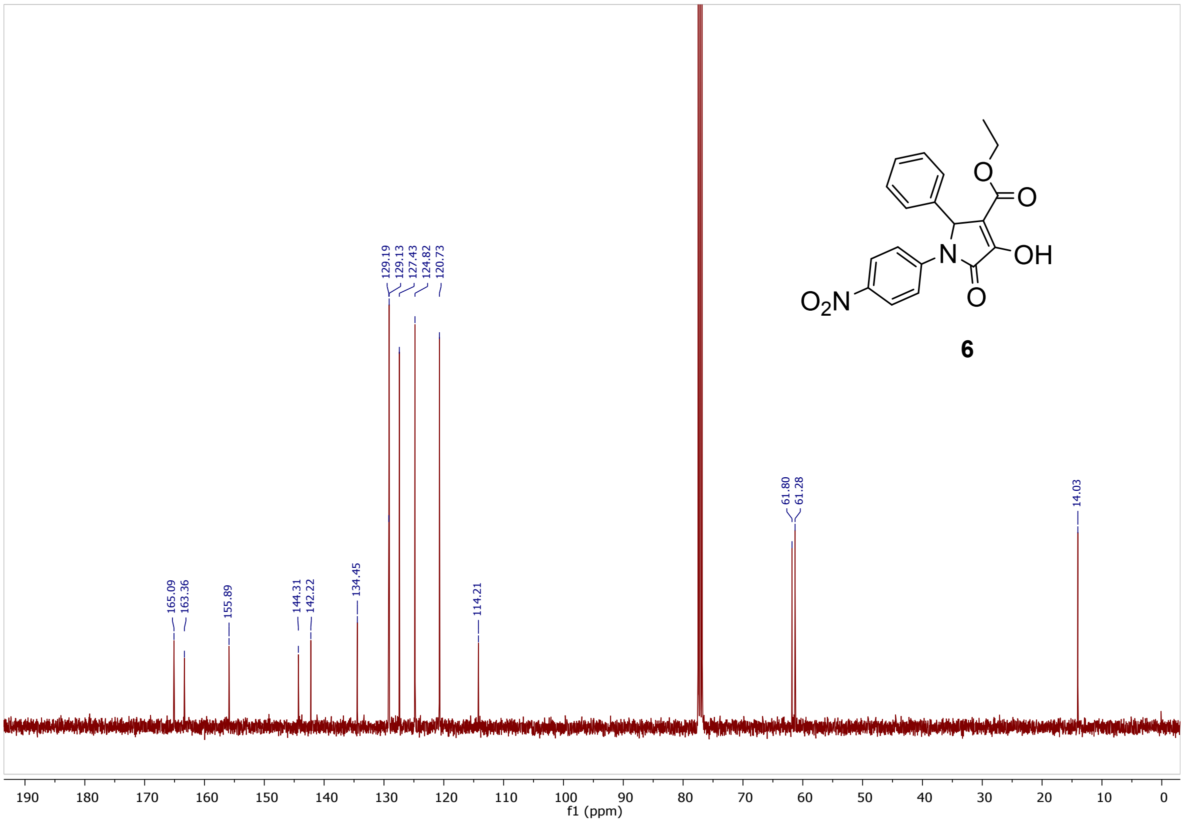

Figure S17. ^{13}C NMR spectrum of compound **6** at 100 MHz in $CDCl_3$.

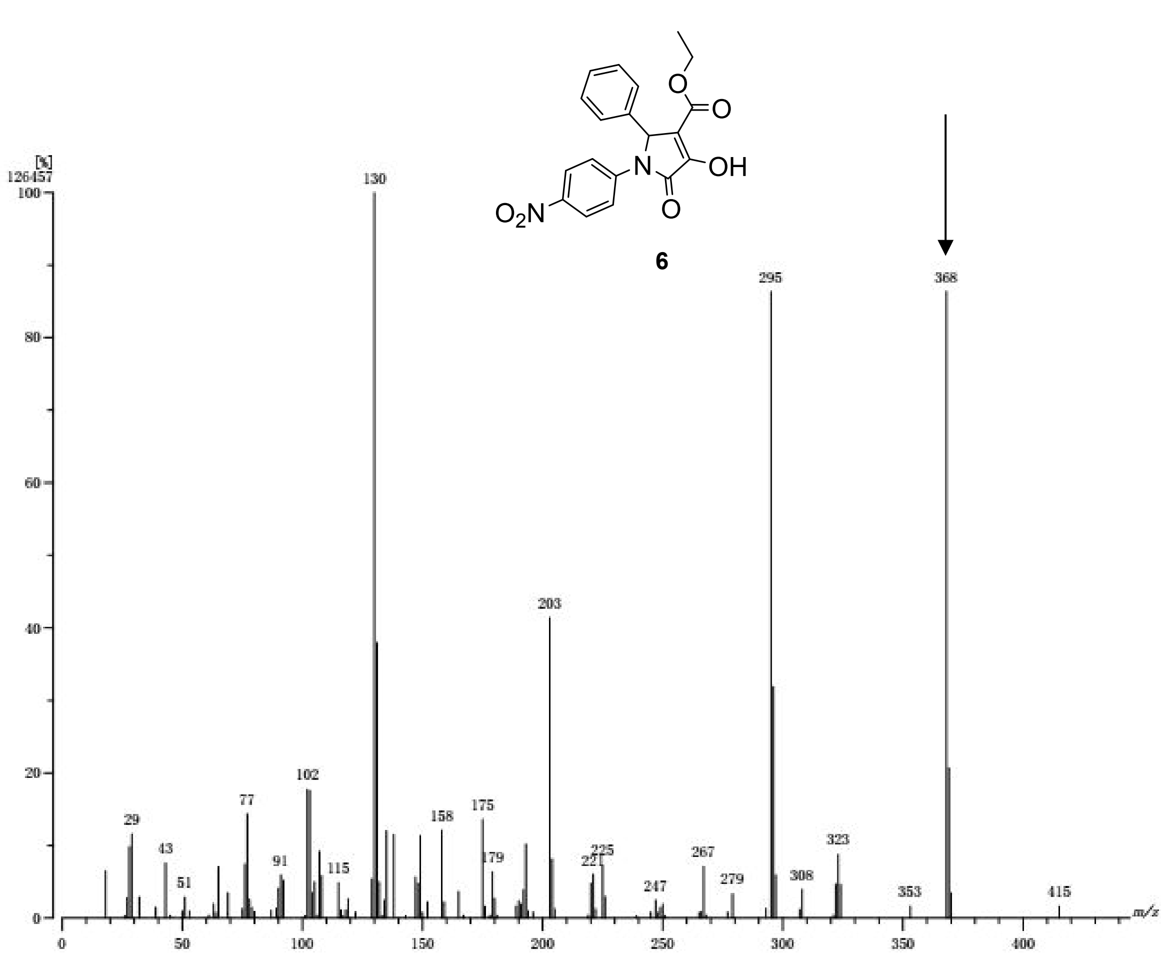

Figure S18. Electron impact mass spectra of compound **6**.

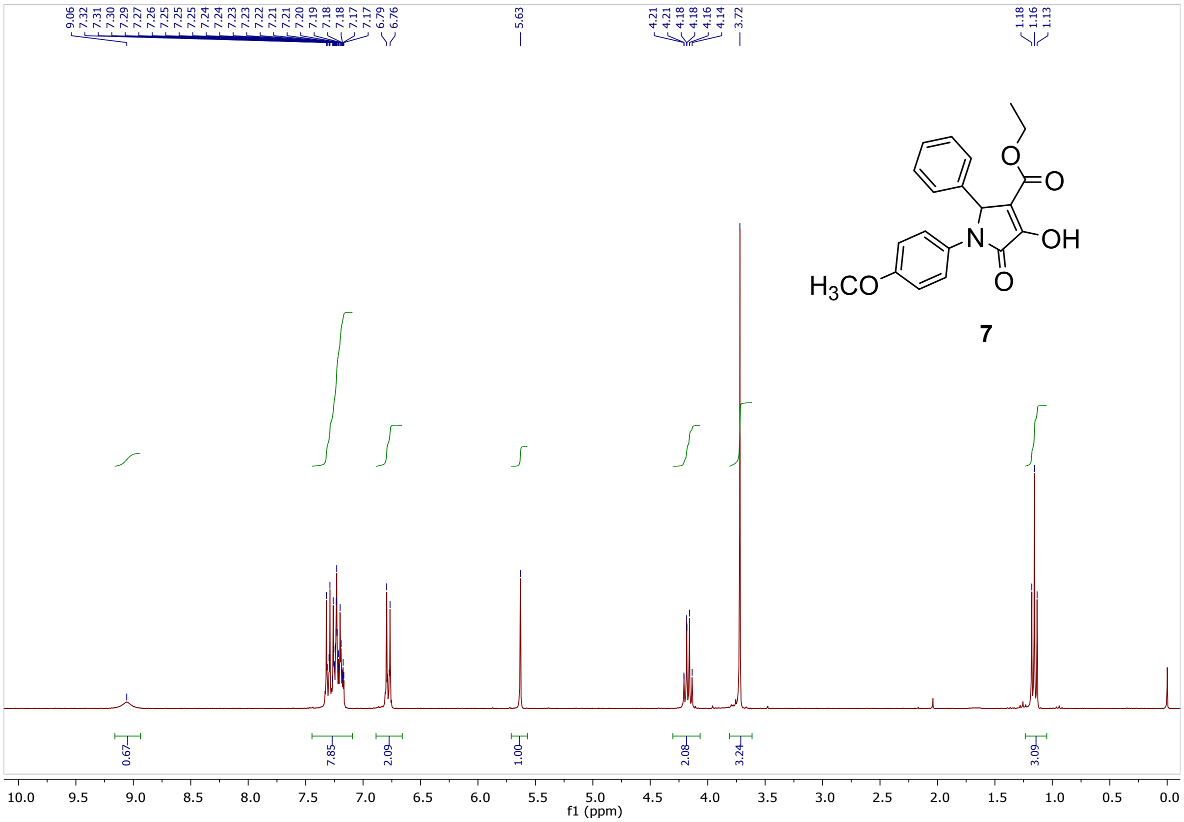

Figure S19. ^{1}H NMR spectrum of compound **7** at 300 MHz in $CDCl_3$.

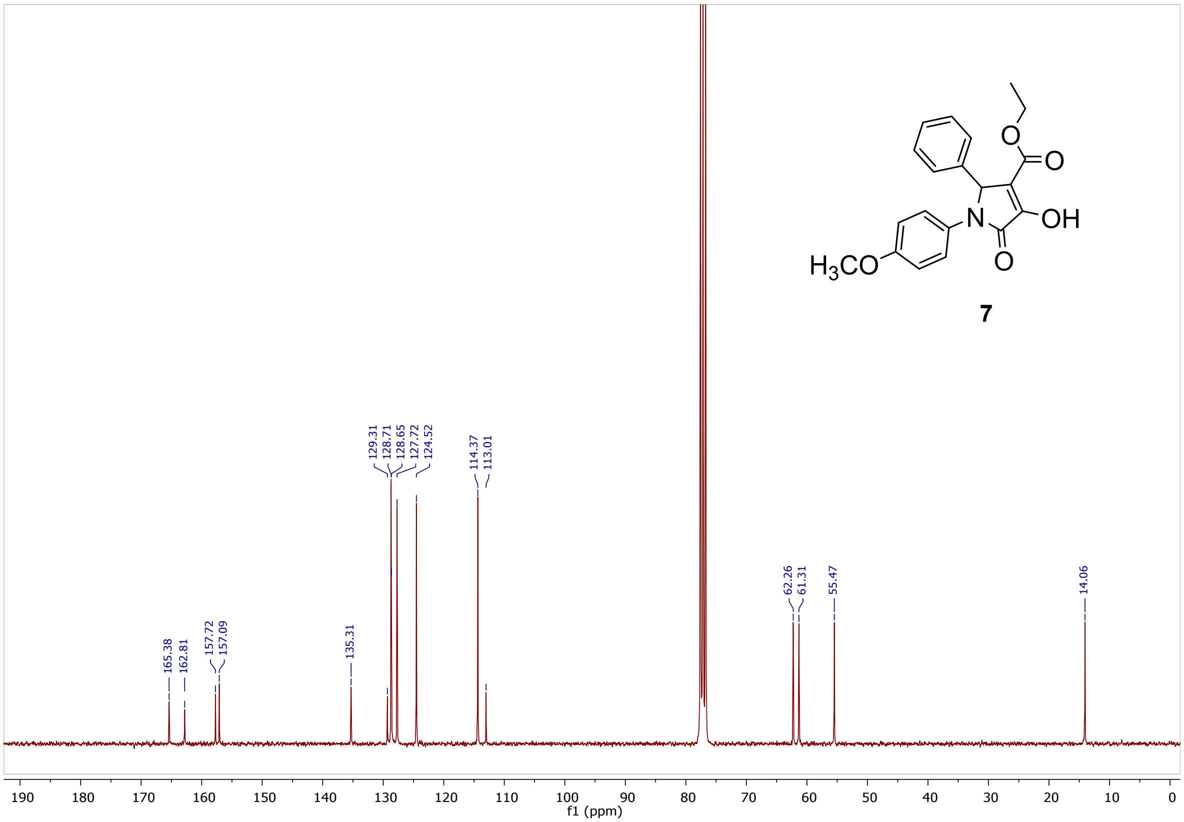

Figure S20. ^{13}C NMR spectrum of compound **7** at 75 MHz in $CDCl_3$.

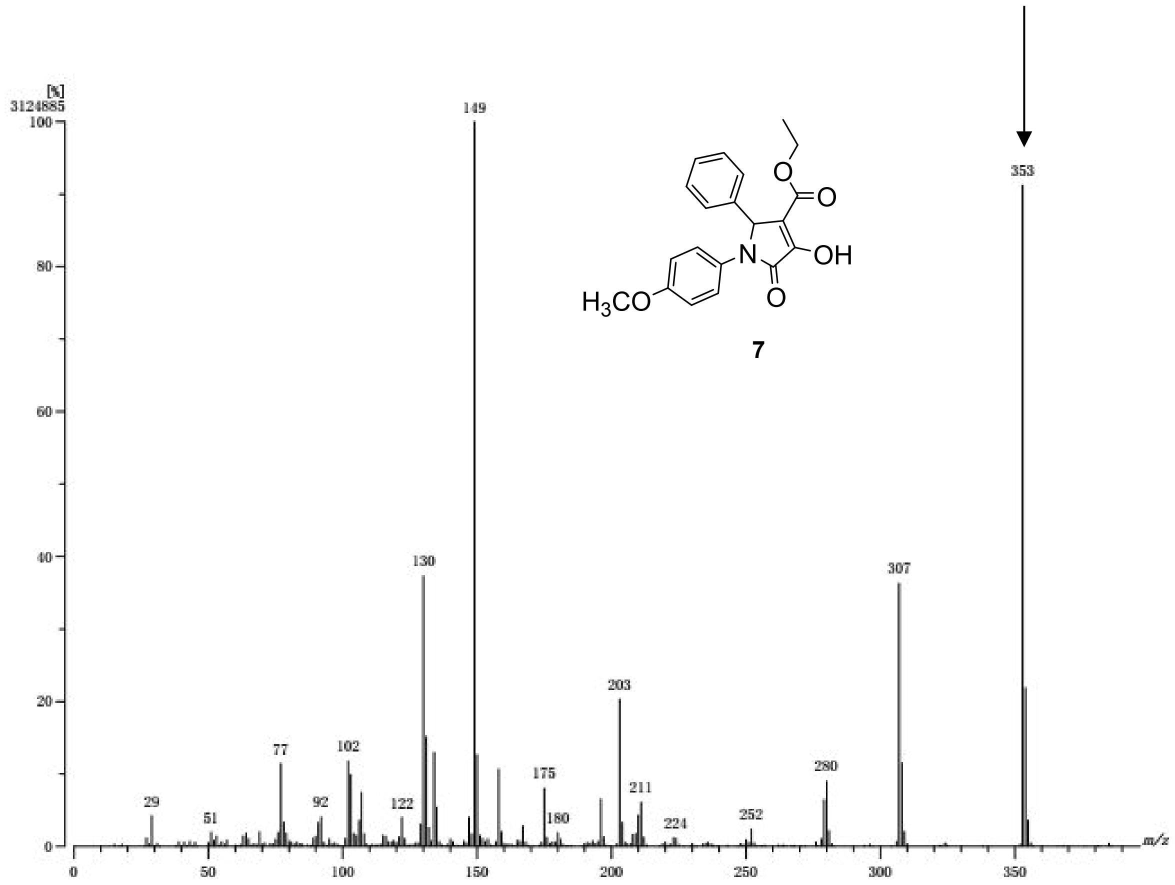

Figure S21. Electron impact mass spectra of compound **7**.

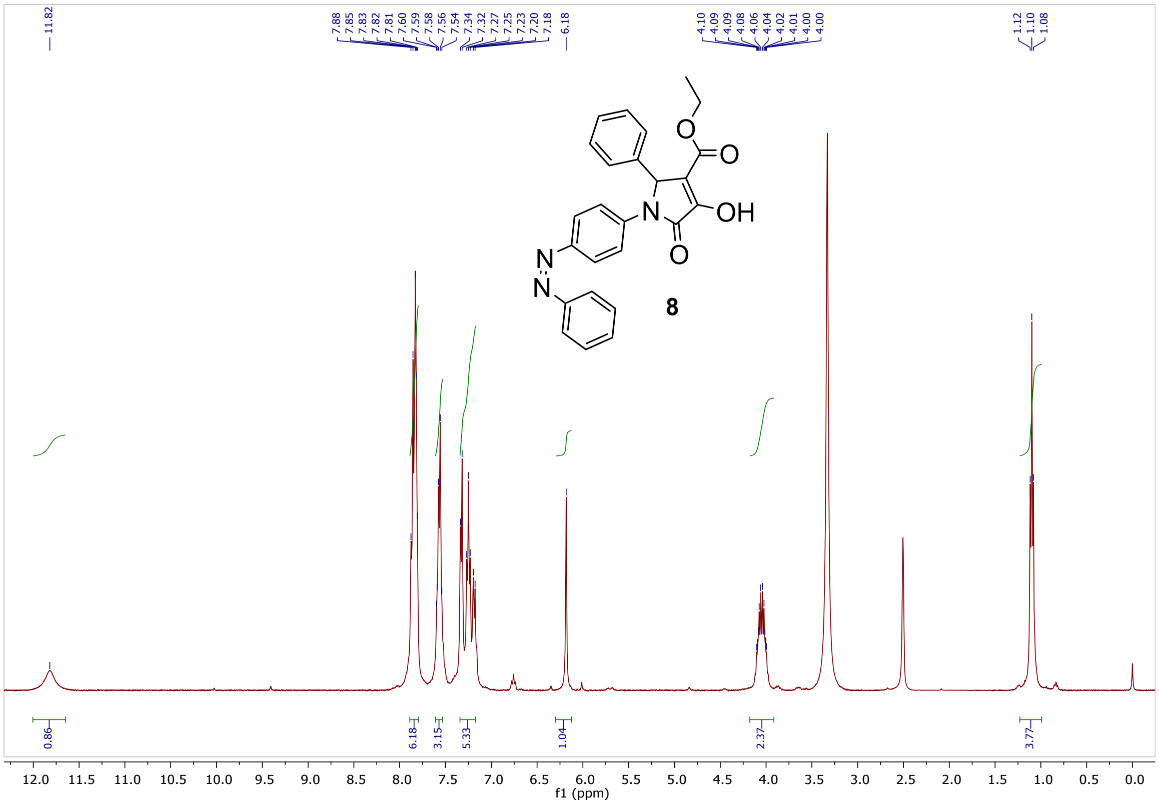

Figure S22. ^{1}H NMR spectrum of compound **8** at 300 MHz in DMSO-d_6.

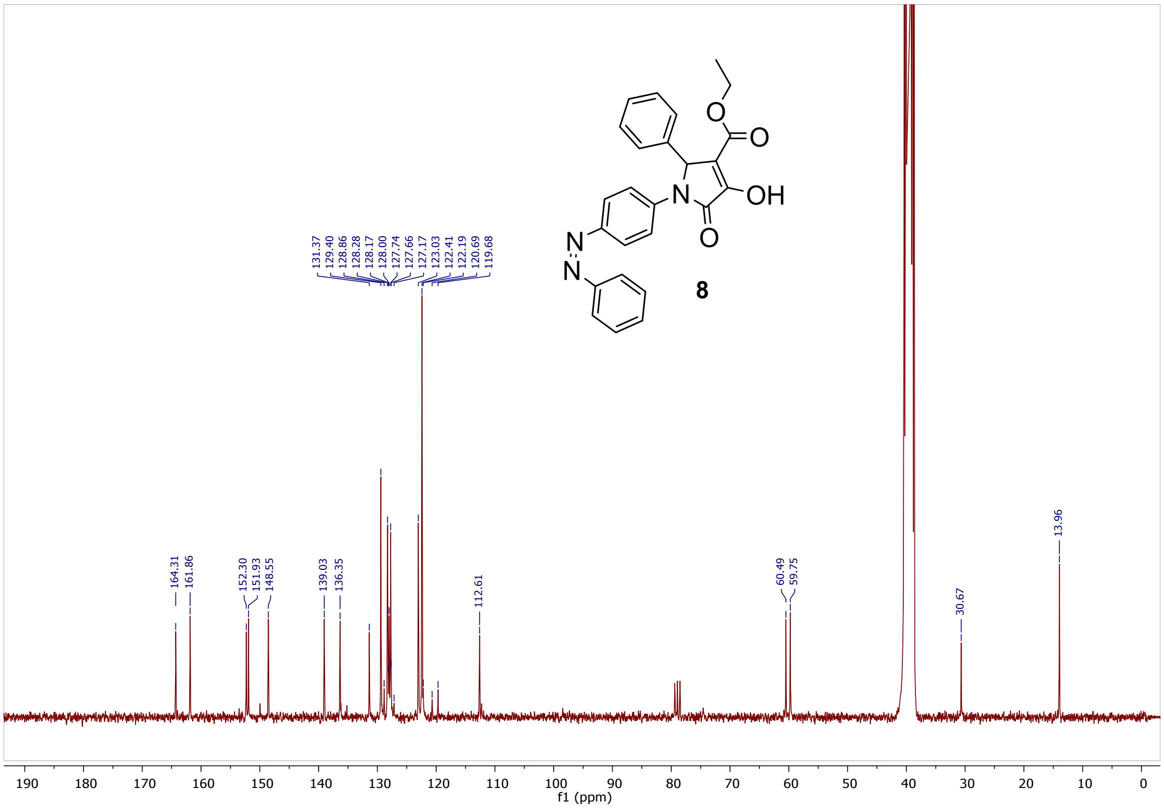

Figure S23. ^{13}C NMR spectrum of compound **8** at 75 MHz in DMSO-d_6.

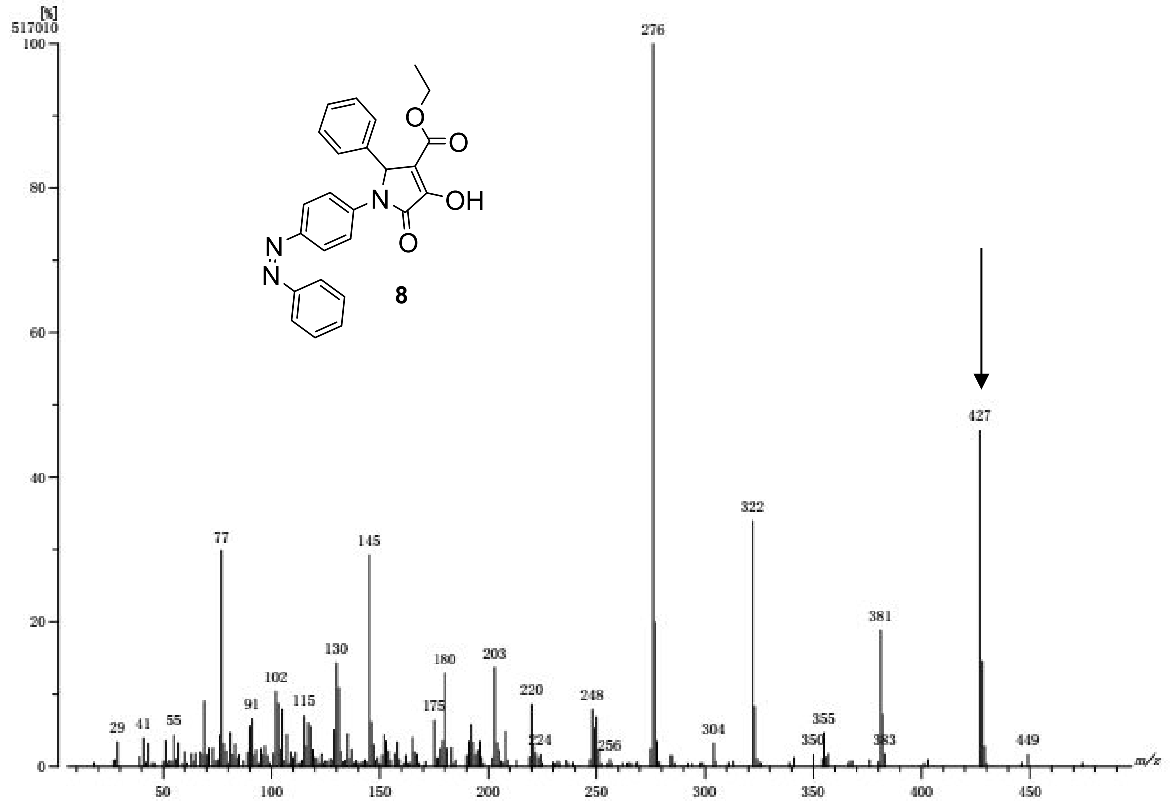

Figure S24. Electron impact mass spectra of compound **8**.

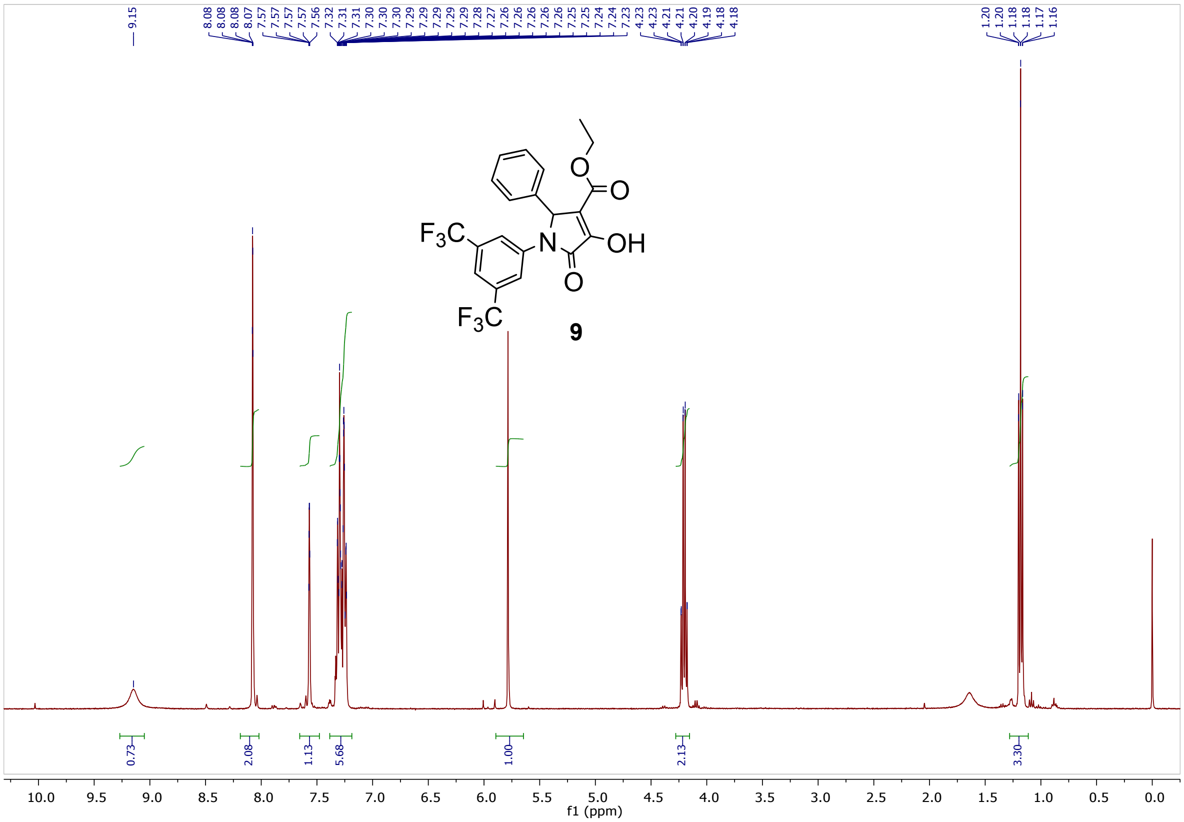

Figure S25. ^{1}H NMR spectrum of compound **9** at 400 MHz in $CDCl_3$.

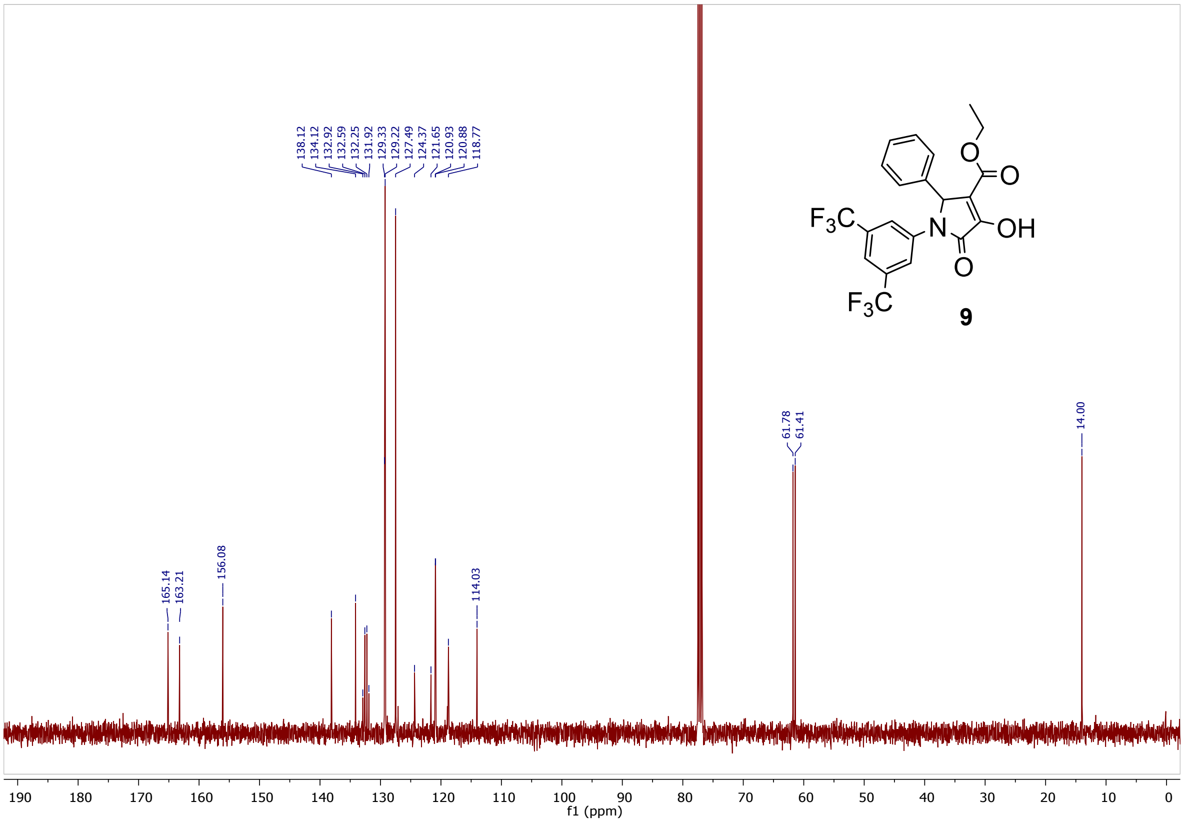

Figure S26. ^{13}C NMR spectrum of compound **9** at 100 MHz in $CDCl_3$.

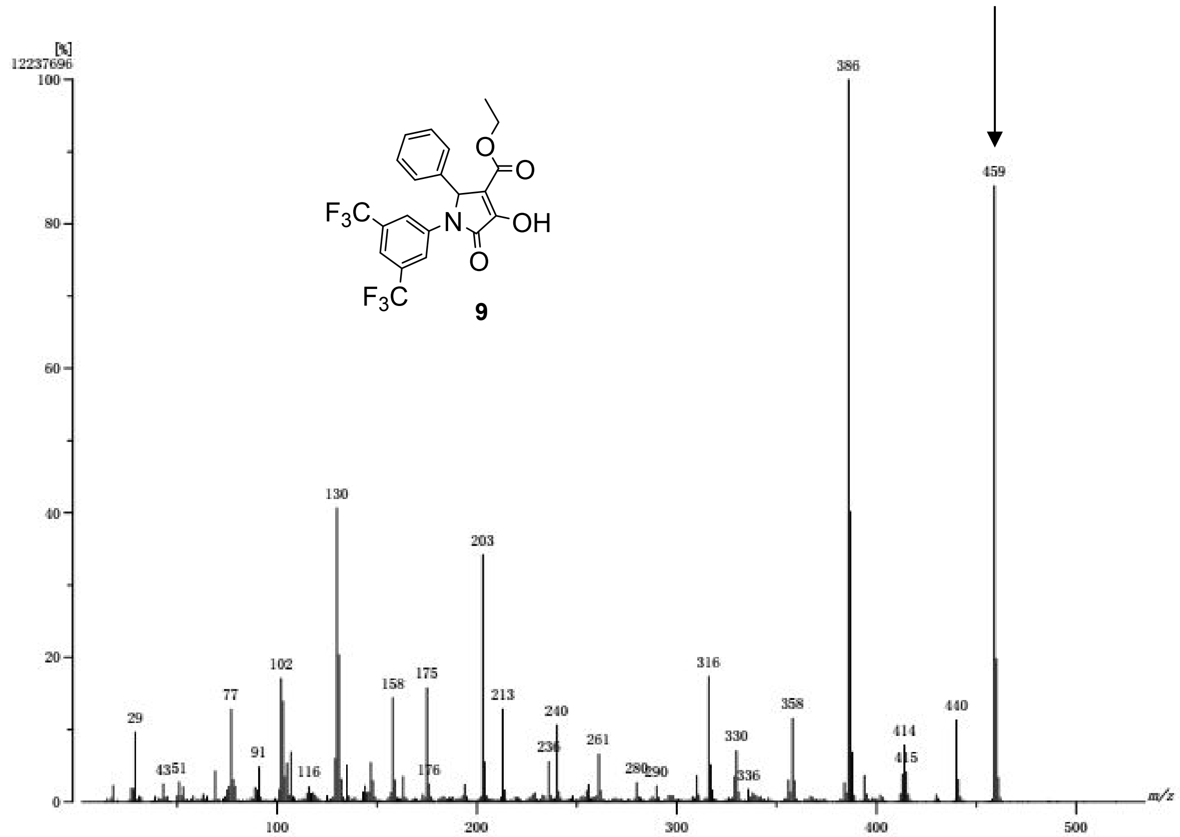

Figure S27. Electron impact mass spectra of compound **9**.

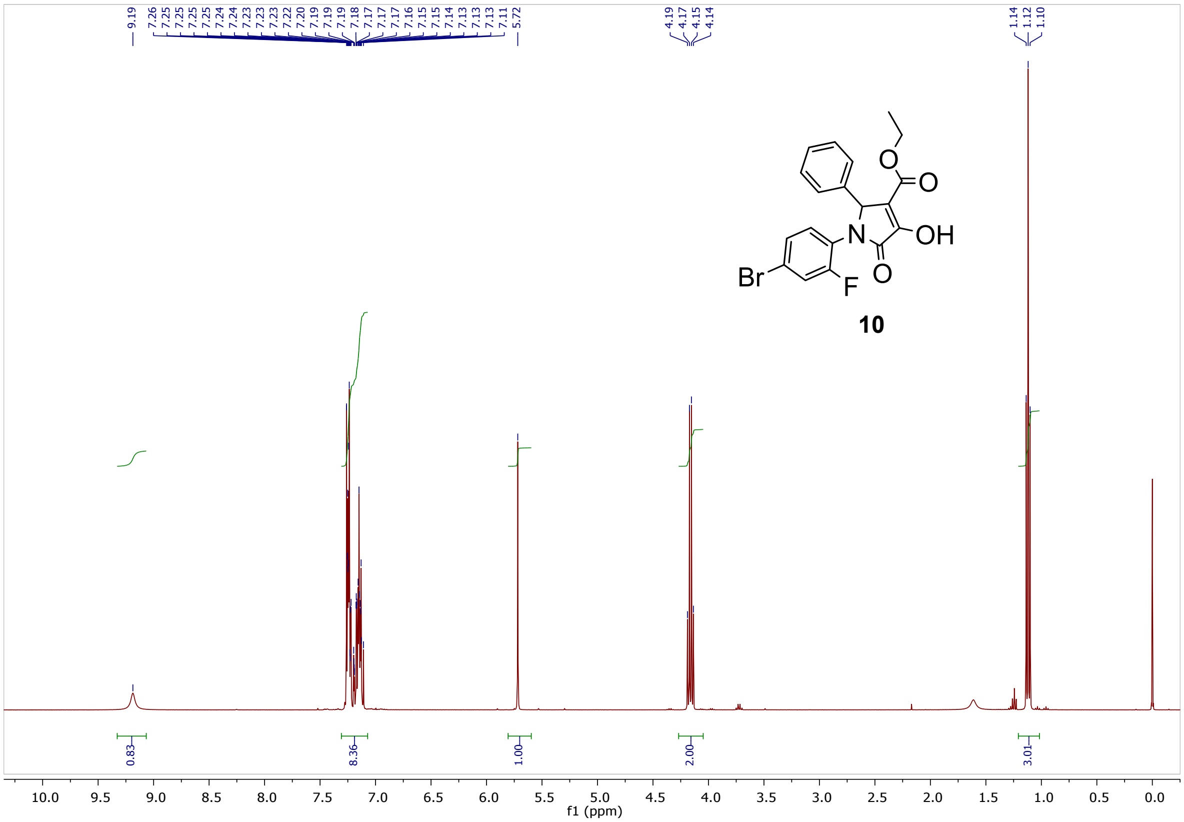

Figure S28. ^{1}H NMR spectrum of compound **10** at 400 MHz in $CDCl_3$.

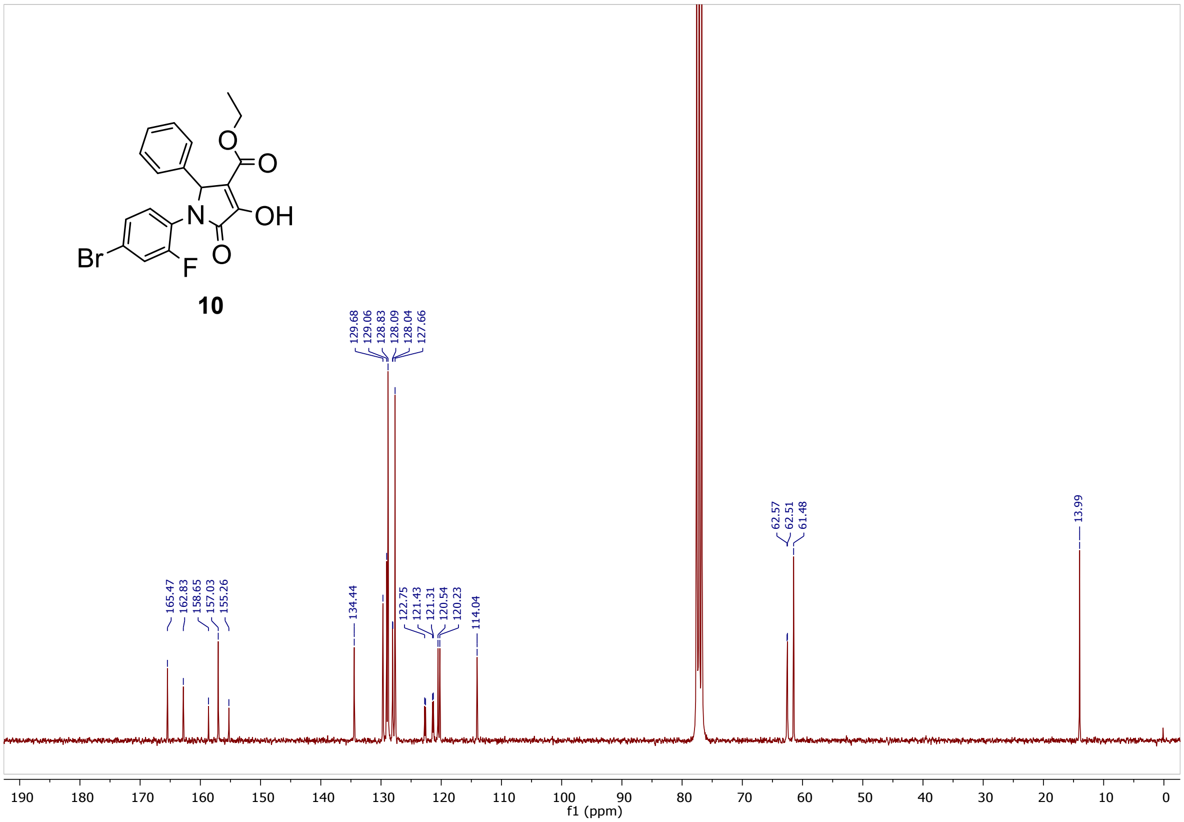

Figure S29. ^{13}C NMR spectrum of compound **10** at 100 MHz in $CDCl_3$.

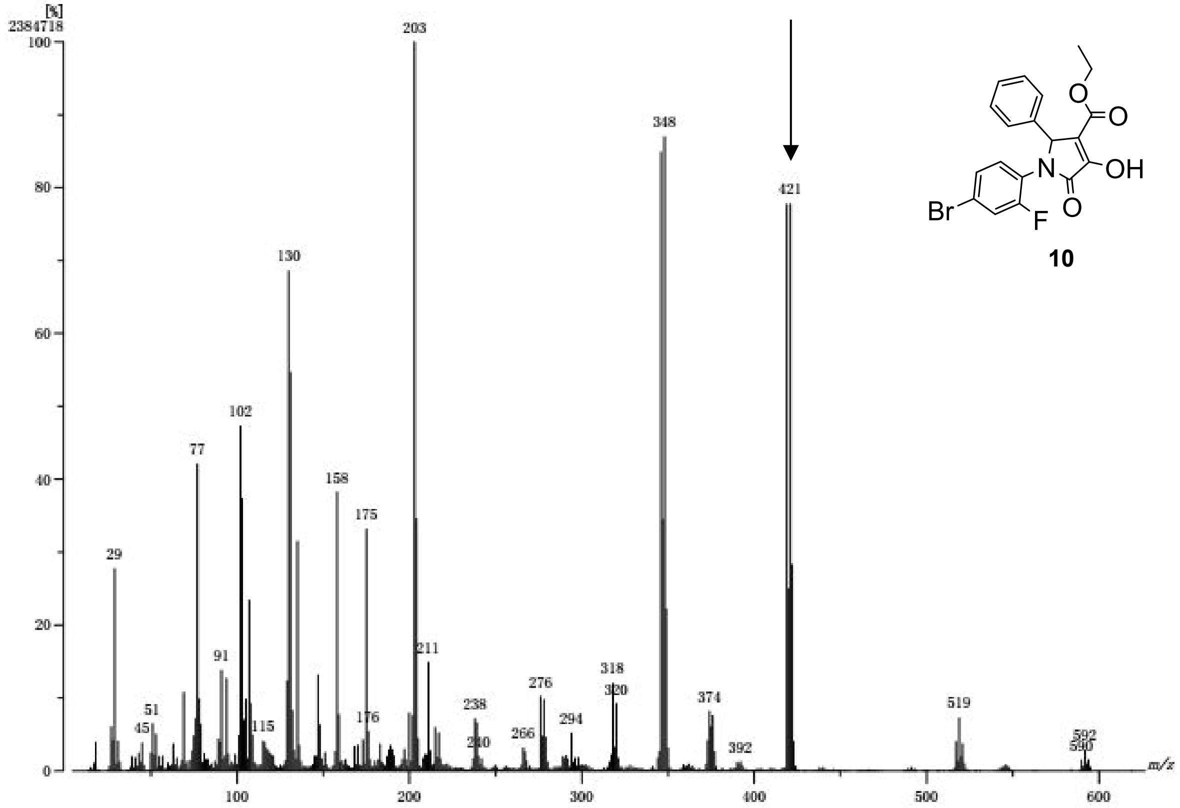

Figure S30. Electron impact mass spectra of compound **10**.

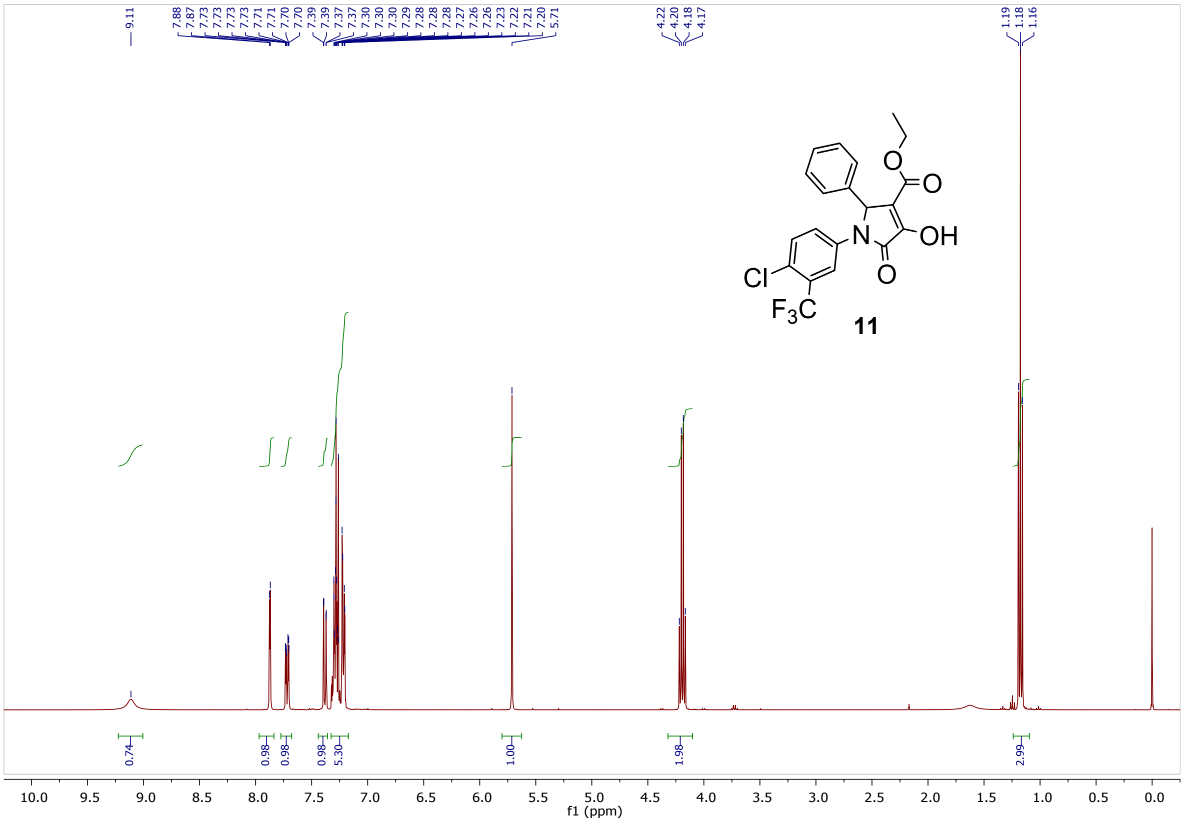

Figure S31. ^{1}H NMR spectrum of compound **11** at 400 MHz in $CDCl_3$.

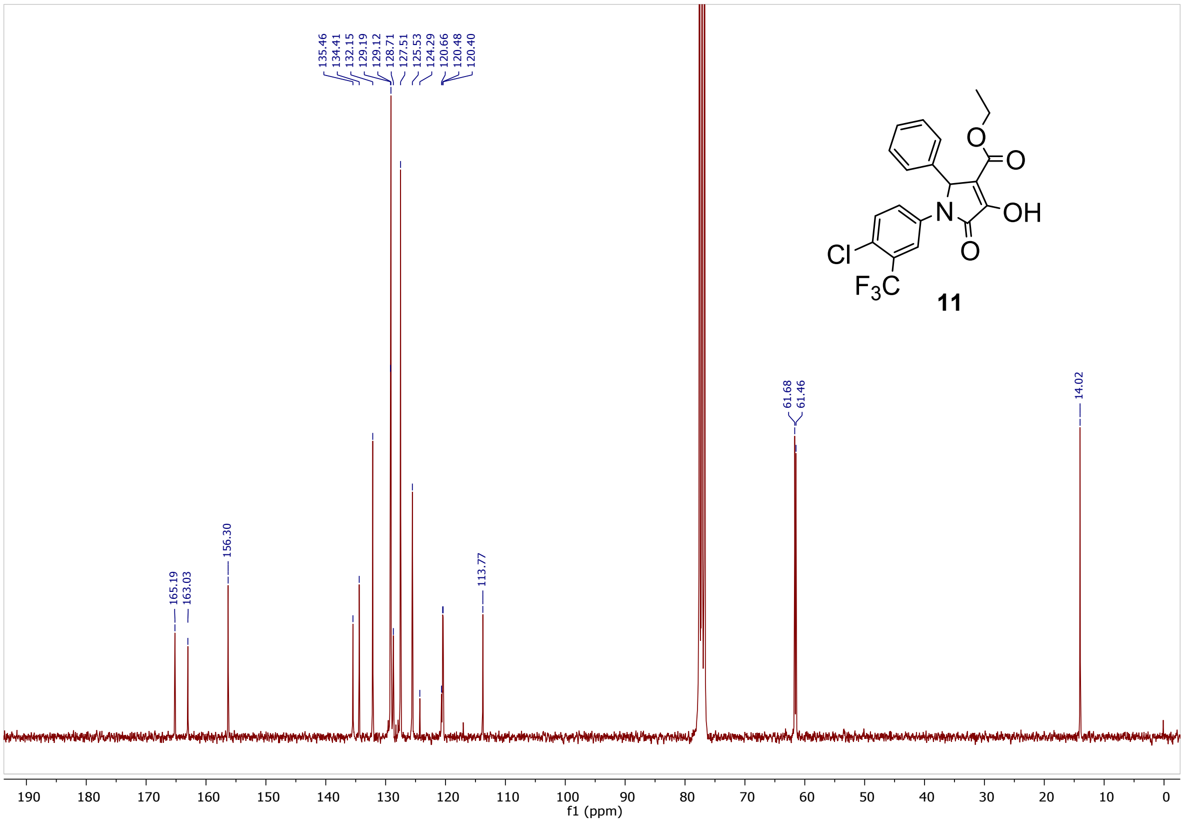

Figure S32. ^{13}C NMR spectrum of compound **11** at 100 MHz in $CDCl_3$.

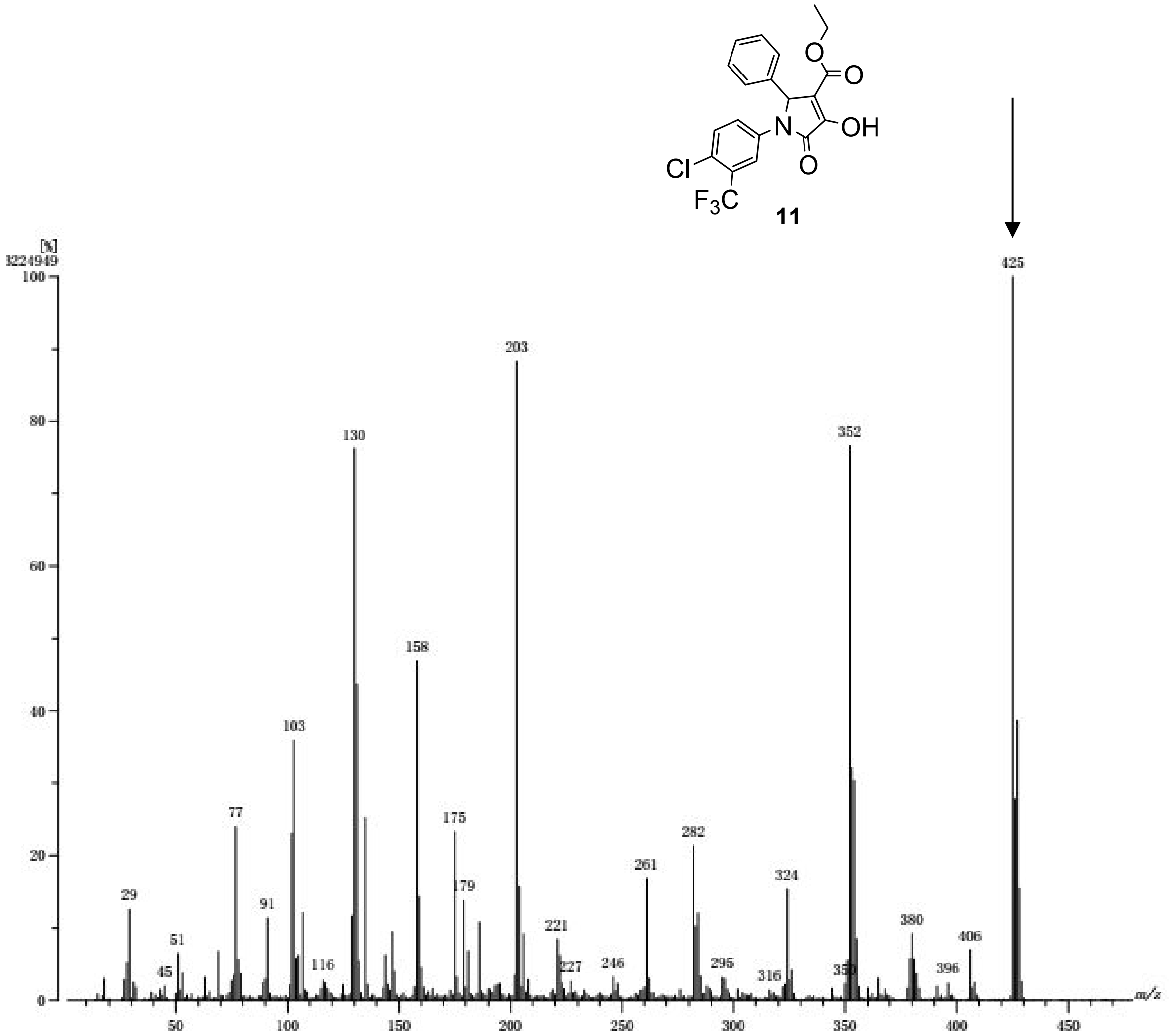

Figure S33. Electron impact mass spectra of compound **11**.

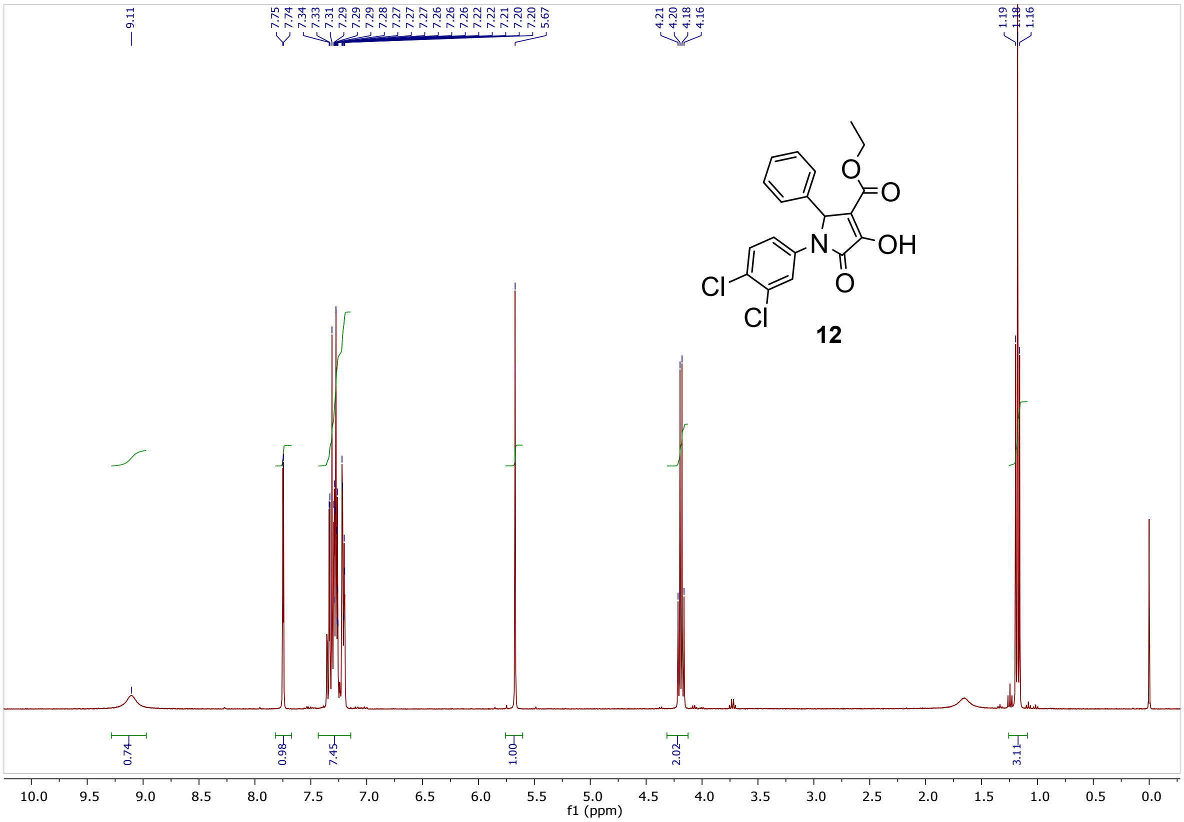

Figure S34. ^{1}H NMR spectrum of compound **12** at 400 MHz in $CDCl_3$.

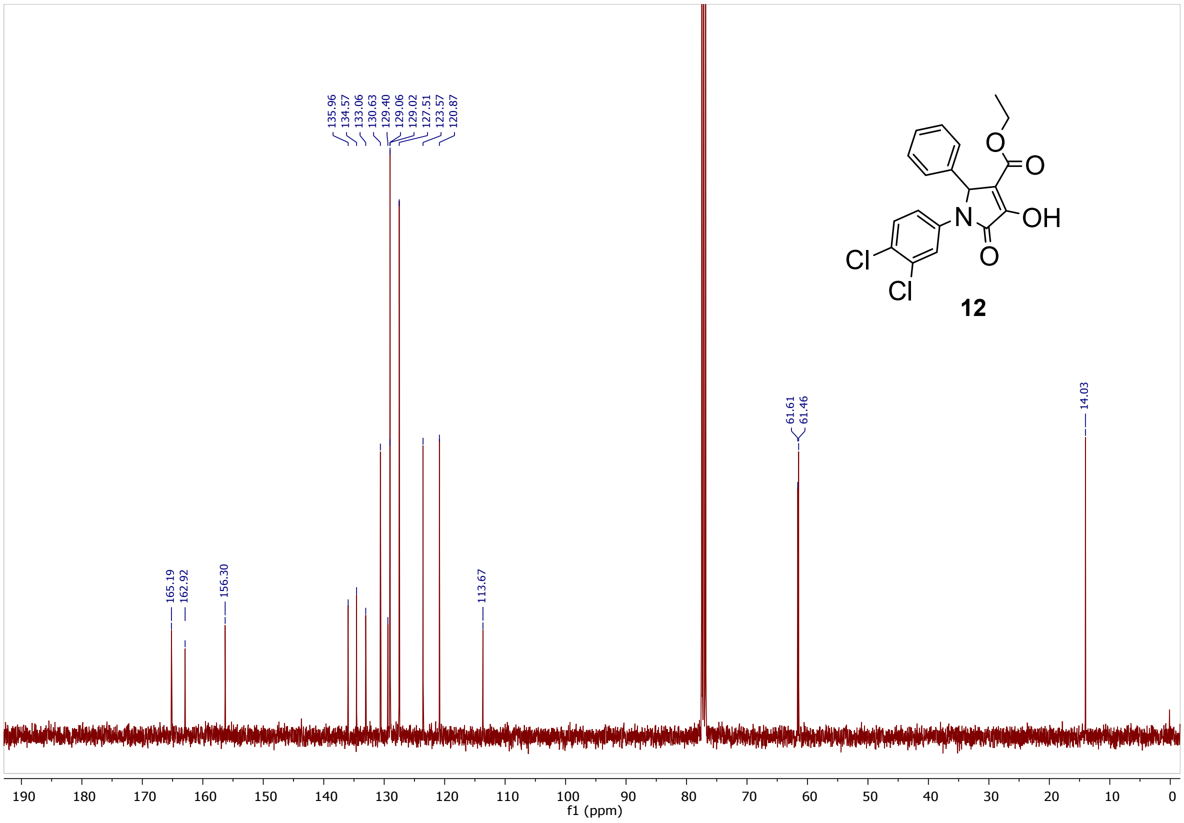

Figure S35. ^{13}C NMR spectrum of compound **12** at 100 MHz in $CDCl_3$.

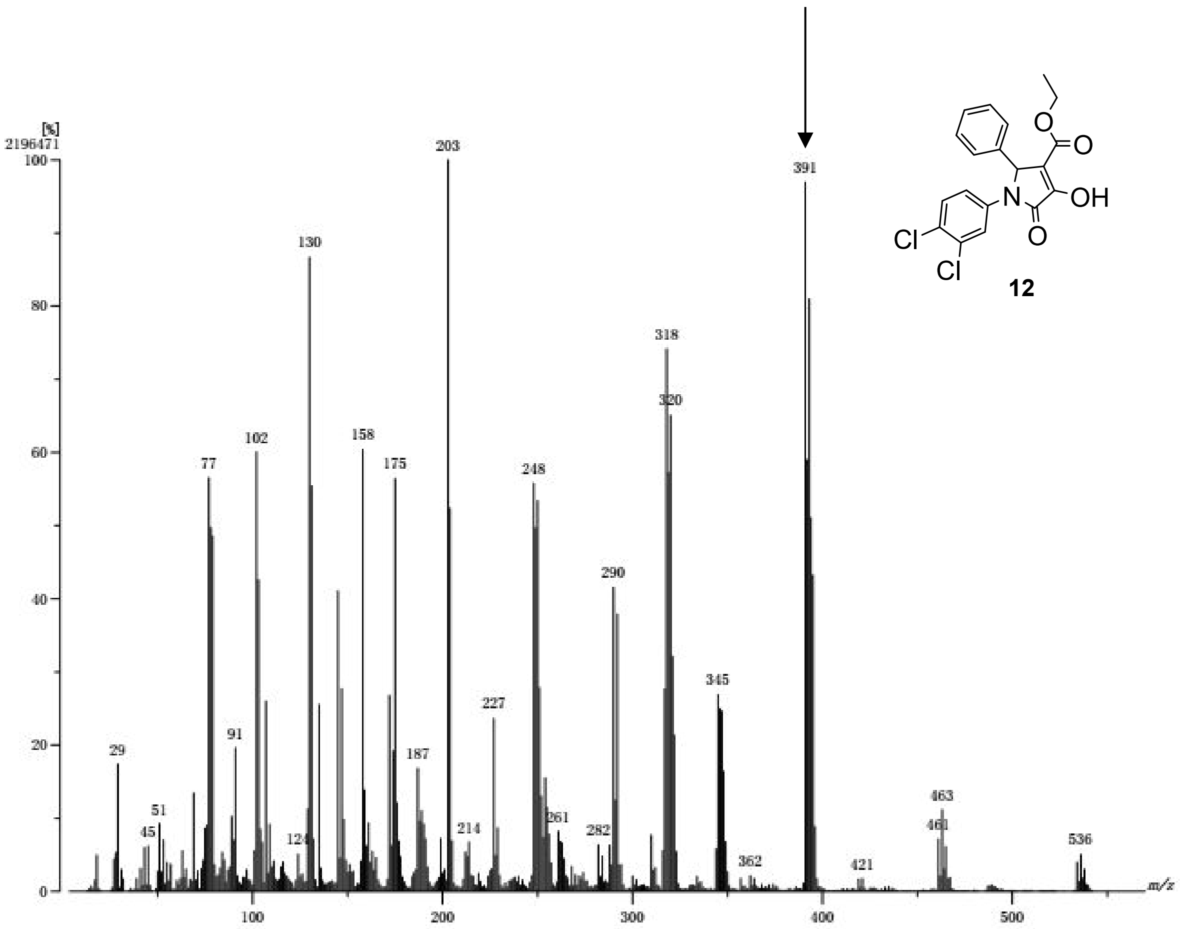

12

Figure S36. Electron impact mass spectra of compound **12**.

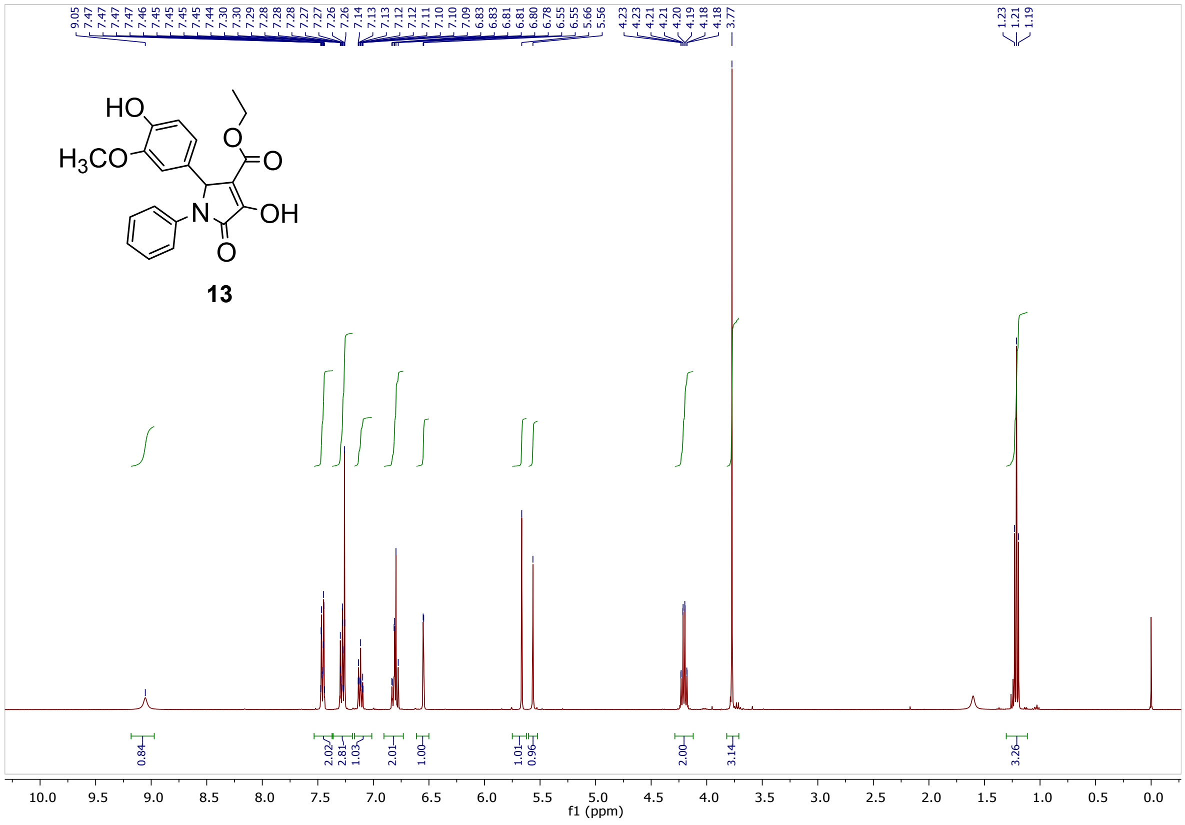

Figure S37. ^{1}H NMR spectrum of compound **13** at 300 MHz in $CDCl_3$.

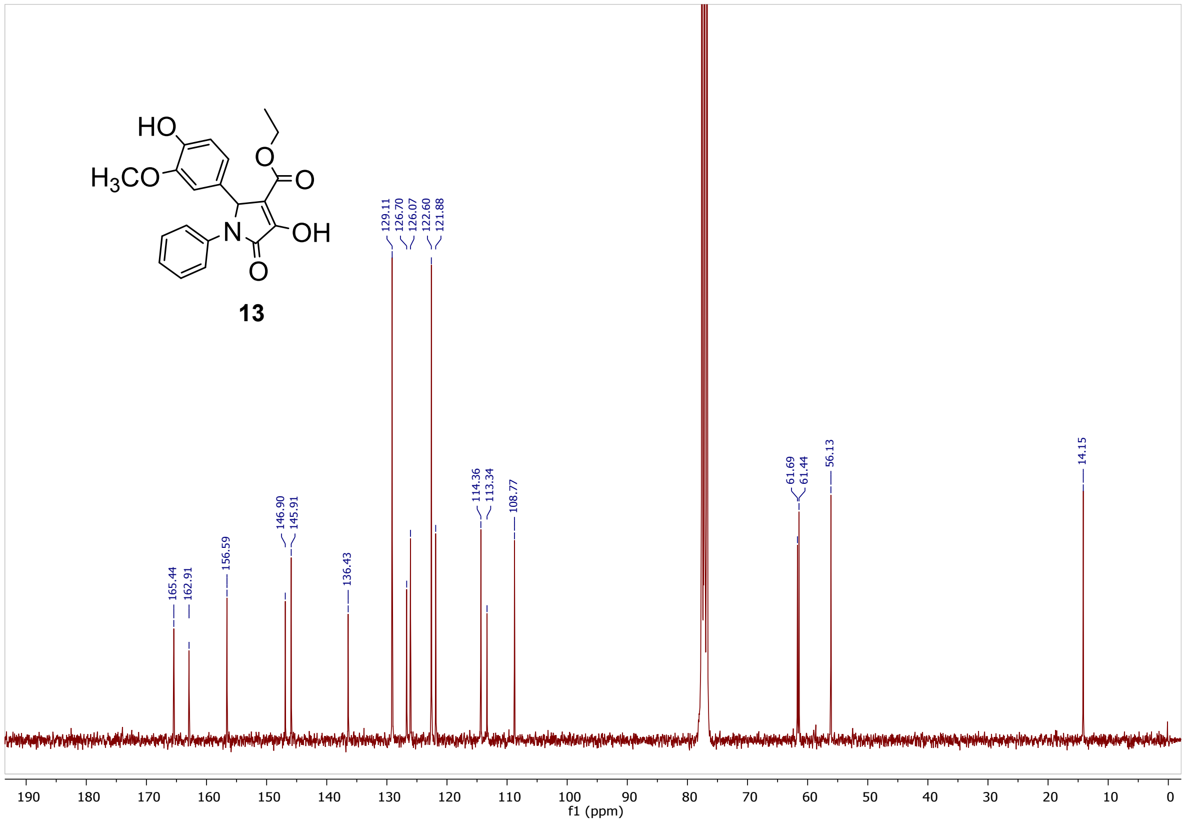

Figure S38. ^{13}C NMR spectrum of compound **13** at 75 MHz in $CDCl_3$.

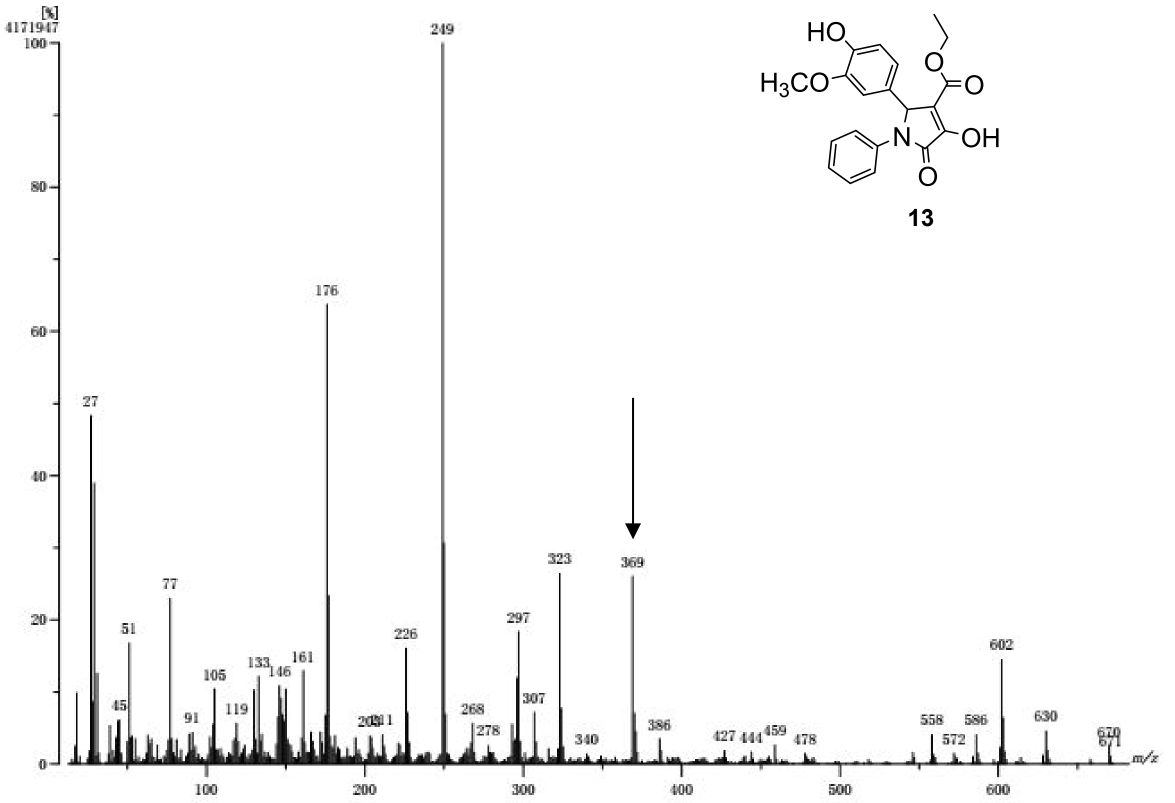

Figure S39. Electron impact mass spectra of compound **13**.

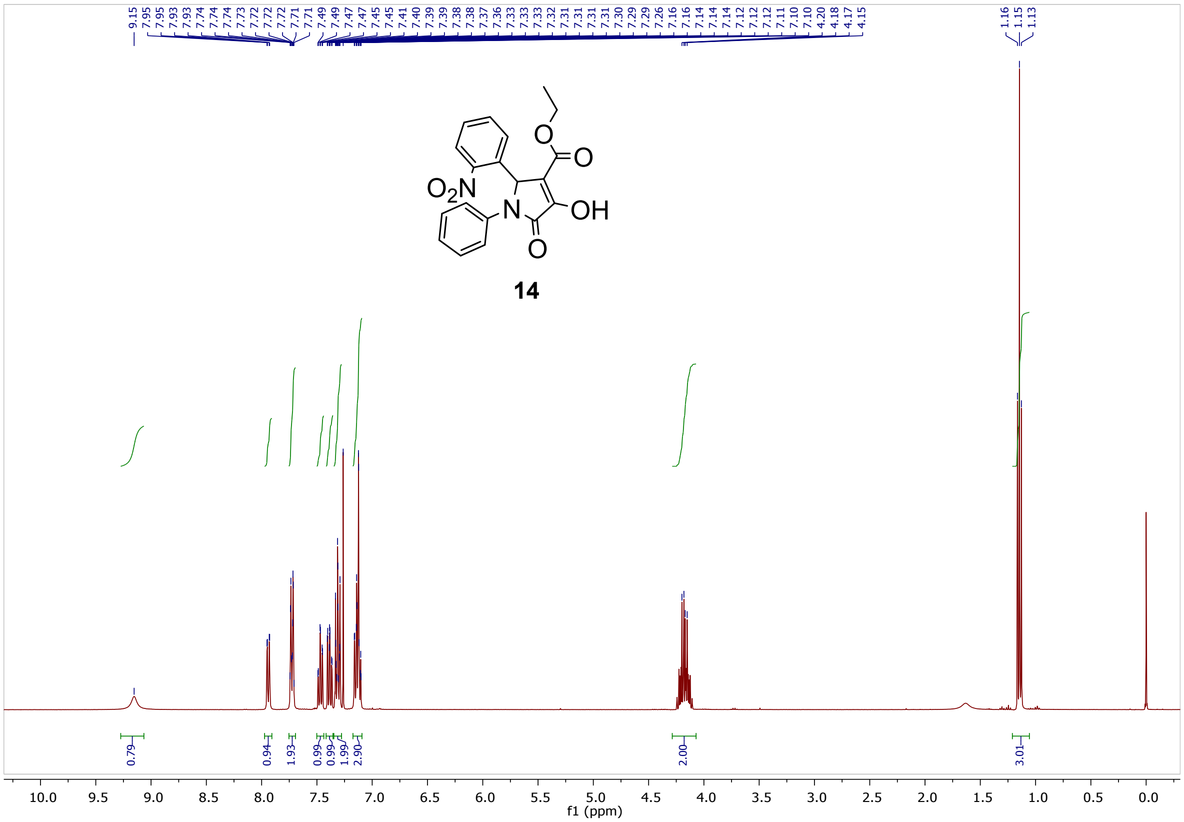

Figure S40. ^{1}H NMR spectrum of compound **14** at 400 MHz in $CDCl_3$.

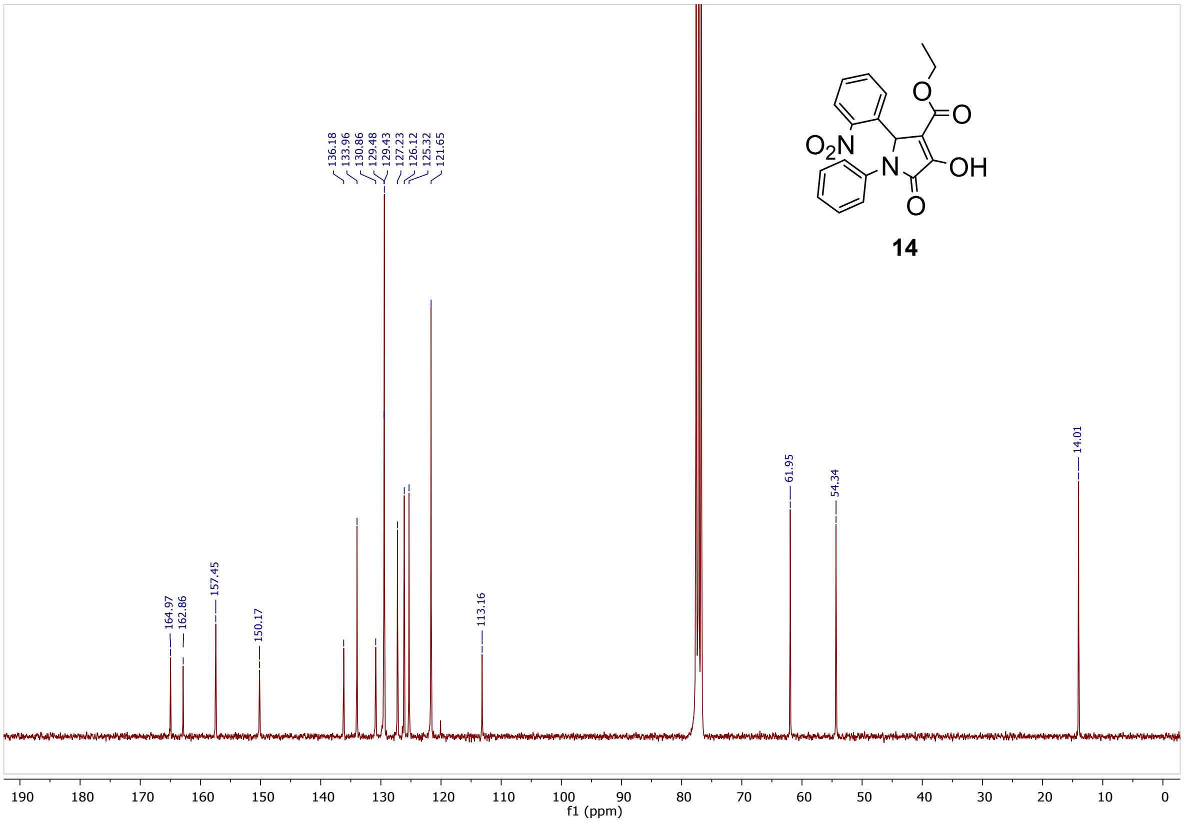

Figure S41. ^{13}C NMR spectrum of compound **14** at 100 MHz in $CDCl_3$.

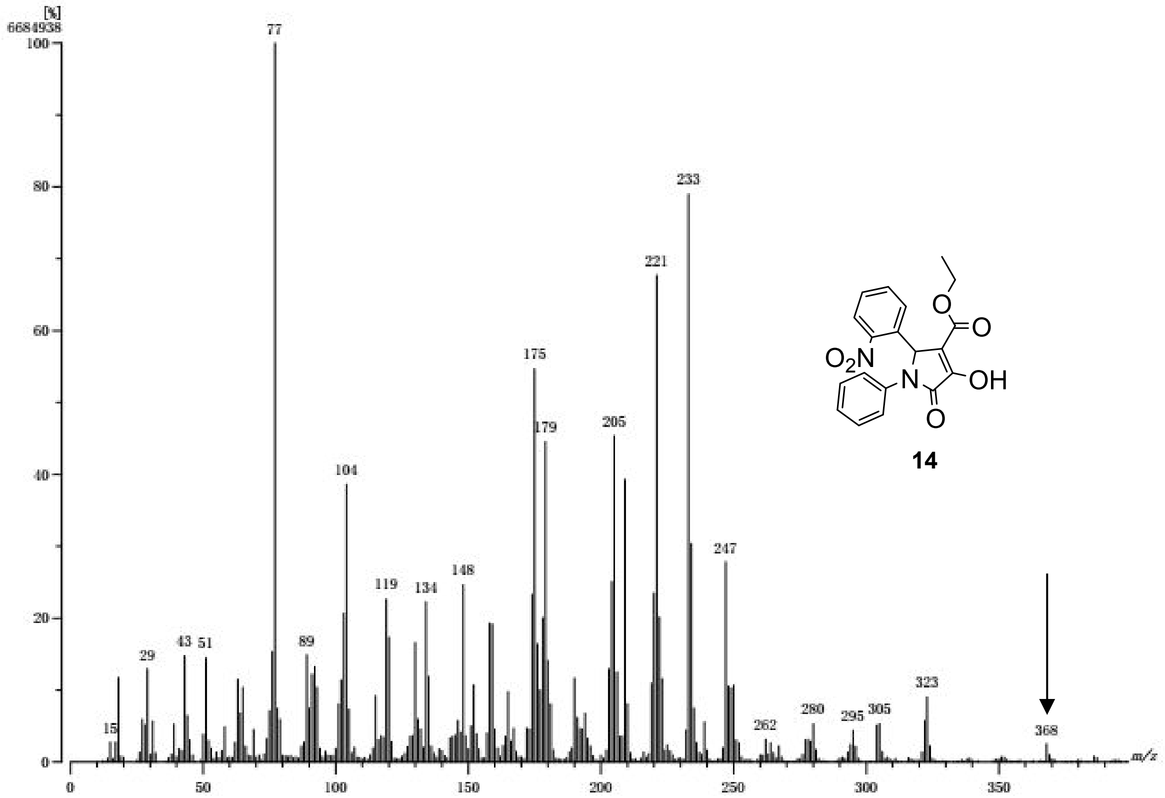

Figure S42. Electron impact mass spectra of compound **14**.

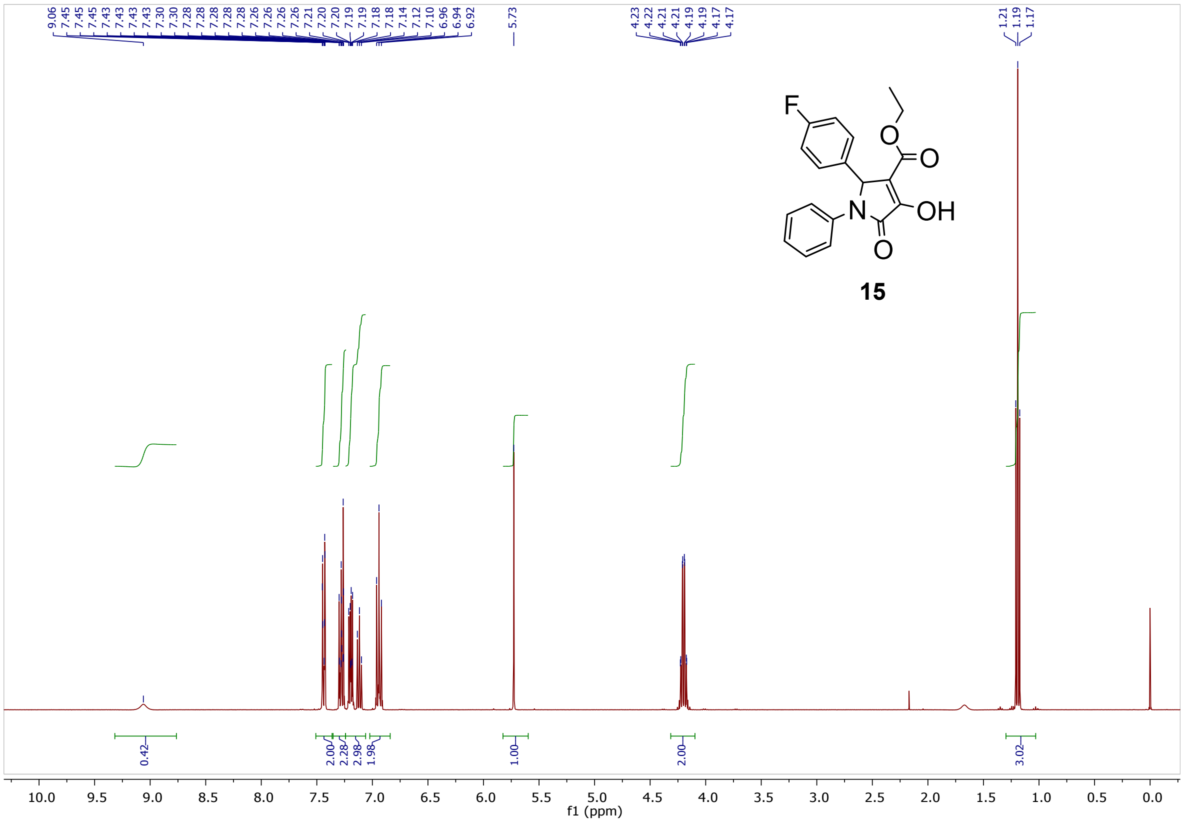

Figure S43. ^{1}H NMR spectrum of compound **15** at 300 MHz in $CDCl_3$.

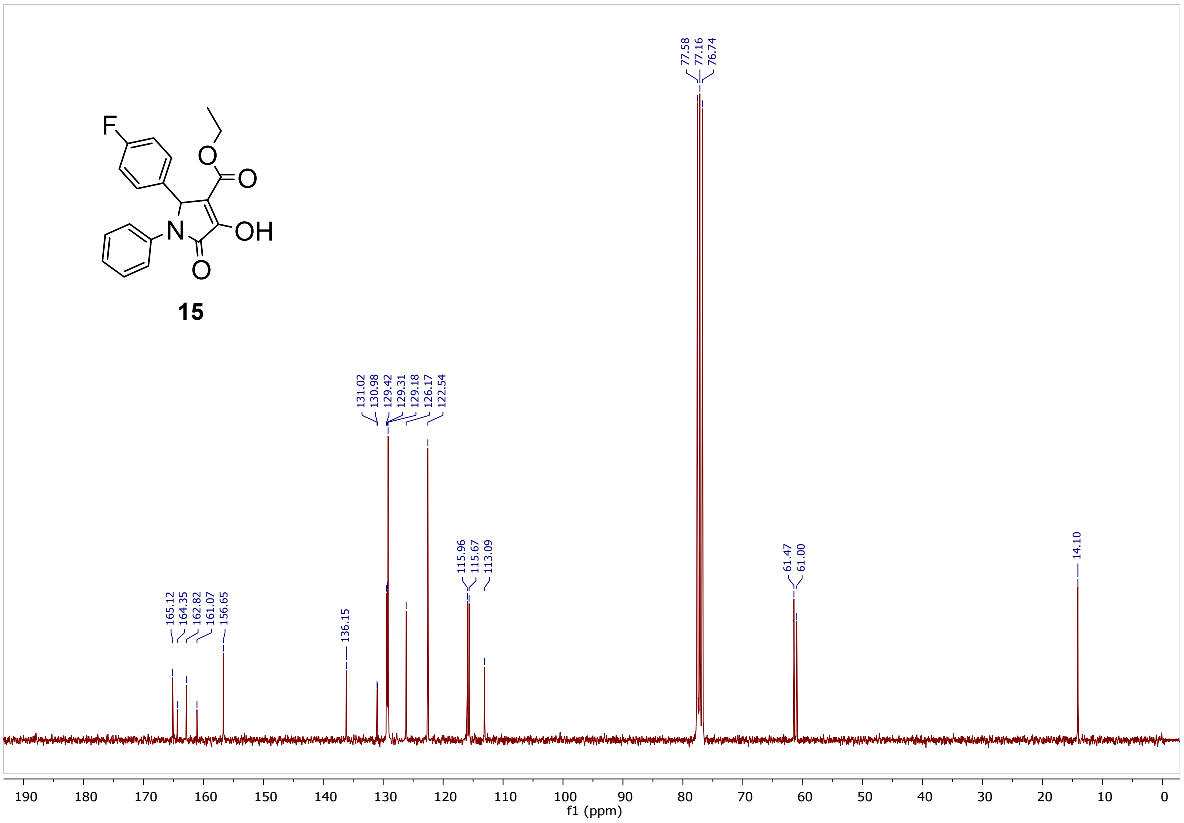

Figure S44. ^{13}C NMR spectrum of compound **15** at 75 MHz in $CDCl_3$.

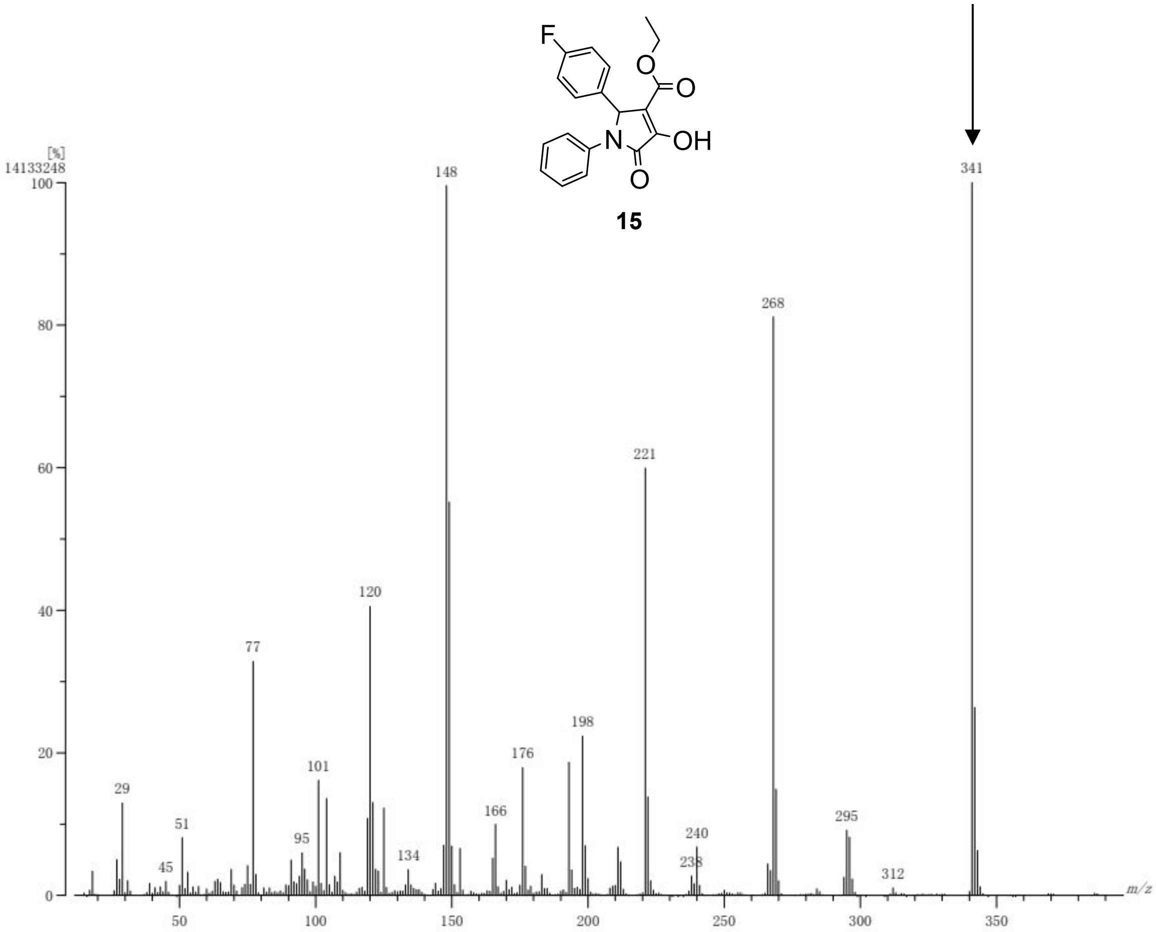

Figure S45. Electron impact mass spectra of compound **15**.

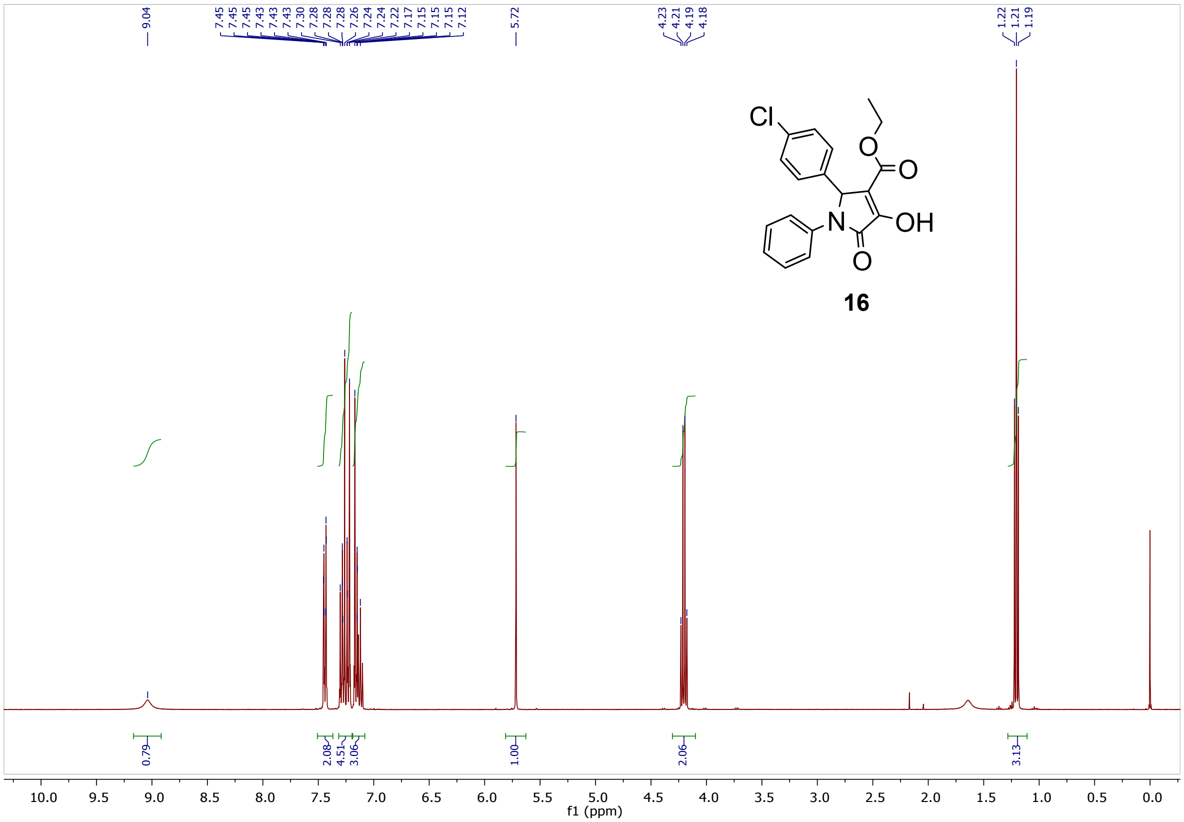

Figure S46. ^{1}H NMR spectrum of compound **16** at 300 MHz in $CDCl_3$.

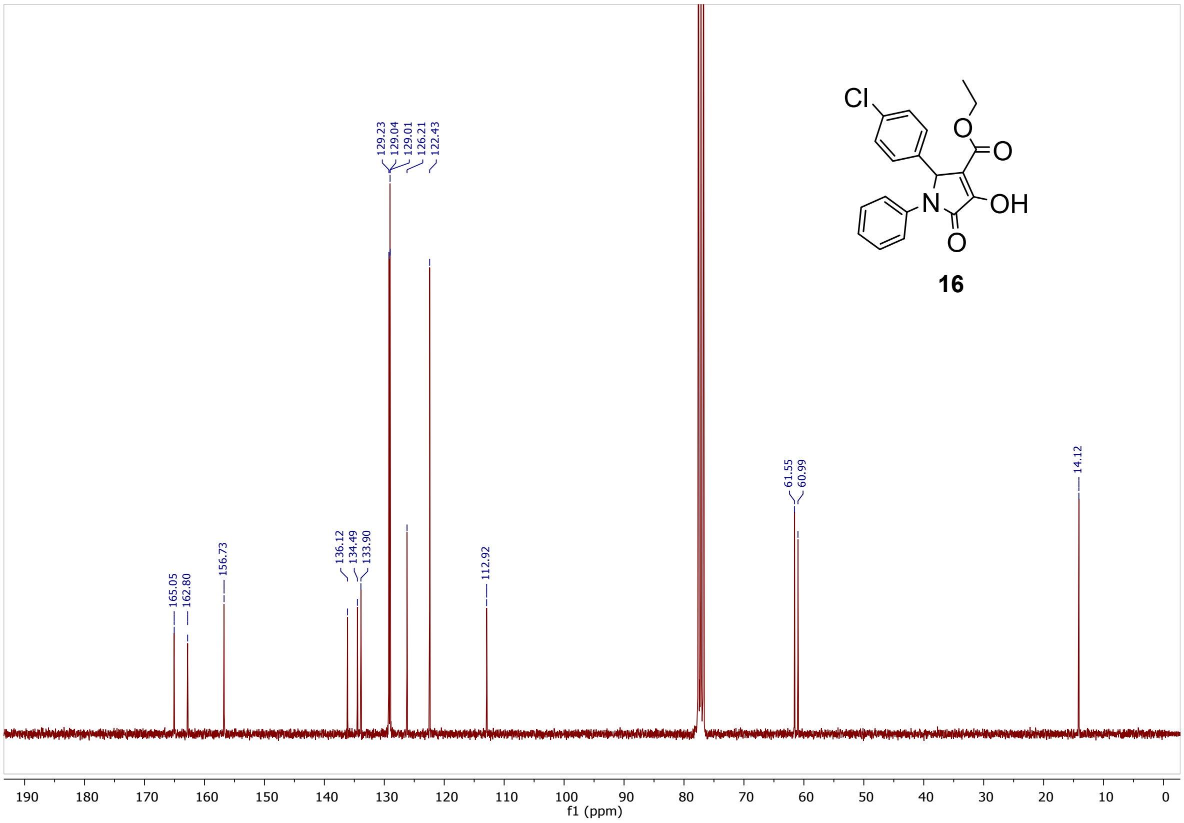

Figure S47. ^{13}C NMR spectrum of compound **16** at 75 MHz in $CDCl_3$.

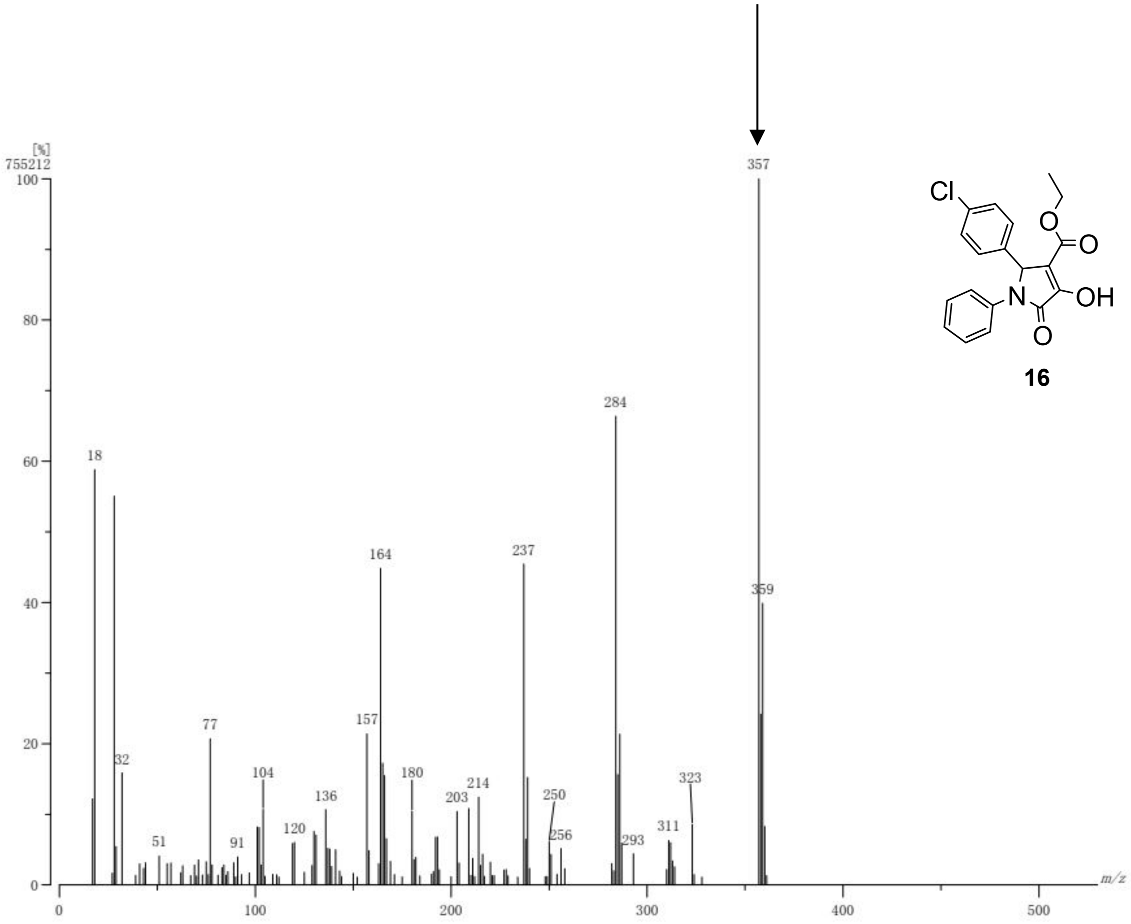

Figure S48. Electron impact mass spectra of compound **16**.

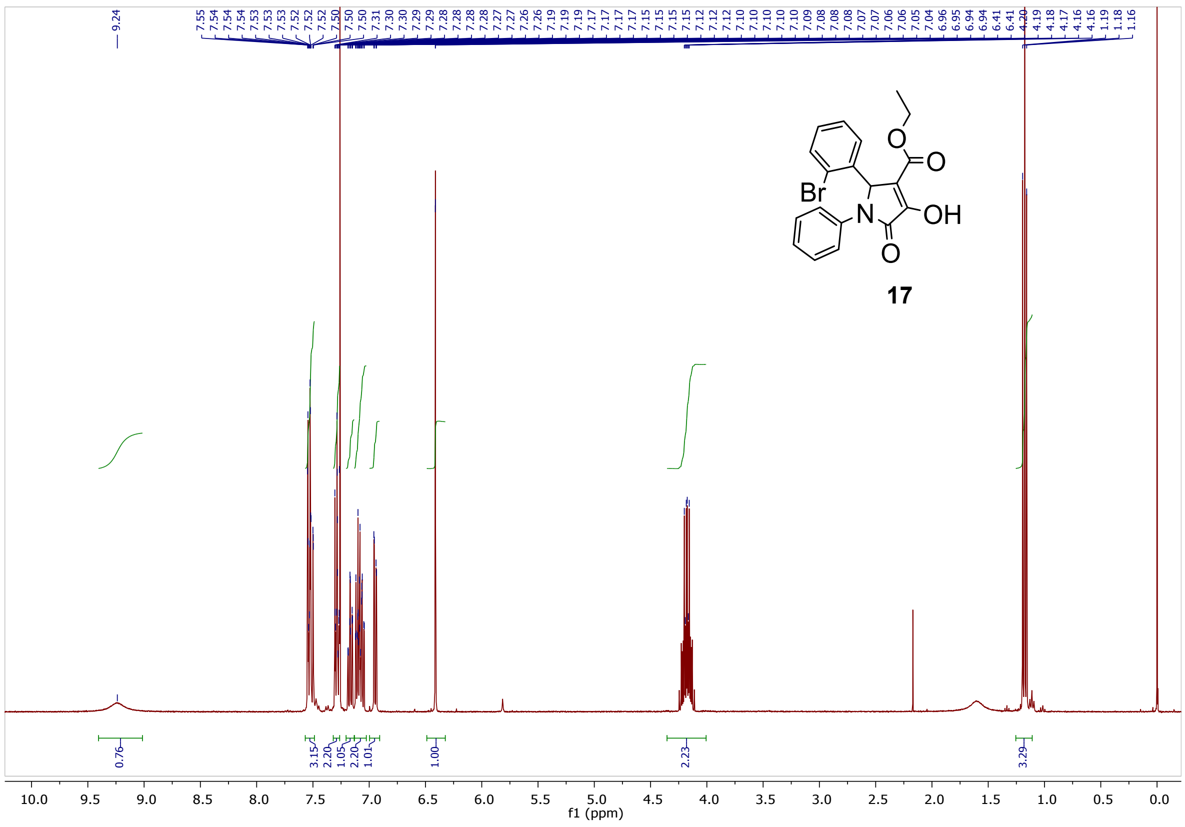

Figure S49. ^{1}H NMR spectrum of compound **17** at 300 MHz in $CDCl_3$.

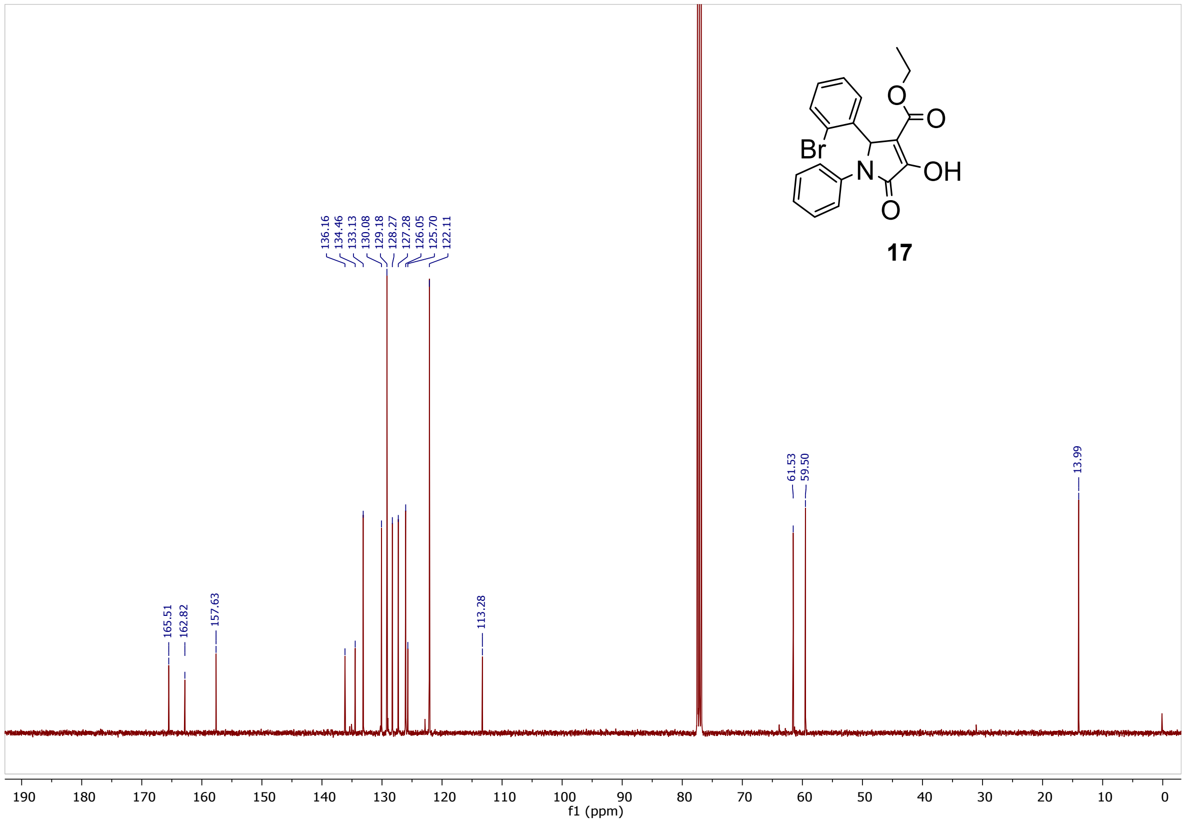

Figure S50. ^{13}C NMR spectrum of compound **17** at 75 MHz in $CDCl_3$.

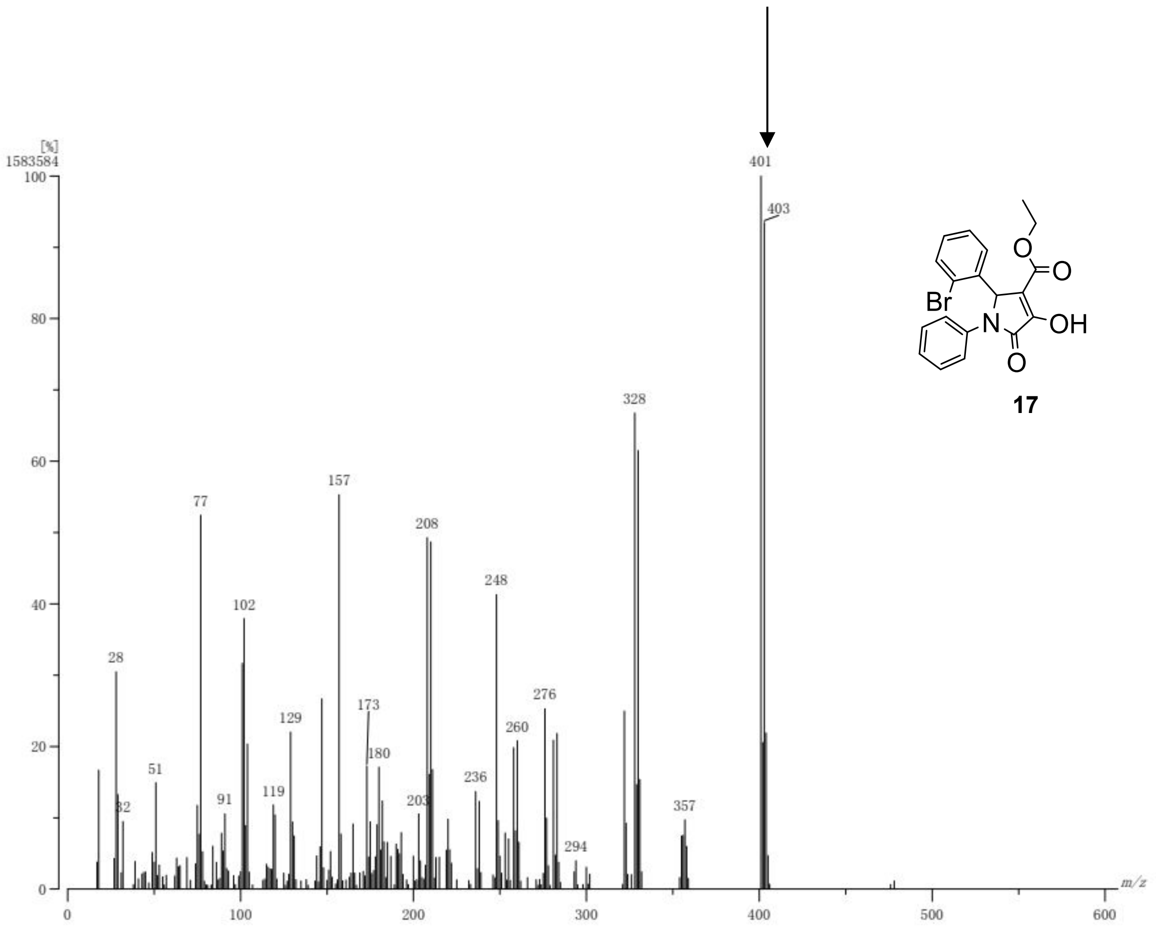

Figure S51. Electron impact mass spectra of compound **17**.

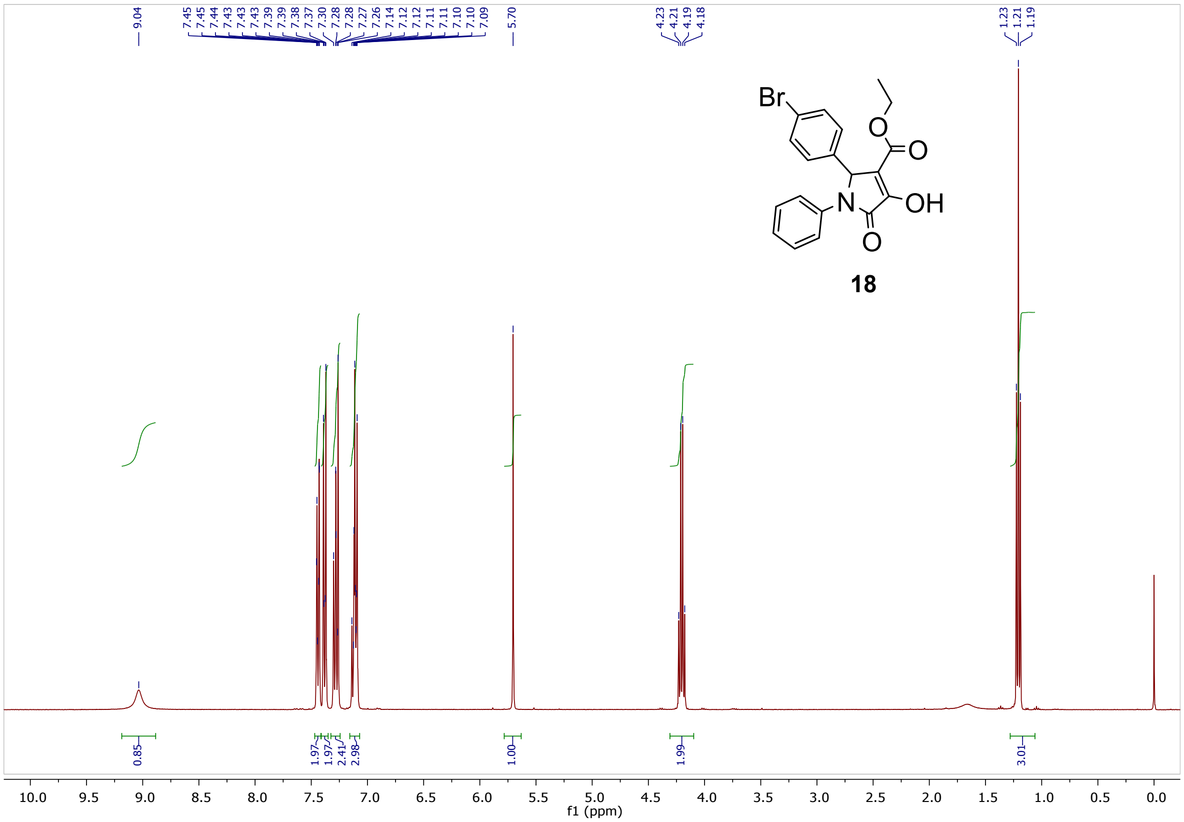

Figure S52. ^{1}H NMR spectrum of compound **18** at 400 MHz in $CDCl_3$.

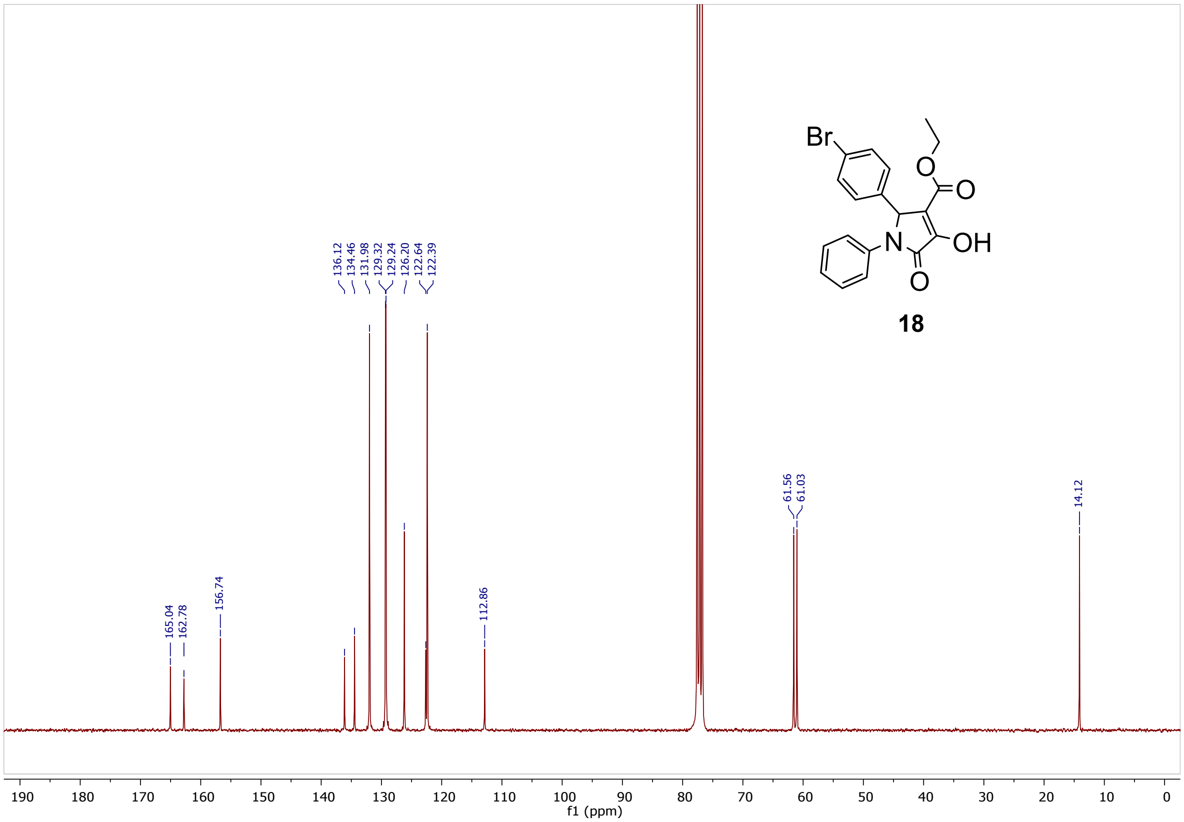

Figure S53. ^{13}C NMR spectrum of compound **18** at 100 MHz in $CDCl_3$.

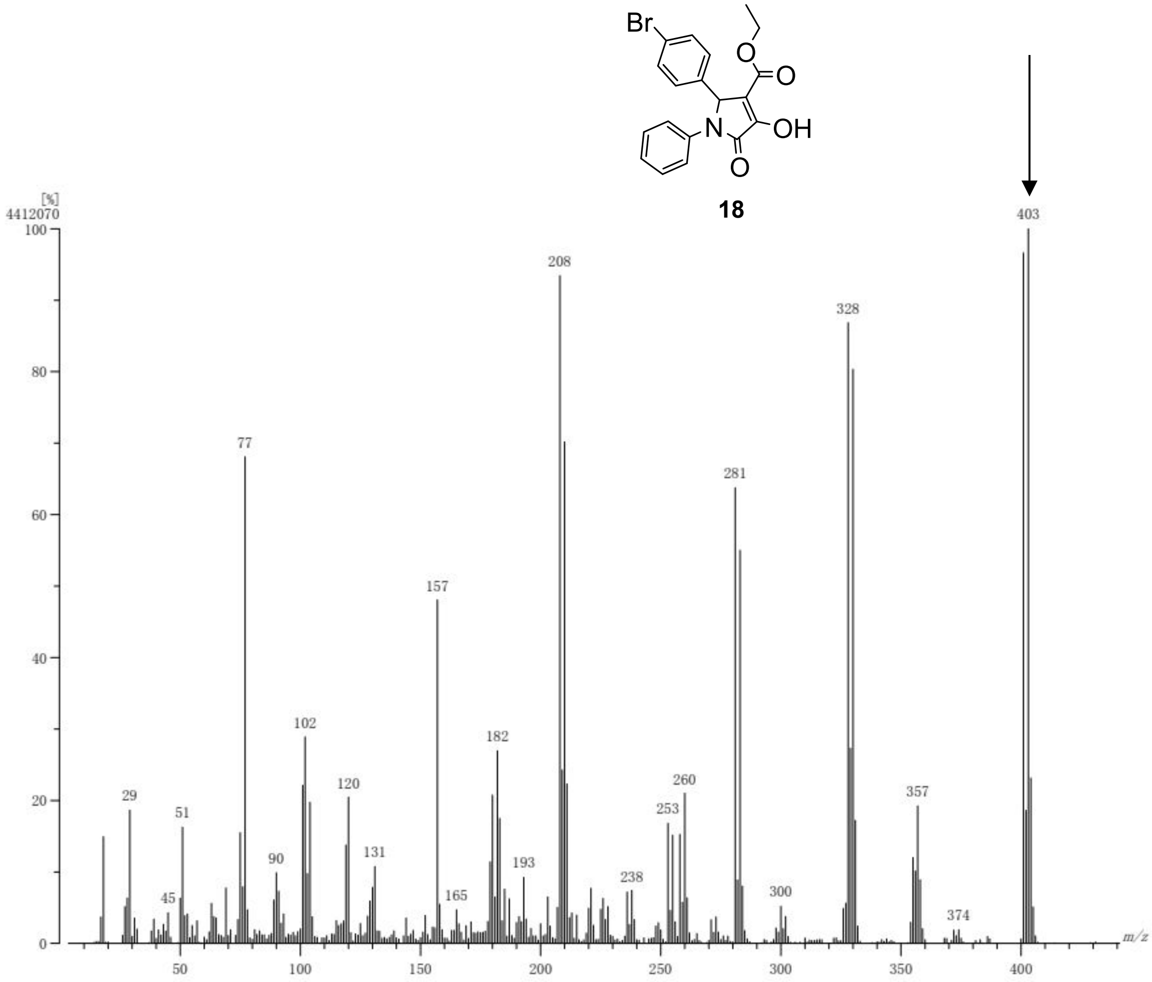

Figure S54. Electron impact mass spectra of compound **18**.

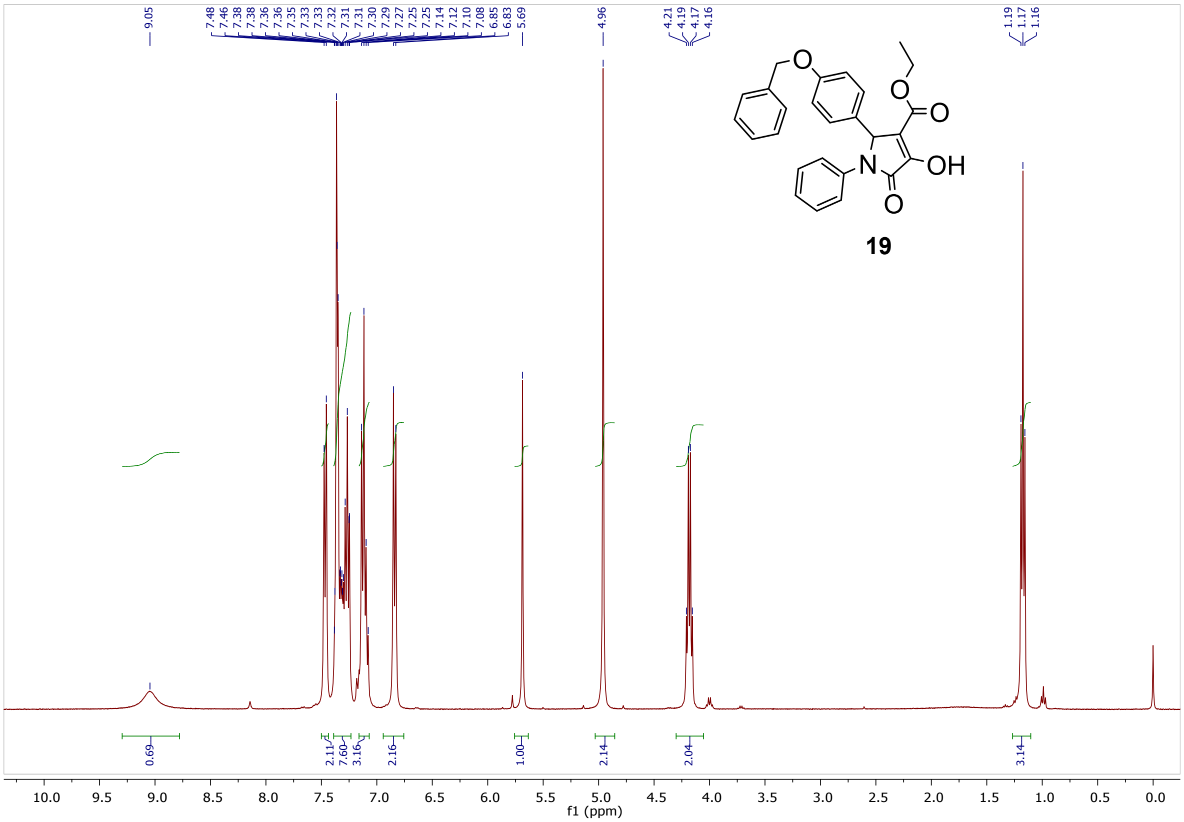

Figure S55. ^{1}H NMR spectrum of compound **19** at 400 MHz in $CDCl_3$.

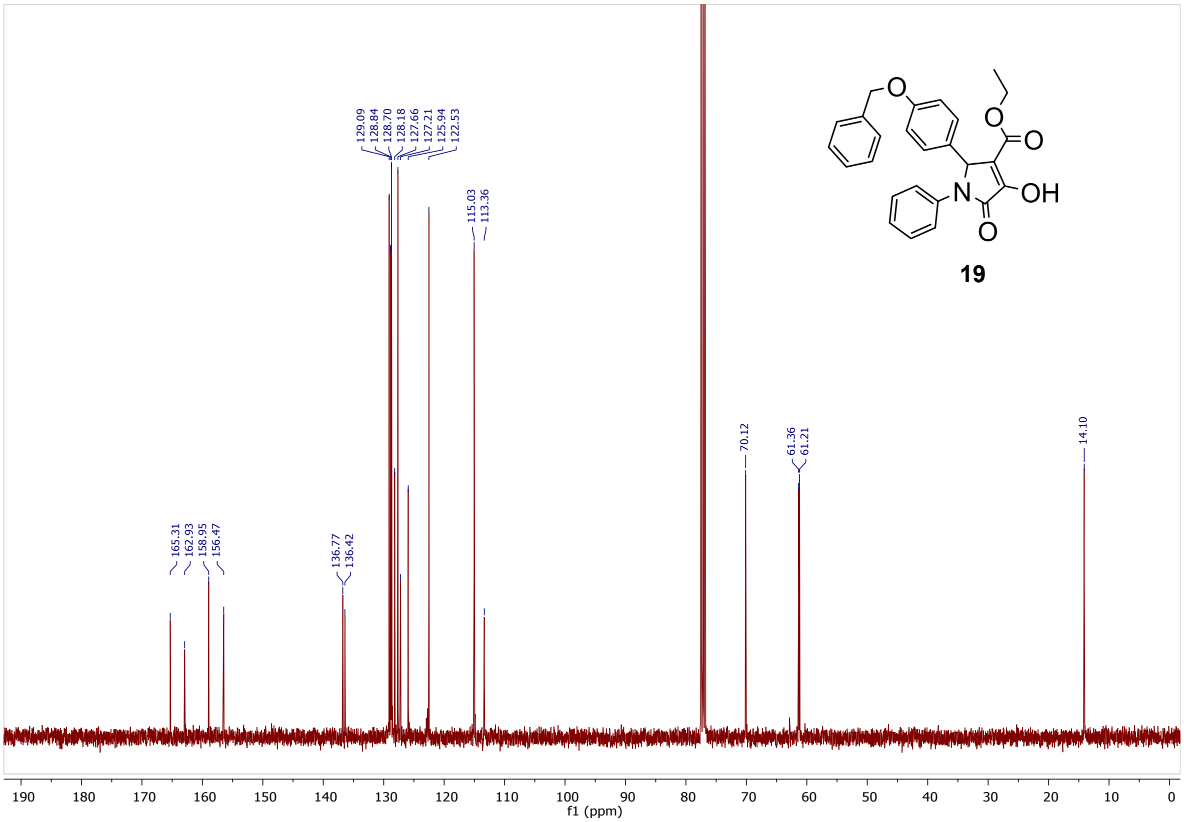

Figure S56. ^{13}C NMR spectrum of compound **19** at 100 MHz in $CDCl_3$.

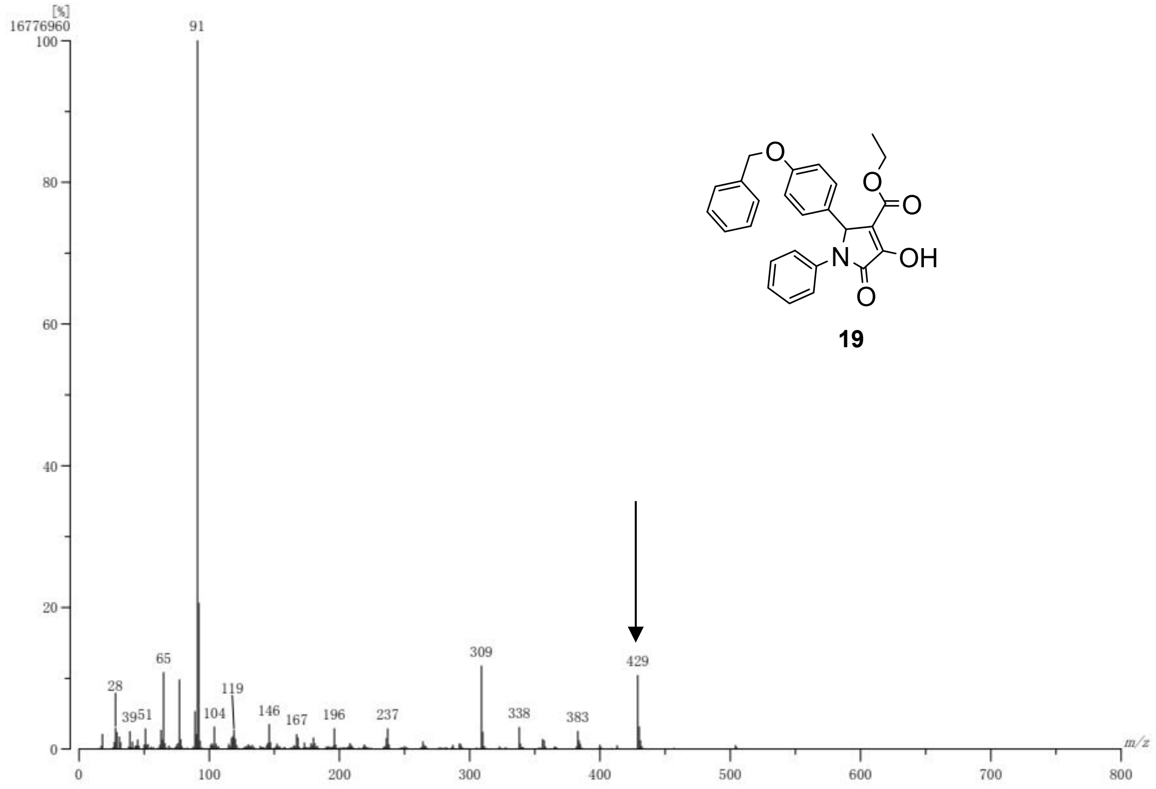

Figure S57. Electron impact mass spectra of compound **19**.

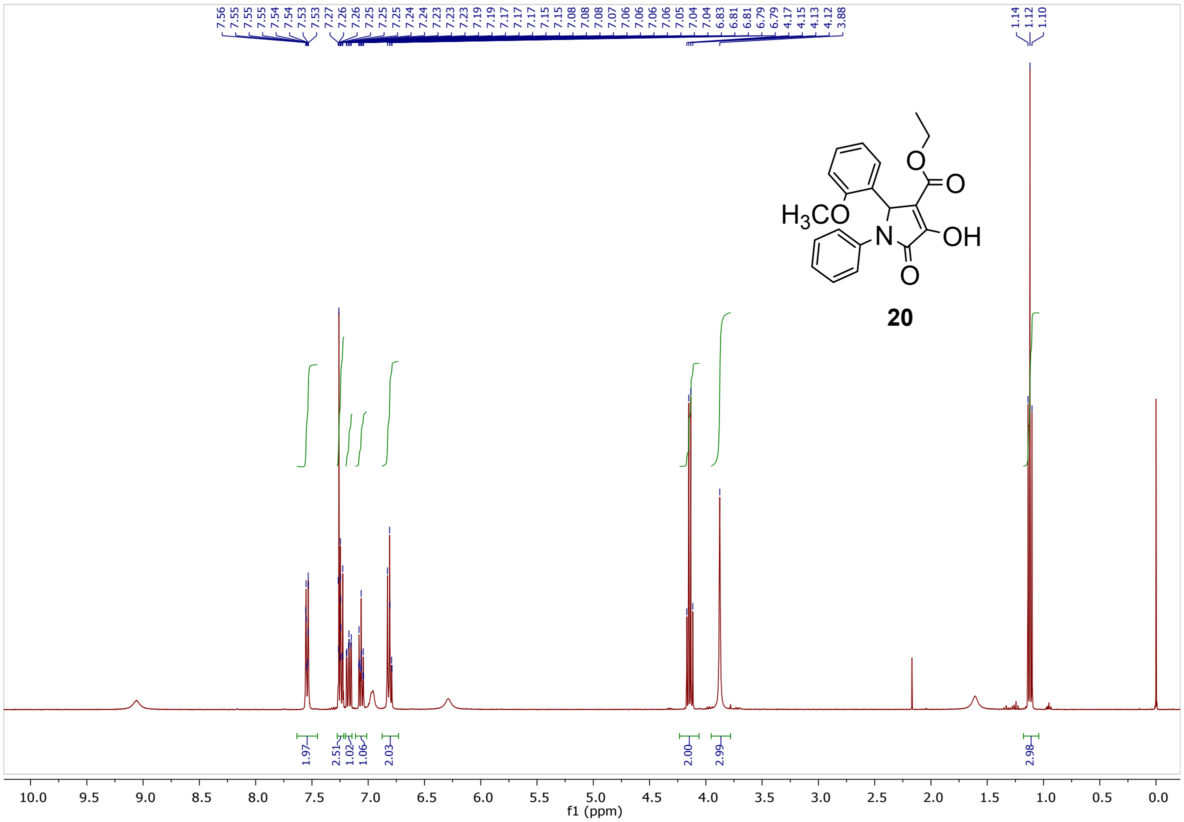

Figure S58. ^{1}H NMR spectrum of compound **20** at 300 MHz in $CDCl_3$.

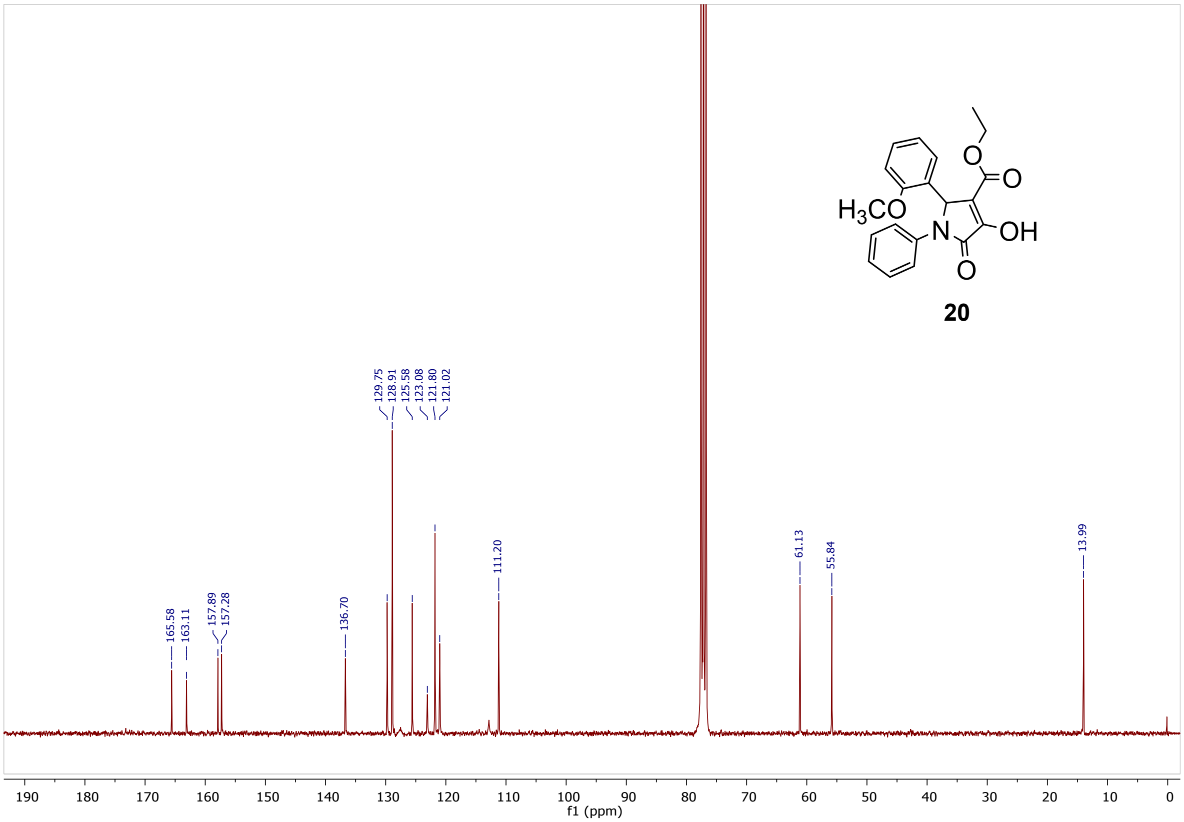

Figure S59. ^{13}C NMR spectrum of compound **20** at 75 MHz in $CDCl_3$.

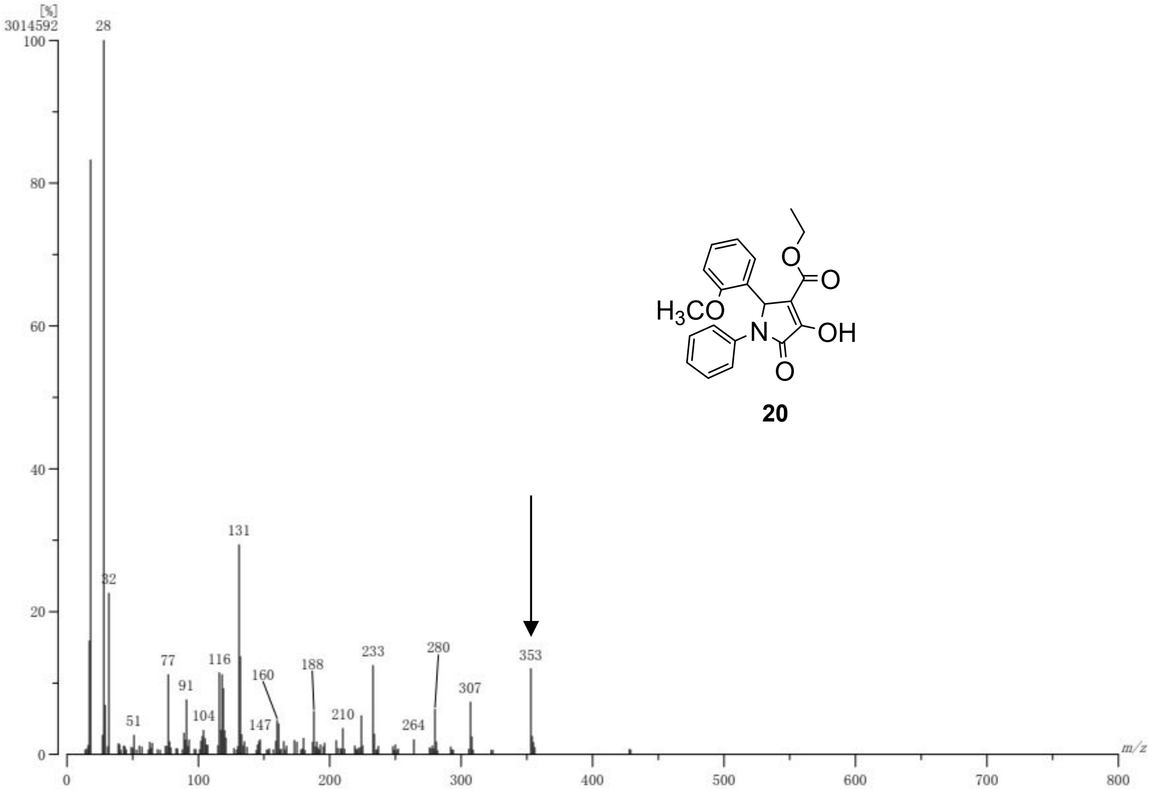

Figure S60. Electron impact mass spectra of compound **20**.

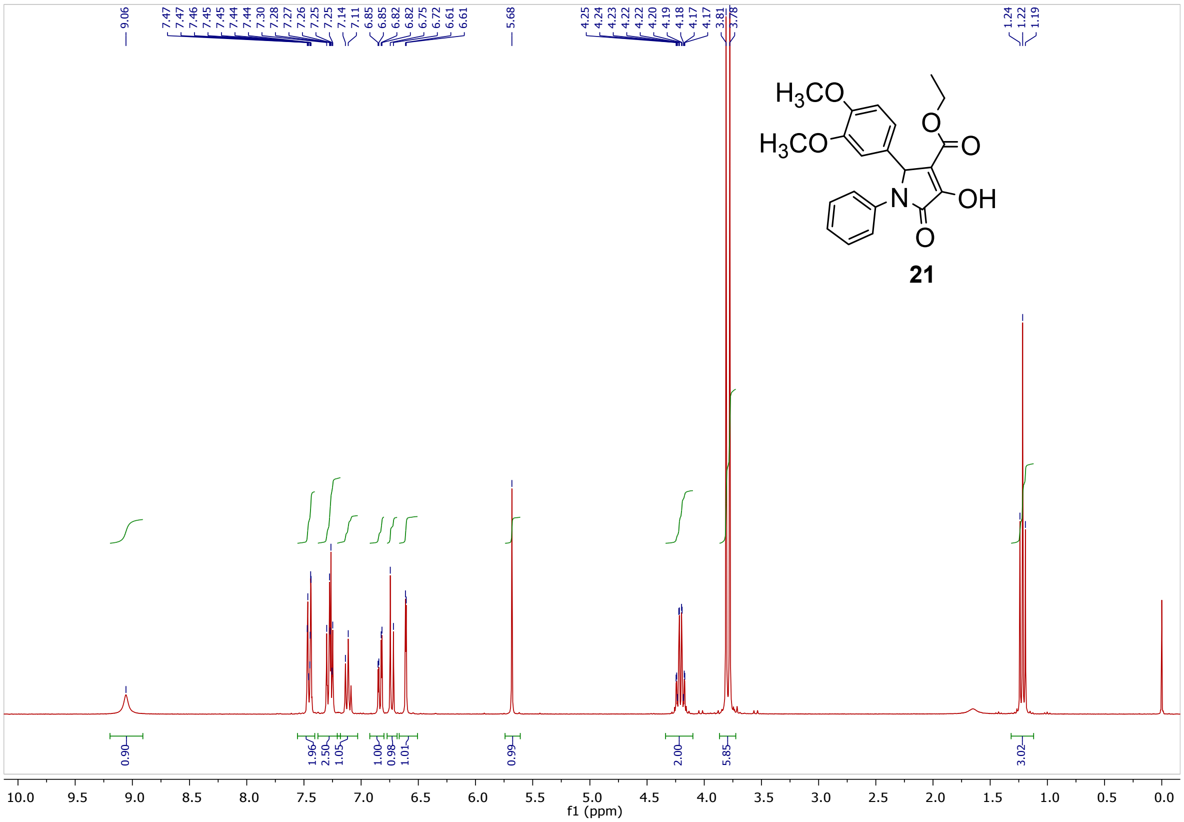

Figure S61. ^{1}H NMR spectrum of compound **21** at 300 MHz in $CDCl_3$.

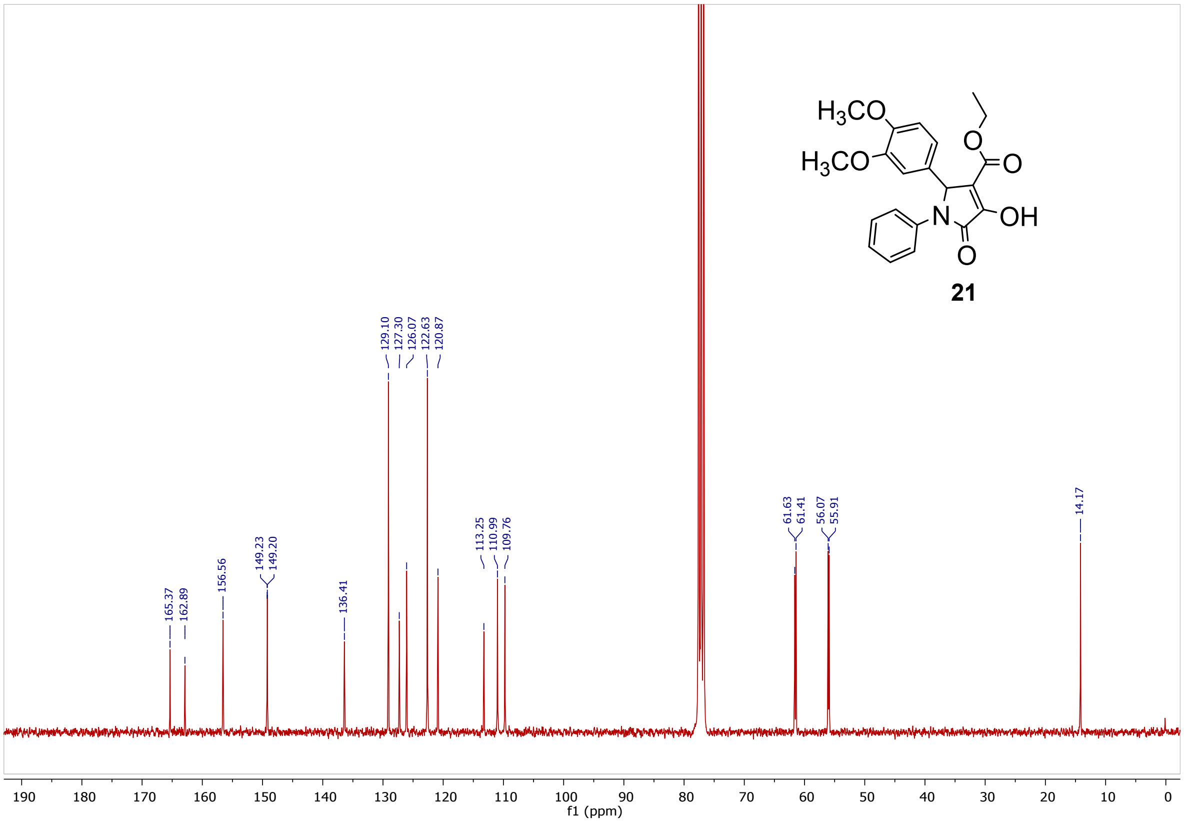

Figure S62. ^{13}C NMR spectrum of compound **21** at 75 MHz in $CDCl_3$.

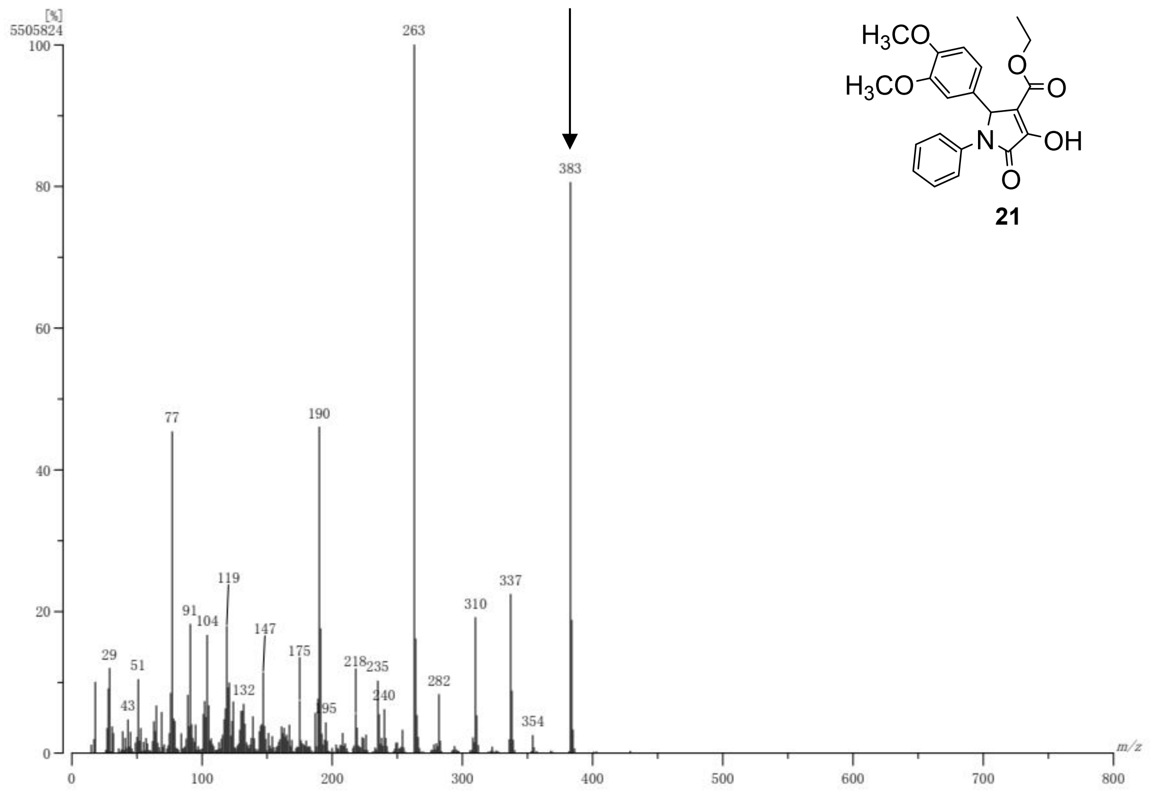

21

Figure S63. Electron impact mass spectra of compound **21**.

Single crystal X ray parameters of compound 2, 9, 12 and 20

Table S2. Single crystal X ray parameters of compound **2**.

**Empirical formula**	$C_{19}H_{16}FNO_4$	
**Formula weight**	341.33	
**Temperature**	100(2) K	
**Wavelength**	0.71073 Å	
**Crystal system**	Triclinic	
**Space group**	P-1	
**Unit cell dimensions**	a = 7.8323(3) Å	α = 79.1160(10)°.
	b = 9.9053(3) Å	β= 79.3110(10)°.
	c = 10.7901(4) Å	γ = 82.0930(10)°.
**Volume**	803.21(5) Å3	
**Z**	2	
**Density (calculated)**	1.411 Mg/m3	
**Absorption coefficient**	0.107 mm-1	
**F(000)**	356	
**Crystal size**	0.567 x 0.486 x 0.341 mm3	
**Theta range for data collection**	1.948 to 27.445°.	
**Index ranges**	-10<=h<=10, -12<=k<=12, -13<=l<=13	
**Reflections collected**	35214	
**Independent reflections**	3656 [R(int) = 0.0186]	
**Completeness to theta = 25.242°**	99.8 %	
**Absorption correction**	None	
**Refinement method**	Full-matrix least-squares on F2	
**Data / restraints / parameters**	3656 / 1 / 230	
**Goodness-of-fit on F^2**	1.043	
**Final R indices [I>2sigma(I)]**	R1 = 0.0339, wR2 = 0.0911	
**R indices (all data)**	R1 = 0.0354, wR2 = 0.0926	
**Extinction coefficient**	n/a	
**Largest diff. peak and hole**	0.353 and -0.221 e.Å^{-3}	

Table S3. Single crystal X ray parameters of compound **9**.

**Empirical formula**	$C_{21}H_{15}F_6NO_4$	
**Formula weight**	459.34	
**Temperature**	100(2) K	
**Wavelength**	0.71073 Å	
**Crystal system**	Triclinic	
**Space group**	P-1	
**Unit cell dimensions**	a = 8.9217(4) Å	α= 88.8898(8)°.
	b = 9.0618(4) Å	β= 87.1363(9)°.
	c = 11.8914(5) Å	γ = 82.0102(9)°.
**Volume**	950.78(7) $Å^3$	
**Z**	2	
**Density (calculated)**	1.604 Mg/m^3	
**Absorption coefficient**	0.149 mm^{-1}	
**F(000)**	468	
**Crystal size**	0.483 x 0.423 x 0.398 mm^3	
**Theta range for data collection**	2.308 to 27.445°.	
**Index ranges**	-11<=h<=11, -11<=k<=11, -15<=l<=15	
**Reflections collected**	20984	
**Independent reflections**	4346 [R(int) = 0.0206]	
**Completeness to theta = 25.242°**	99.6 %	
**Absorption correction**	None	
**Refinement method**	Full-matrix least-squares on F^2	
**Data / restraints / parameters**	4346 / 67 / 321	
**Goodness-of-fit on F^2**	1.051	
**Final R indices [I>2sigma(I)]**	R1 = 0.0386, wR2 = 0.1013	
**R indices (all data)**	R1 = 0.0411, wR2 = 0.1034	
**Extinction coefficient**	n/a	
**Largest diff. peak and hole**	0.570 and -0.367 e.$Å^{-3}$	

Table S4. Single crystal X ray parameters of compound **12**.

**Empirical formula**	$C_{19}H_{15}Cl_2NO_4$	
**Formula weight**	392.22	
**Temperature**	100(2) K	
**Wavelength**	0.71073 Å	
**Crystal system**	Monoclinic	
**Space group**	C2/c	
**Unit cell dimensions**	a = 16.9510(8) Å	α= 90°.
	b = 10.1079(5) Å	β= 108.6789(8)°.
	c = 21.7669(10) Å	γ = 90°.
**Volume**	3533.1(3) $Å^3$	
**Z**	8	
**Density (calculated)**	1.475 Mg/m^3	
**Absorption coefficient**	0.393 mm^{-1}	
**F(000)**	1616	
**Crystal size**	0.569 x 0.539 x 0.442 mm^3	
**Theta range for data collection**	1.975 to 27.442°.	
**Index ranges**	-21<=h<=21, -13<=k<=13, -28<=l<=28	
**Reflections collected**	37907	
**Independent reflections**	4027 [R(int) = 0.0213]	
**Completeness to theta = 25.242°**	99.8 %	
**Absorption correction**	None	
**Refinement method**	Full-matrix least-squares on F^2	
**Data / restraints / parameters**	4027 / 1 / 239	
**Goodness-of-fit on F^2**	1.056	
**Final R indices [I>2sigma(I)]**	R1 = 0.0288, wR2 = 0.0766	
**R indices (all data)**	R1 = 0.0300, wR2 = 0.0775	
**Extinction coefficient**	n/a	
**Largest diff. peak and hole**	0.401 and -0.234 e.$Å^{-3}$	

Table S5. Single crystal X ray parameters of compound **20**.

**Empirical formula**	$C_{20}H_{19}NO_5$	
**Formula weight**	353.36	
**Temperature**	100(2) K	
**Wavelength**	0.71073 Å	
**Crystal system**	Triclinic	
**Space group**	P-1	
**Unit cell dimensions**	a = 9.2491(5) Å	α= 69.7774(9)°.
	b = 9.3574(4) Å	β= 79.6451(10)°.
	c = 11.6013(6) Å	γ = 64.0619(9)°.
**Volume**	846.78(7) Å^3	
**Z**	2	
**Density (calculated)**	1.386 Mg/m^3	
**Absorption coefficient**	0.100 mm^{-1}	
**F(000)**	372	
**Crystal size**	0.554 x 0.507 x 0.471 mm^3	
**Theta range for data collection**	2.539 to 27.445°.	
**Index ranges**	-11<=h<=11, -12<=k<=12, -15<=l<=15	
**Reflections collected**	18704	
**Independent reflections**	3856 [R(int) = 0.0212]	
**Completeness to theta = 25.242°**	99.8 %	
**Absorption correction**	None	
**Refinement method**	Full-matrix least-squares on F^2	
**Data / restraints / parameters**	3856 / 1 / 240	
**Goodness-of-fit on F^2**	1.042	
**Final R indices [I>2sigma(I)]**	R1 = 0.0342, wR2 = 0.0865	
**R indices (all data)**	R1 = 0.0371, wR2 = 0.0886	
**Extinction coefficient**	n/a	
**Largest diff. peak and hole**	0.364 and -0.228 e.Å^{-3}	

Water influence experiments

Reaction using diethyl oxalacetate: A mixture of sodium diethyl oxaloacetate (1.0 equiv), aniline (1.0 equiv), and benzaldehyde (1.0 equiv) in ethanol was heated at 60 °C until completion, as monitored by TLC (Squeme S1). After cooling to room temperature, the reaction mixture was poured into ice–water and acidified with hydrochloric acid to pH 4. The resulting precipitate was collected by filtration, then washed sequentially with water and cold ethanol to remove residual reactants. The product was obtained as a white solid with sufficient purity for spectroscopic and spectrometric analysis.

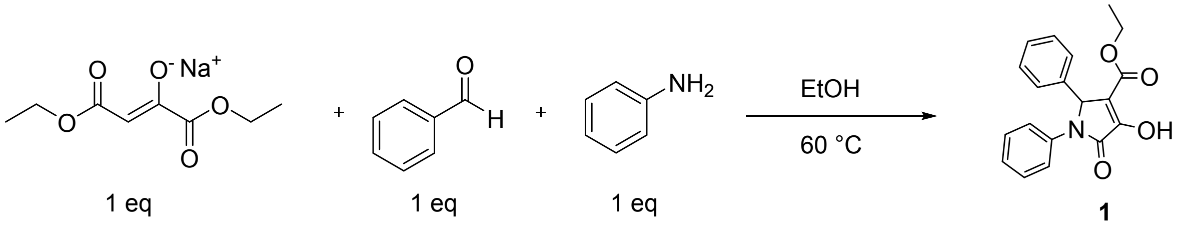

Scheme 1. Reaction using the sodium salt of diethyl oxaloacetate as a precursor for the synthesis of pyrrolidone **1.**

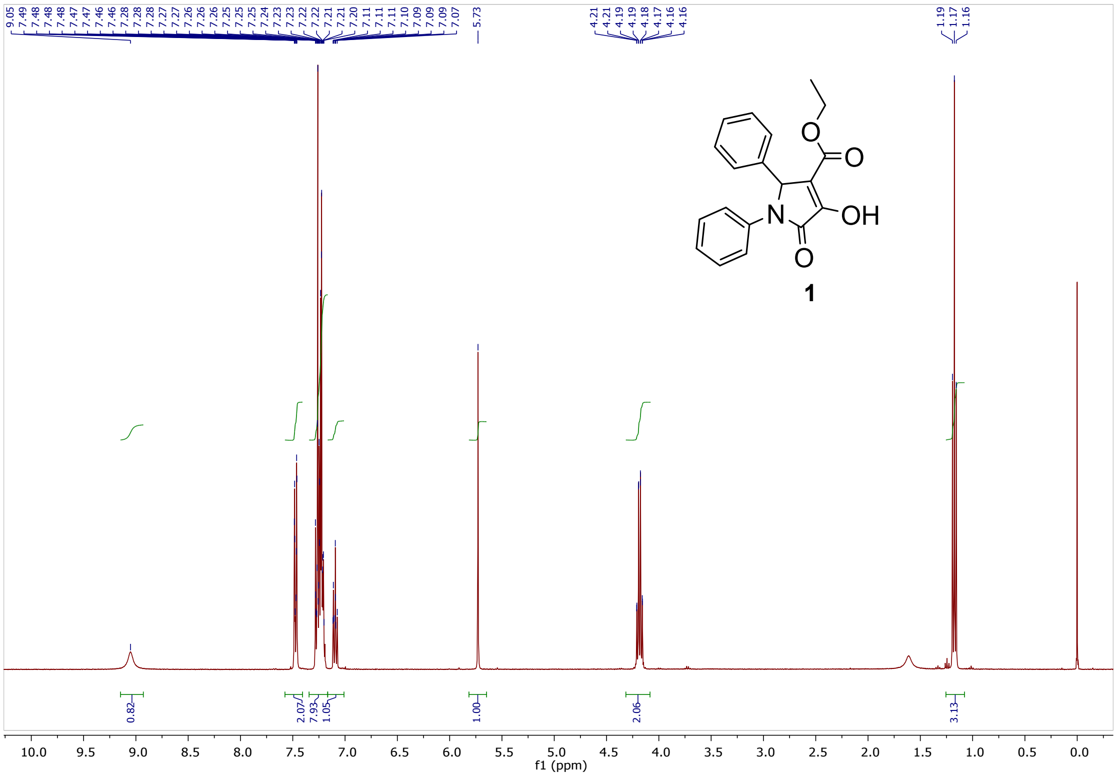

Figure S64. ^{1}H NMR spectrum (400 MHz, $CDCl_3$) of product obtained from reaction with sodium salt of diethyl oxaloacetate (pyrrolidinone **1**).

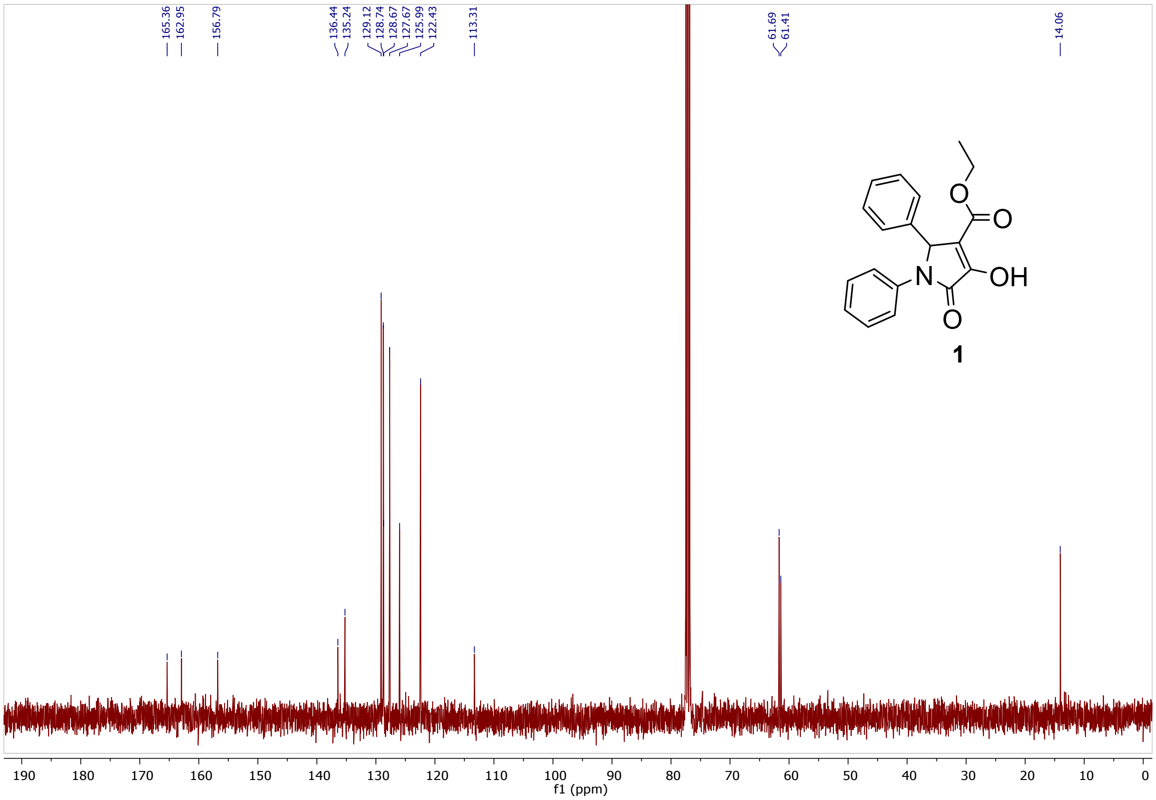

Figure S65. ^{13}C NMR spectrum (100 MHz, $CDCl_3$) of product obtained from reaction with sodium salt of diethyl oxaloacetate (pyrrolidinone **1**).

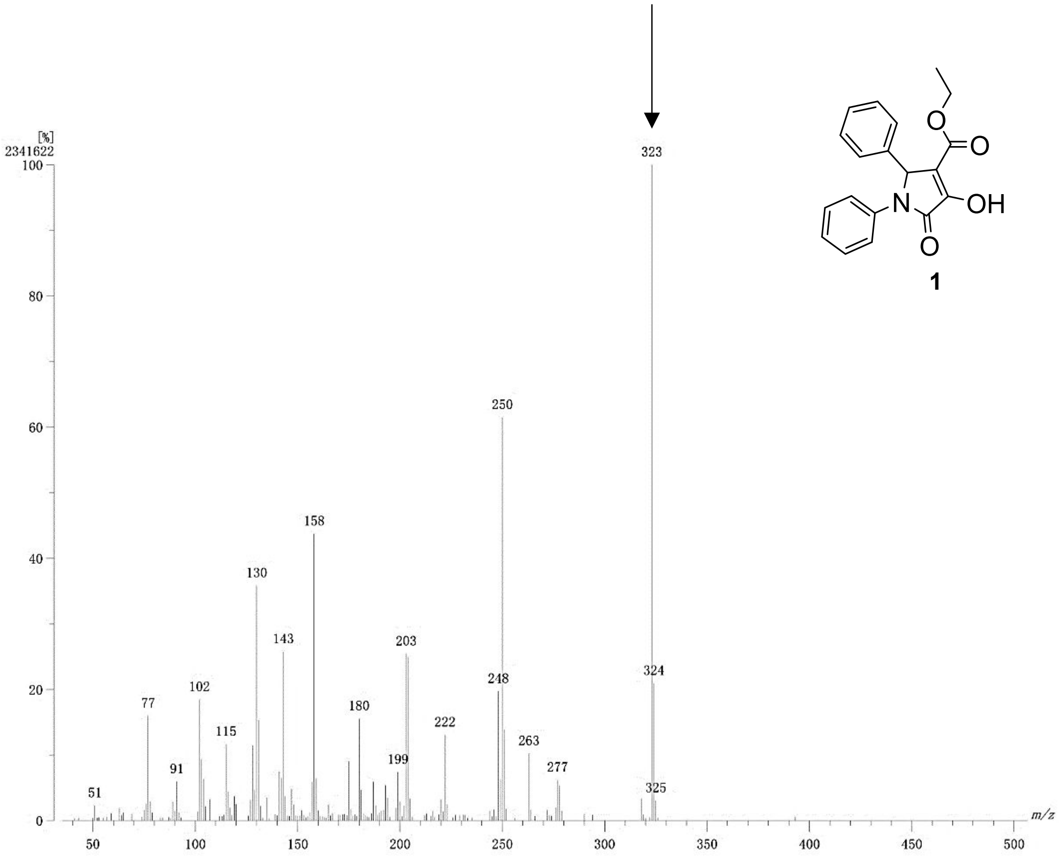

Figure S66. Electron impact mass spectra of product obtained from reaction with sodium salt of diethyl oxaloacetate (pyrrolidinone **1**).

Reaction using molecular sieves: A mixture of diethyl acetylenedicarboxylate (1.0 equiv), aniline (1.0 equiv), benzaldehyde (1.0 equiv), and 3 Å molecular sieves was stirred in solvent and catalyst-free conditions at 60 °C for 3 h. Thin-layer chromatography (TLC) analysis indicated no formation of pyrrolidone-type products under these conditions. However, a more precise analysis was carried out using High Performance Liquid Chromatography.

HPLC Analysis.

Both a reference sample of the pyrrolidone product (**A**) and the crude from the reaction using molecular sieves (**B**) were analyzed using an Agilent 1260 Infinity II UV–Vis Diode Array HPLC system (Figure S67). Separation was performed on a NUCLEODUR C8 Gravity column (150 × 4.6 mm, 5 μm particle size). Samples were prepared in acetonitrile and injected under gradient elution conditions: starting with a mobile phase of acetonitrile/water (25:75, v/v), followed by a switch to 100% acetonitrile after 30 min. The flow rate was maintained at 1.0 mL/min, and detection was carried out at 254 nm. The following chromatograms were obtained.

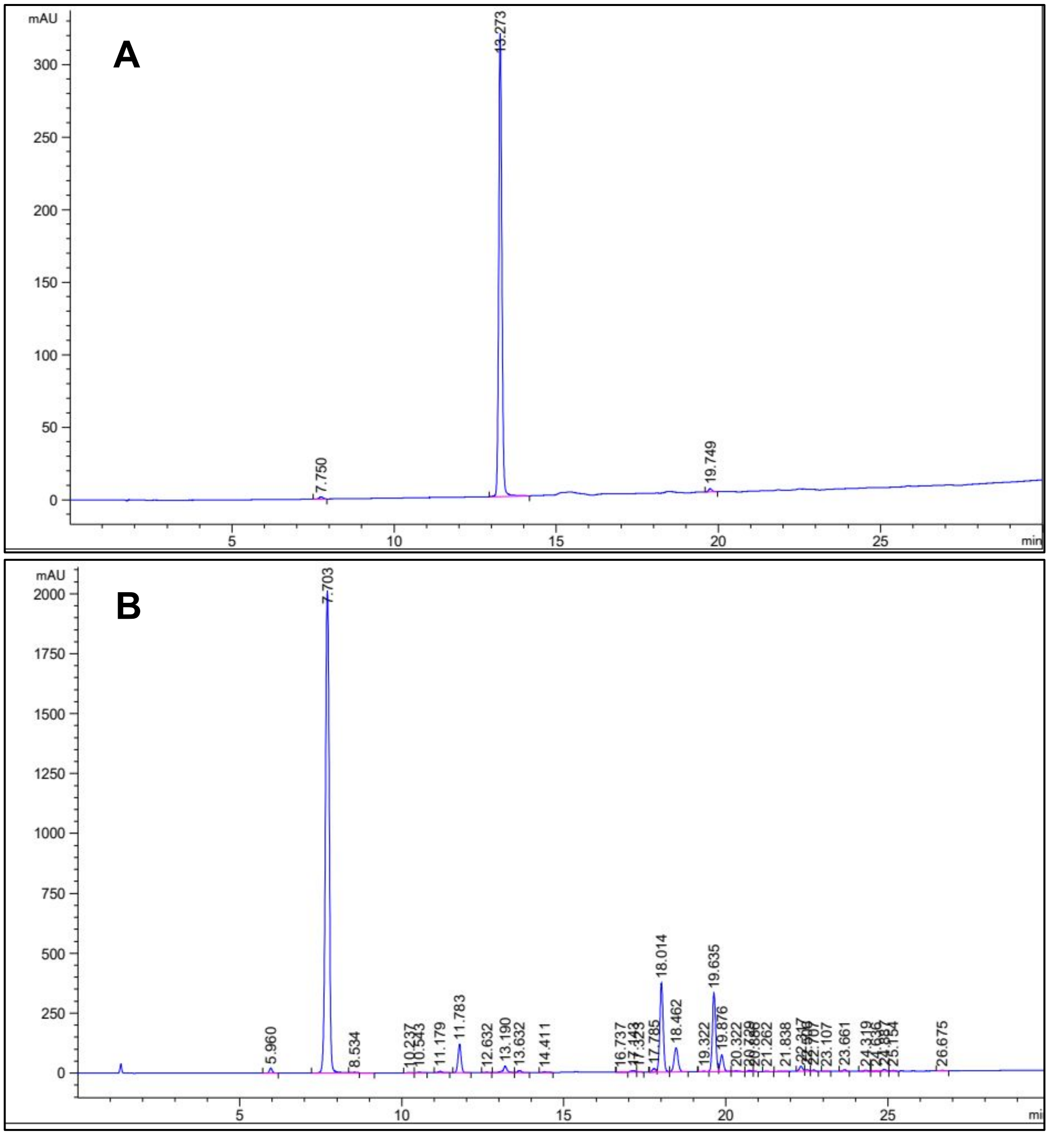

Figure S67. HPLC chromatograms: (**A**) pyrrolidone standard; (**B**) crude from the solvent- and catalyst-free reaction (no matching peak).

Enamine determination as byproduct

The pyrrolidone **1** was precipitated from the ethanolic mixture. Subsequently, an HPLC analysis was performed to confirm the presence of the enamine (a reaction byproduct) in the remaining mother liquors after precipitation. The analyzed samples are indicated in the chromatograms as follows: Reaction mother liquor (Black), enamine standard (Red) and Reaction mother liquor + enamine standard (Blue) (Figure S68).

Chromatographic profiles of the analyzed samples were obtained using a high-performance liquid chromatography system (HPLC, Waters e2695) equipped with a photodiode array detector (PDA, Waters 2998), recording absorbance in the range of 190–700 nm. Separation of the sample constituents was achieved on a reverse-phase C18 column (Phenomenex Gemini C18, 250 × 4.6 mm, 5 µm) using mixtures of water (acidified with 0.1% v/v formic acid) and acetonitrile (CH_3CN) as the mobile phase. The elution gradient consisted of an initial 20% CH_3CN, followed by a linear gradient from 20% to 100% CH_3CN over 30 min, and finally 5 min at 100% CH_3CN. Samples were dissolved in an HPLC-grade MeOH/dioxane mixture (8:2 v/v), and the injection volume was 10 µL.

Additionally, an NMR analysis was conducted by comparing with an enamine standard synthesized according to the specifications detailed in the experimental protocol.

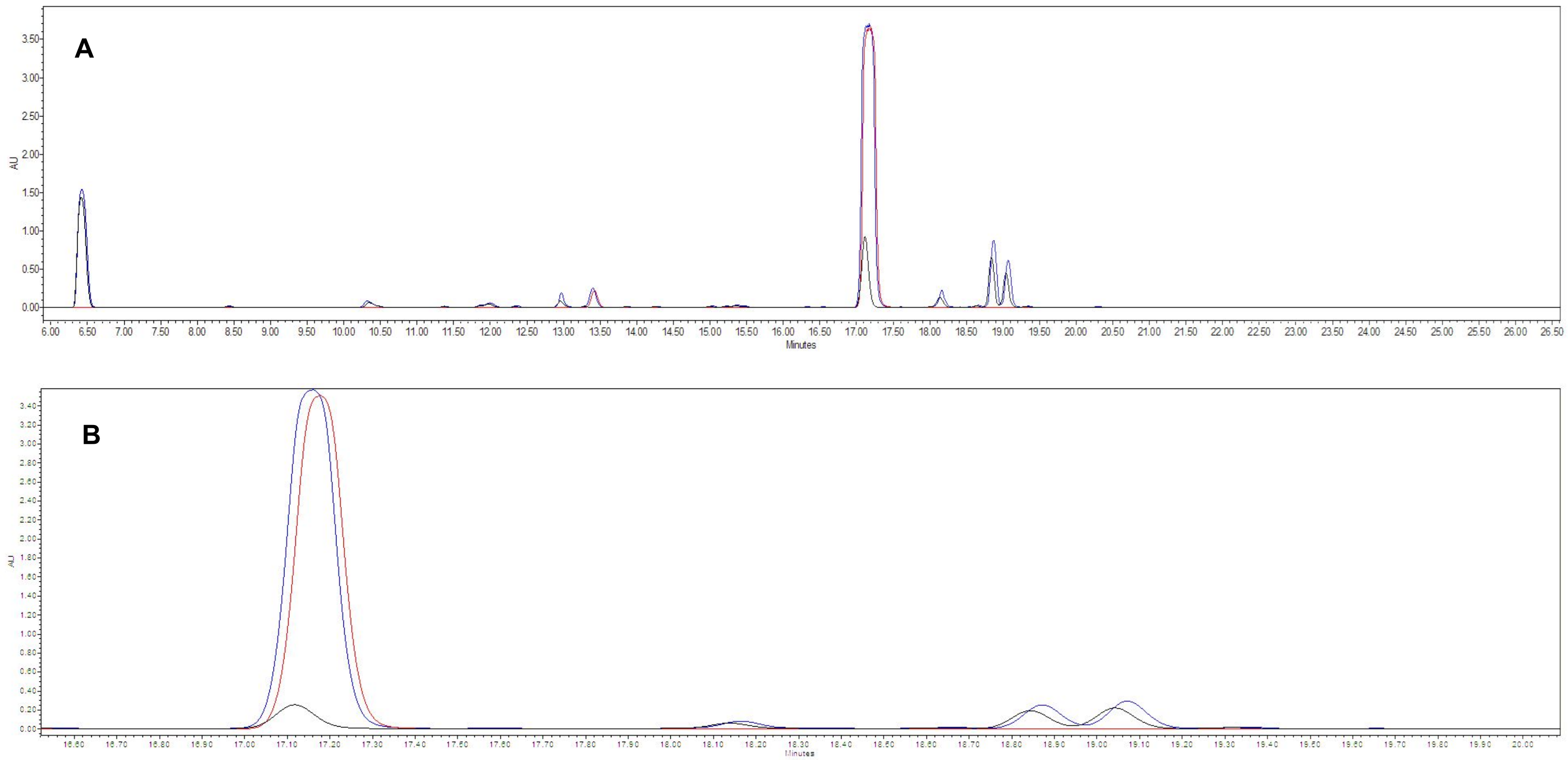

Figure S68. HPLC chromatograms: (**A**) Reaction mother liquor (Black), enamine standard (Red) and reaction mother liquor + enamine standard (Blue); (**B**) Enlarged view of the chromatogram (retention time 17–20 min).

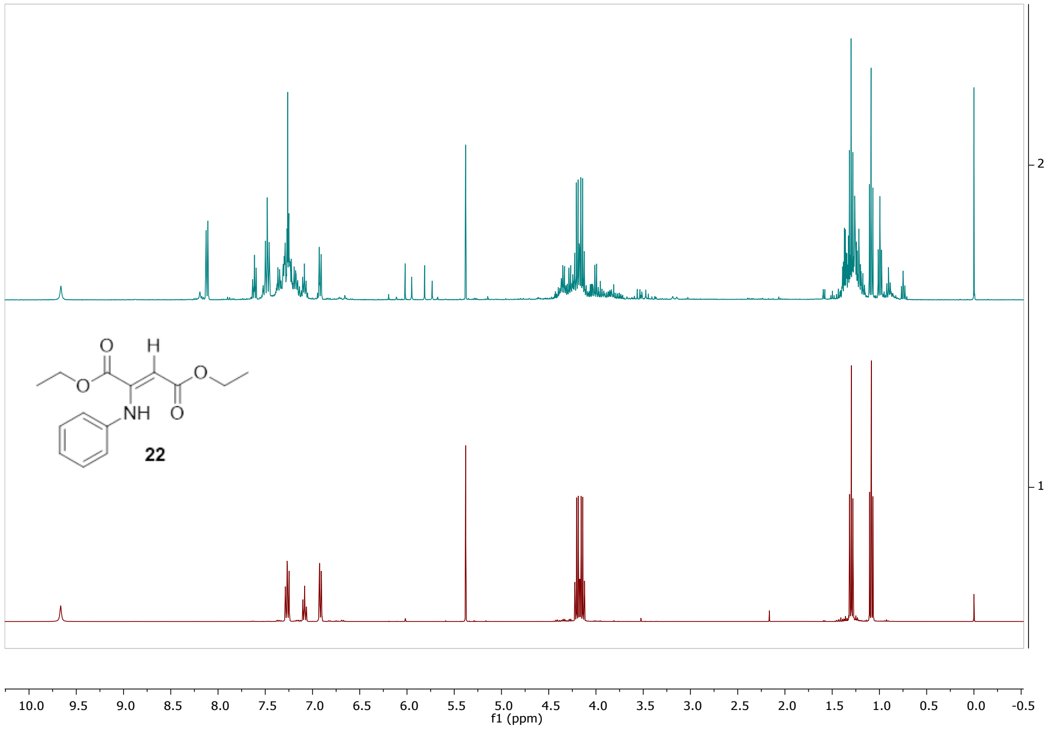

Figure S69. Comparison of ¹H NMR spectra (400 MHz, $CDCl_3$) of the reaction mother liquor (top) and the enamine standard **22** (bottom).

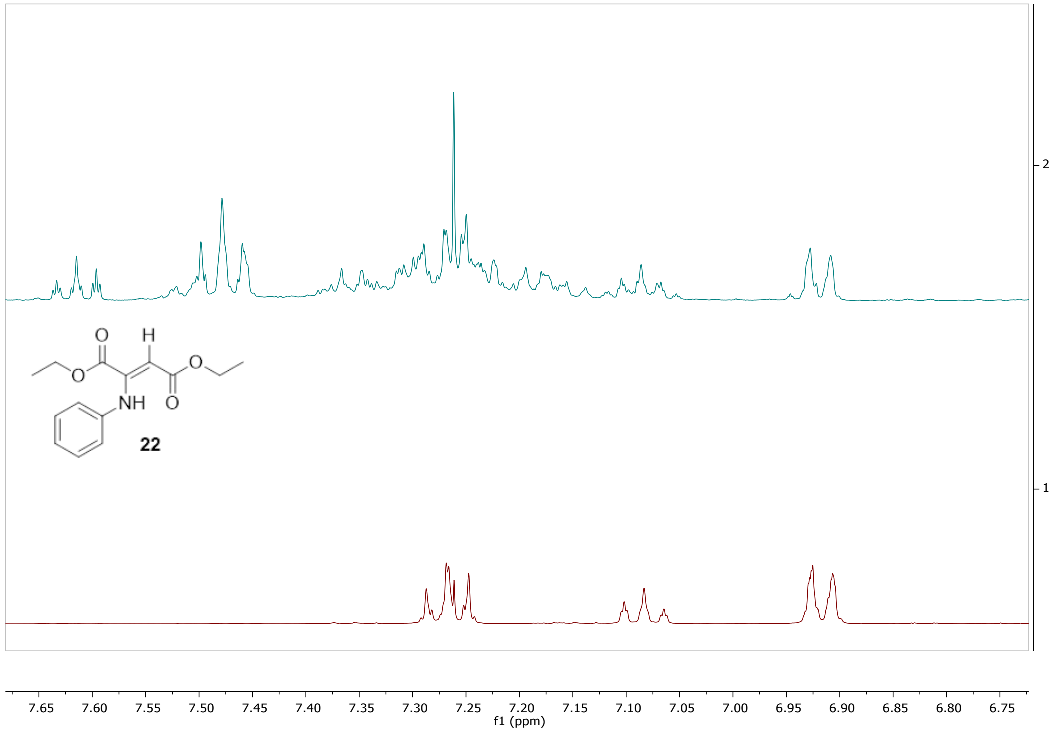

Figure S70. Comparison of ¹H NMR spectra (400 MHz, $CDCl_3$) of the reaction mother liquor (2) and the enamine standard (1), showing a zoomed-in view of the aromatic region.

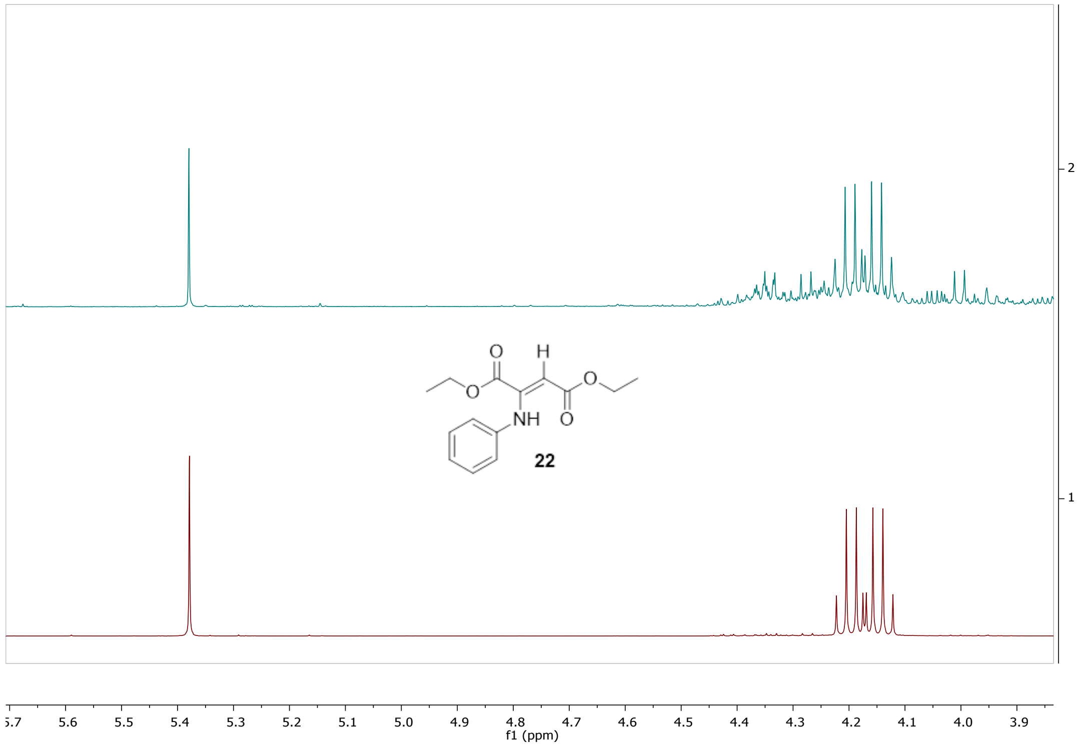

Figure S71. Comparison of ^{1}H NMR spectra (400 MHz, $CDCl_3$) for the reaction mother liquor (2) and the enamine standard (1), showing a zoomed-in view of the vynilic and heteroatom region.

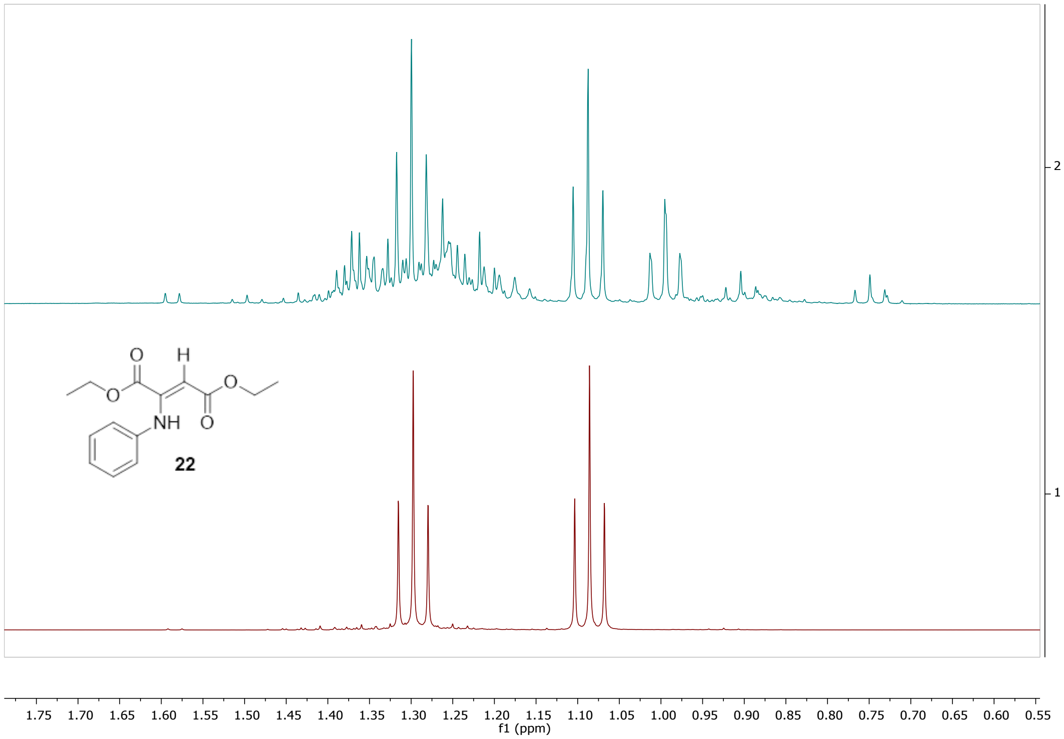

Figure S72. Comparison of ^{1}H NMR spectra (400 MHz, $CDCl_3$) for the reaction mother liquor (2) and the enamine standard (1), showing a zoomed-in view of the aliphatic region**.**

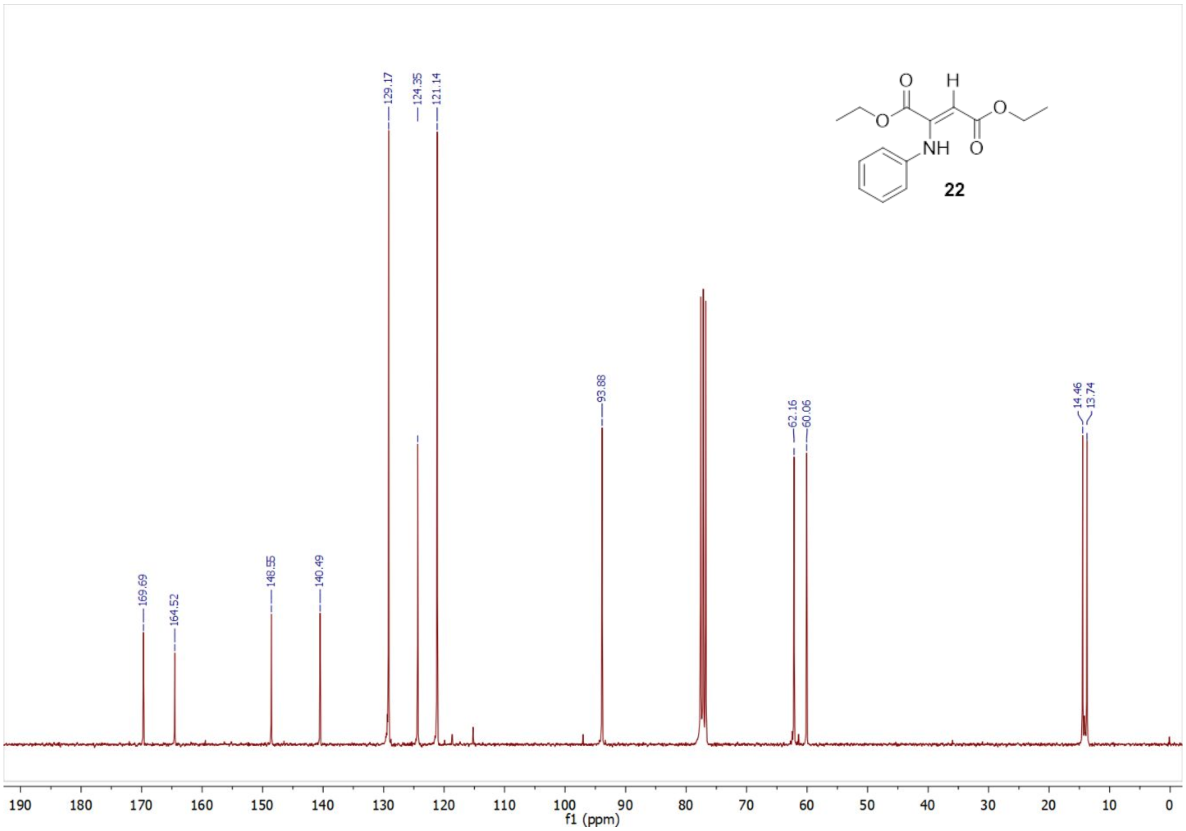

Figure S73. ^{13}C NMR spectrum of compound **22** at 75 MHz in $CDCl_3$.

Partial charge analysis of diethyl oxaloacetate (M06-2X-D3/def2-TZVP)

Table S6. Partial atomic charges (in e) of diethyl oxaloacetate calculated at the M06-2X-D3/def2-TZVP level at 298.15 K (Gaussian 16). Optimized geometries were confirmed as true minima (no imaginary frequencies).

Entry	Atom type	Mulliken charges	Mulliken charges with hydrogens summed into heavy atoms
1	C	-0.408670	0.026769
2	C	-0.096223	0.180637
3	H	0.145768	
4	H	0.143904	
5	H	0.145766	
6	H	0.138431	
7	O	-0.259169	-0.259169
8	H	0.138429	
9	C	0.373797	0.373797
10	C	0.153822	0.153822
11	O	-0.365283	-0.365283
12	C	-0.203442	-0.021692
13	O	-0.391716	-0.034213
14	C	0.350651	0.350651
15	O	-0.334989	-0.334989
16	O	-0.265596	-0.265596
17	C	-0.090311	0.177786
18	C	-0.402534	0.017480
19	H	0.134051	
20	H	0.134046	
21	H	0.140691	
22	H	0.138634	
23	H	0.140690	
24	H	0.357503	
25	H	0.181750	

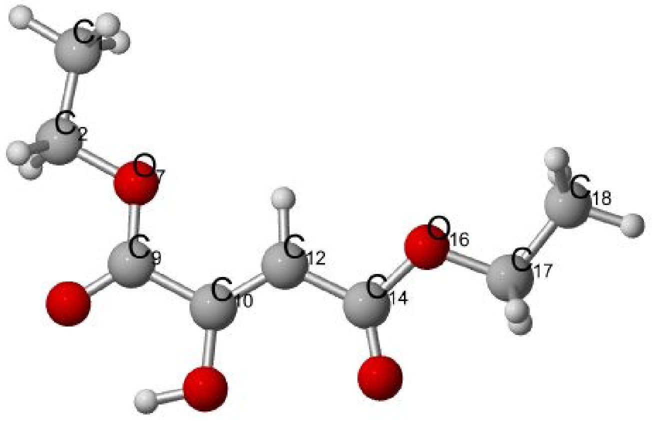
C1
C2
O7
C9
C10
C12
C14
O16
C17
C18
